# Interactions between *Glossina pallidipes* salivary gland hypertrophy virus and tsetse endosymbionts in wild tsetse populations

**DOI:** 10.1186/s13071-022-05536-9

**Published:** 2022-11-29

**Authors:** Mouhamadou M. Dieng, Antonios A. Augustinos, Güler Demirbas-Uzel, Vangelis Doudoumis, Andrew G. Parker, George Tsiamis, Robert L. Mach, Kostas Bourtzis, Adly M. M. Abd-Alla

**Affiliations:** 1 Insect Pest Control Laboratory, Joint FAO/IAEA Centre of Nuclear Techniques in Food and Agriculture, Wagrammer Straße 5, 100, 1400 Vienna, Austria; 2 Laboratory of Systems Microbiology and Applied Genomics, Department of Environmental Engineering, University of Patras, 2 Seferi Str., 30100 Agrinio, Greece; 3 Institute of Chemical, Environmental, and Biological Engineering, Research Area Biochemical Technology, Vienna University of Technology, Gumpendorfer Straße 1a, 1060 Vienna, Austria; 4 Present Address: Department of Plant Protection, Institute of Industrial and Forage Crops, Hellenic Agricultural Organization-Demeter, 26442 Patras, Greece; 5 Present Address: Roppersbergweg 15, 2381 Laab im Walde, Austria

**Keywords:** *Hytrosaviridae*, Tsetse microbiota, Virus transmission, *Wigglesworthia*, *Wolbachia*, *Sodalis*

## Abstract

**Background:**

Tsetse control is considered an effective and sustainable tactic for the control of cyclically transmitted trypanosomosis in the absence of effective vaccines and inexpensive, effective drugs. The sterile insect technique (SIT) is currently used to eliminate tsetse fly populations in an area-wide integrated pest management (AW-IPM) context in Senegal. For SIT, tsetse mass rearing is a major milestone that associated microbes can influence. Tsetse flies can be infected with microorganisms, including the primary and obligate *Wigglesworthia glossinidia*, the commensal *Sodalis glossinidius*, and *Wolbachia pipientis*. In addition, tsetse populations often carry a pathogenic DNA virus, the *Glossina pallidipes* salivary gland hypertrophy virus (GpSGHV) that hinders tsetse fertility and fecundity. Interactions between symbionts and pathogens might affect the performance of the insect host.

**Methods:**

In the present study, we assessed associations of GpSGHV and tsetse endosymbionts under field conditions to decipher the possible bidirectional interactions in different *Glossina* species. We determined the co-infection pattern of GpSGHV and *Wolbachia* in natural tsetse populations. We further analyzed the interaction of both *Wolbachia* and GpSGHV infections with *Sodalis* and *Wigglesworthia* density using qPCR.

**Results:**

The results indicated that the co-infection of GpSGHV and *Wolbachia* was most prevalent in *Glossina austeni* and *Glossina morsitans morsitans*, with an explicit significant negative correlation between GpSGHV and *Wigglesworthia* density. GpSGHV infection levels > 10^3.31^ seem to be absent when *Wolbachia* infection is present at high density (> 10^7.36^), suggesting a potential protective role of *Wolbachia* against GpSGHV.

**Conclusion:**

The result indicates that *Wolbachia* infection might interact (with an undefined mechanism) antagonistically with SGHV infection protecting tsetse fly against GpSGHV, and the interactions between the tsetse host and its associated microbes are dynamic and likely species specific; significant differences may exist between laboratory and field conditions.

**Graphical Abstract:**

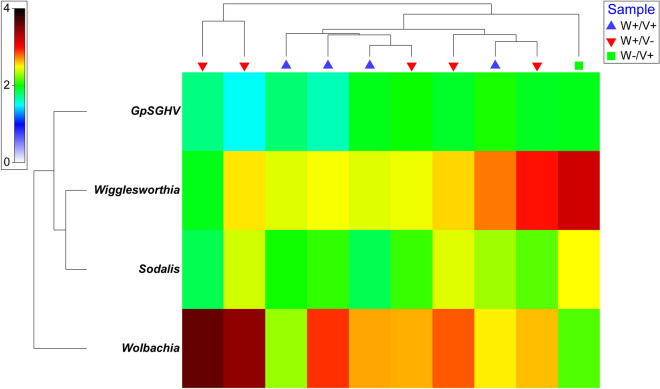

**Supplementary Information:**

The online version contains supplementary material available at 10.1186/s13071-022-05536-9.

## Introduction

Mutualistic bacteria are functionally essential to the physiological well-being of their animal hosts. They benefit their hosts by providing essential nutrients, aiding in digestion and maintaining intestinal equilibrium. Furthermore, mutualistic symbionts foster the development, differentiation, and proper function of their host’s immune system [1–5]. Insects provide a useful model for studying host-microbe interactions because they are associated with bacterial communities that can be easily manipulated during their host’s development [6]. Tsetse flies (*Glossina* spp.) accommodate various types of bacteria, including two gut-associated bacterial symbionts, the obligate *Wigglesworthia glossinidia* and the commensal *Sodalis glossinidius*, the widespread symbiont *Wolbachia pipientis*, and a recently discovered *Spiroplasma* endosymbiont [7–13]. In addition, tsetse flies can house different types of viral infection, including the salivary gland hypertrophy virus (GpSGHV), iflavirus, and negevirus, besides trypanosome parasites [14–17]. Symbiotic associations between insect disease vectors, gut and endosymbiotic bacteria have been particularly well studied to determine how these microbes influence their host’s ability to be infected and transmit disease [18–22]. For example, in tsetse flies, the obligate bacteria *W. glossinidia* are essential for maintaining female fecundity and the host immune system by providing important nutritional components (vitamin B6) and folate (vitamin B9) [22–24]. In addition, *Sodalis* may modulate tsetse susceptibility to infection with trypanosomes, and several studies using field-captured tsetse have noted that the prevalence of trypanosome infections positively correlates with increased *Sodalis* density in the fly’s gut [25–29]. In contrast, the exogenous bacterium *Kosakonia cowanii* inhibits trypanosome infection by creating an unfavorable environment for trypanosome establishment in the mid-gut [30].

Flies in the genus *Glossina* (tsetse flies) are unique to Africa and are of great medical and economic importance as they serve as a vector for the trypanosomes responsible for sleeping sickness in humans (human African trypanosomosis or HAT) and nagana in animals (African animal trypanosomosis or AAT) [31, 32]. The presence of tsetse and trypanosomes is considered one of the major challenges to sustainable development in Africa [33, 34]. The lack of adequate and affordable vaccines coupled with pathogen resistance to drug treatments severely limits AAT control, leaving vector control as the most feasible option for sustainable management of the disease [31, 32]. In addition to various pesticide- and trapping-based methods for tsetse control, the sterile insect technique (SIT) is considered an efficient, sustainable and environmentally friendly method when implemented in the frame of area-wide integrated pest management (AW-IPM) [35, 36]. However, the SIT requires the mass rearing of many males to be sterilized with ionizing radiation before release into the targeted area [33, 37].

Tsetse fly biology is characterized by its viviparous reproduction rendering tsetse mass rearing a real challenge. Tsetse flies nourish their intrauterine larvae from glandular secretions and give birth to fully developed larvae (obligate adenotrophic viviparity) [38, 39]. They also live considerably longer than other vector insects, which somewhat compensates for their slow reproduction rate [40]. The ability to nourish larvae on the milk gland secretion, although limiting the number of larvae produced per female lifetime (8–12), facilitates the transmition of endosymbitic bacteria and pathogens from females to larvae such as *Wigglesworthia*, *Sodalis*, *Wolbachia, Spiroplasma*, and GpSGHV [8, 10, 13, 41]. Moreover, as strictly hematophagous, tsetse rely on the associated endosymbionts to obtain essential nutrients for female reproduction. Therefore, tsetse well-being in mass rearing for SIT is affected by the status of its endosymbionts as well as infection with pathogenic viruses and the interactions between them. Although *Wigglesworthia* is an obligate endosymbiont and found in all tsetse species, *Sodalis*, *Wolbachia*, and *Spiroplasma* infection varied from one species to another [8, 10, 42–45]. In addition, infection with GpSGHV, although reported in different tsetse species, is mainly symptomatic in *G. pallidipes* [46–48]. As GpSGHV is horizontally transmitted via the feeding system under laboratory conditions, leading to high infection rates [49–51], and the virus has a negative effect on the reproductive system of the host causing reduced fecundity and fertility [52, 53], control of the virus infection is important in tsetse mass rearing for efficient production of irradiated males for SIT program implementation.

The variable responses of different tsetse species to the GpSGHV infection might indicate a possibility of the tsetse microbiota modulating the molecular dialogue among the virus, symbiont, and host, shaping the response of each species to the virus infection. It was necessary, therefore, to investigate the infection status of the major tsetse endosymbionts (*Wigglesworthia*, *Sodalis*, and *Wolbachia*) in different tsetse species and their potential interactions. We have recently investigated the interaction between GpSGHV and tsetse symbionts in six tsetse species after virus injection under laboratory conditions [54]. The results indicated that the interaction between the GpSGHV and tsetse symbionts is a complicated process that varies from one tsetse species to another. It is worth noting that the study of Demirbas-Uzel et al. [54] was conducted in tsetse flies maintained under controlled laboratory conditions (sustainable food availability, constant environmental conditions (temperature and humidity), and high density of the flies), which favors the increase of tsetse symbionts [45, 55, 55–57]. In addition, this study was done using adults artificially infected with GpSGHV by injection. Therefore, we investigated the associations of the GpSGHV and tsetse symbionts in field-collected samples by evaluating the prevalence of co-infection of GpSGHV and *Wolbachia* and their potential association with *Wigglesworthia* and *Sodalis* infection in natural tsetse populations. The results are also discussed in the context of developing an effective and robust mass production system of high-quality sterile tsetse flies for implementing SIT programs.

## Methods

### Tsetse samples, extraction of total DNA, and PCR amplifications

The field collection of tsetse fly samples, DNA extraction, and the PCR-based prevalence of GpSGHV and *Wolbachia* infections were reported previously [7, 47, 58, 59]. Based on these publications, and using *G. m. morsitans*, *G. pallidipes*, *G. medicorum*, *G. brevipalpis*, and *G. austeni* samples collected from Burkina Faso, South Africa, Tanzania, Zambia, and Zimbabwe, four infection patterns (i.e. presence) were determined: (i) flies PCR positive for both GpSGHV and *Wolbachia* (*W*^+^/*V*^+^), (ii) flies PCR positive for *Wolbachia* alone (*W*^+^/*V*^−^), (iii) flies PCR positive for GpSGHV alone (*W*^−^/*V*^+^), and (iv) flies PCR negative for both GpSGHV and *Wolbachia* (*W*^−^/*V*^−^). It has to be noted that the prevalence of the symbionts was assessed using a conventional PCR assay while their densities (see below) were determined using a qPCR assay. Since these two assays were different in several aspects including the size of the amplicons and visualization process, this resulted in some discrepancies regarding the infections status of some virus samples initially considered virus free by conventional PCR that were found to be positive during the qPCR analysis.

### Analysis of the associations among SGHV and *Wolbachia*, *Sodalis*, and *Wigglesworthia* infection in wild tsetse populations

The associations among GpSGHV and *Wolbachia, Sodalis*, and *Wigglesworthia* were assessed by qPCR analysis. Tsetse fly samples were selected for qPCR analysis only if a given population of each species was characterized by the presence of two or three of the infection patterns (*W*^+^/*V*^+^), (*W*^+^/*V*^−^), and (*W*^−^/*V*^+^). Based on this criterion, 203 individual flies (78, 103, and 22 flies with infection pattern (*W*^+^/*V*^+^), (*W*^+^/*V*^−^), and (*W*^−^/*V*^+^), respectively) were analyzed (Table 1). The qPCR analysis was performed as previously described [47, 53, 60]. In brief, for the standard curve, total DNA was diluted tenfold before being used for qPCR analysis on a CFX96 real-time PCR detection system (Bio-Rad, Hercules, CA) using the primers and conditions presented in Additional file 2: Table S1. The estimated copy number by qPCR for each sample compared with the standard curve was determined in diluted DNA (4 ng/μl) and corrected through the multiplication by the inverse dilution factor to reflect the GpSGHV, *Wolbachia, Wigglesworthia*, or *Sodalis* copy number (hereafter mention as density) per fly. Analysis of the *Wolbachia*, *Wigglesworthia*, *Sodalis,* and SGHV density levels (titers) was based only on qPCR data with the expected melting curves at 85.5–86 °C, 78.5–80 °C, 81.5–82 °C, and 76.5–77 °C, respectively. Data with a melting curve outside the indicated range were excluded from the analysis. The status of *Sodalis* and *Wigglesworthia* infection of the samples used for the qPCR analysis was not determined by traditional PCR. Based on the estimated copy number per fly for SGHV, *Wolbachia*, *Wigglesworthia*, and *Sodalis*, the average copy number was calculated for all tested flies. Flies with copy number values less than the median were considered infected with low density and flies with copy number value greater than the median were considered infected at a high level. The median copy numbers of the GpSGHV, *Wolbachia*, *Sodalis*, and *Wigglesworthia* in all tested samples were 10^3.31^, 10^7.36^, 10^6.07^, and 10^6.84^ per fly, respectively.

**Table 1:** SGHV and *Wolbachia* infection status of tsetse flies in natural populations of different *Glossina* species

*Glossina* taxon	Country (area, collection date)	*N*	*W*^ + ^/*V*^ +^	*W* ^+^ /*V*^-^	*W*^-^/*V* ^+^	*W*^-^/*V*^-^	χ^2^	*P*
*G. austeni*	Tanzania (Jozani, 1997)^a^	42	0	22	2	18		
*G. austeni*	Tanzania (Zanzibar, 1995)^a,c^	78	3	72	0	3		
*G. austeni*	South Africa (Zululand, 1999)^a,c^	83	51	28	1	3		
*G. austeni*	Coastal Tanzania (Muhoro, NA)	2	0	2	0	0		
*G. austeni*	All locations	205	54	124	3	24	4.32	0.04
*G. brevipalpis*	South Africa (Zululand, 1995)^a^	50	0	1	0	49		
*G. brevipalpis*	Coastal Tanzania (Muhoro, NA)	1	0	1	0	0		
*G. brevipalpis*	Coastal Tanzania (Muyuyu, NA)	1	0	1	0	0		
*G. brevipalpis*	All locations	52	0	3	0	49		
*G. f. fuscipes*	Uganda (Buvuma Island, 1994)^a,b^	53	0	0	6	47		
*G. medicorum*	Burkina Faso (Comoe, 2008)^c^	94	2	18	7	67	0.01	0.94
*G. m. submorsitans*	Burkina Faso (Nazinga, 2009)	3	0	0	0	3		
*G. m. submorsitans*	Burkina Faso (Comoe Folonzo, 2007)	30	0	2	3	25		
*G. m. submorsitans*	Burkina Faso (Comoe, 2008)^c^	109	0	4	9	96		
*G. m. submorsitans*	All locations	142	0	6	12	124	0.58	0.45
*G. p. palpalis*	Democratic Republic of Congo (Zaire, 1995)^a^	48	0	0	1	47		
*G. tachinoides*	Burkina Faso (Nazinga, 2009)	15	0	0	0	15		
*G. tachinoides*	Burkina Faso (Comoe Folonzo, 2007)	112	3	2	26	81		
*G. tachinoides*	Burkina Faso (Comoe, 2008)	72	0	0	8	64		
*G. tachinoides*	Ghana (Pong Tamale, Walewale, 2008)	46	0	5	0	41		
*G. tachinoides*	Ghana (Walewale, 2008)	149	0	27	6	116		
*G. tachinoides*	Ghana (Fumbissi, 2008)	39	0	0	0	39		
*G. tachinoides*	All locations	433	3	34	40	356	0.15	0.70
*G. m. morsitans*	Coastal Tanzania (Utete, NA)	3	0	2	0	1		
*G. m. morsitans*	Zambia (MFWE, Eastern Zambia, 2007)^a,c^	122	26	96	0	0		
*G. m. morsitans*	Tanzania (Ruma, 2005)^a,c^	100	29	71	0	0		
*G. m. morsitans*	Zimbabwe (Gokwe, 2006)^a^	74	0	7	8	59		
*G. m. morsitans*	Zimbabwe (Kemukura, 2006)^a^	26	0	26	0	0		
*G. m. morsitans*	Zimbabwe (M.Chiuy, 1994)^a,c^	36	5	28	0	3		
*G. m. morsitans*	Zimbabwe (Makuti, 2006)^a,c^	99	11	84	1	3		
*G. m. morsitans*	Zimbabwe (Mukond, 1994)^a^	36	0	35	0	1		
*G. m. morsitans*	Zimbabwe (Mushumb, 2006)^a^	8	0	3	0	5		
*G. m. morsitans*	Zimbabwe (Rukomeshi, 2006)^a,c^	100	8	90	0	2		
*G. m. morsitans*	All locations	604	79	442	9	74	1.07	0.30
*G. pallidipes*	Zambia (MFWE, Eastern Zambia, 2007)^a,c^	203	1	4	97	101		
*G. pallidipes*	Kenya (Mewa, Katotoi, Meru national park, 2007)^a^	470	0	0	10	460		
*G. pallidipes*	Ethiopia (Arba Minch, 2007)^a^	454	0	2	87	365		
*G. pallidipes*	Tanzania (Ruma, 2005)^a,c^	83	2	1	42	38		
*G. pallidipes*	Tanzania (Mlembuli and Tunguli, 2009)^a^	94	0	0	0	94		
*G. pallidipes*	Zimbabwe (Mushumb, 2006)^a^	50	0	0	1	49		
*G. pallidipes*	Zimbabwe (Gokwe, 2006)^a^	150	0	0	19	131		
*G. pallidipes*	Zimbabwe (Rukomeshi, 2006)^a^	59	0	5	0	54		
*G. pallidipes*	Zimbabwe (Makuti, 2006)^a,c^	96	1	3	5	87		
*G. pallidipes*	Mainland Tanzania (Death Valley, NA)	6	0	4	0	2		
*G. pallidipes*	Coastal Tanzania (Muhoro, NA)	4	0	3	0	1		
*G. pallidipes*	Coastal Tanzania (Muyuyu, NA)	3	0	3	0	0		
*G. pallidipes*	All locations	1672	4	25	261	1382	0.09	0.76
*G. p. gambiensis*	Senegal (Diacksao Peul and Pout, 2009)^a^	188	0	1	31	156		
*G. p. gambiensis*	Guinea (Kansaba, Mini Pontda, Kindoya, Ghada Oundou, 2009)^a^	180	0	0	13	167		
*G. p. gambiensis*	Guinea (Alahine, 2009)^a^	29	0	0	3	26		
*G. p. gambiensis*	Guinea (Boureya Kolonko, 2009)^a^	36	0	0	1	35		
*G. p. gambiensis*	Guinea (Fefe, 2009)^a^	29	0	0	1	28		
*G. p. gambiensis*	Guinea (Kansaba, 2009)^a^	19	0	0	4	15		
*G. p. gambiensis*	Guinea (Kindoya, 2009)^a^	12	0	1	0	11		
*G. p. gambiensis*	Guinea (Lemonako, 2009)^a^	30	0	0	4	26		
*G. p. gambiensis*	Guinea (Togoue, 2009)^a^	32	0	0	1	31		
*G. p. gambiensis*	Guinea (Conakry, 2010)	138	0	5	0	133		
*G. p. gambiensis*	Burkina Faso (Comoe, 2008)	12	0	0	7	5		
*G. p. gambiensis*	Burkina Faso (Comoe Folonzo, 2007)	53	0	1	14	38		
*G. p. gambiensis*	Burkina Faso (Kenedougou, 2007)	37	0	1	0	36		
*G. p. gambiensis*	Burkina Faso (Houet Bama, 2007)	69	0	1	41	27		
*G. p. gambiensis*	Guinea (Fefe, Togoue, Alahine, Boureya Kolonko, 2009–2010)	94	0	5	0	89		
*G. p. gambiensis*	Guinea (Boureya Kolonko, Kansaba, Kindoya, Ghada Oundou, 2009–2010)	94	0	3	0	91		
*G. p. gambiensis*	Mali (Fijira, 2009)	14	0	0	0	14		
*G. p. gambiensis*	Senegal (Diaka Madia, 2009)	42	0	0	0	42		
*G. p. gambiensis*	Senegal (Tambacounda, 2008)	38	0	3	0	35		
*G. p. gambiensis*	Senegal (Simenti, 2008)	33	0	6	0	27		
*G. p. gambiensis*	Senegal (Kédougou, 2008)	15	0	1	0	14		
*G. p. gambiensis*	All locations	1194	0	28	120	1046	3.20	0.07

^a^In these samples, the presence of *Wolbachia* was tested in Doudoumis et al. [7]

^b^The individuals of *G. f. fuscipes* were considered negative for *Wolbachia* based on the results of the initial PCR amplification. The results from the reamplification method were not considered so that the conditions were consistent for all species

^c^Samples used for qPCR analysis to determine the density of *Wigglesworthia, Sodalis*, *Wolbachia*, and GpSGHV

### Statistical analysis

The proportion of single and double infections (GpSGHV and *Wolbachia*) in wild flies was analyzed by location and species and for all samples together using the Chi-squared test. The Chi-squared tests for independence, Spearman correlation coefficient, and Cochran-Mantel-Haenszel test for repeated tests of independence were performed using Excel 2010. *P*-values were calculated from the data with the significance threshold selected as 0.05.

The difference in *Wigglesworthia, Sodalis, Wolbachia*, and GpSGHV density between different locations and tsetse species and the correlation between densities as well as preparing figures were executed in R v 4.0.5 [61] using RStudio v 1.4.1106 [62, 63] with packages ggplot2 v3.3.2.1 [64], lattice v0.20-41 [65], car (version 3.1-0) [66], ggthems (version 4.2.4) [67], and MASS v7.3-51.6 [68]. All regression analyses of symbionts and GpSGHV densities were conducted using the generalized linear model (glm) for different tsetse species and different countries with analysis of deviance table (type II tests). Pearson correlation coefficient between the density of *Wolbachia* and *Wigglesworthia* and the log transformed density of GpSGHV and *Sodalis* was conducted in R. The analysis details are presented in Additional file 1. Overall similarities in *Wolbachia*, *Wigglesworthia*, *Sodalis*, and GpSGHV density levels between tsetse species, countries, and infection pattern were shown using the matrix display and metric multidimensional scaling (mMDS) plot with bootstrap averages in PRIMER version 7 + and were displayed with a Bray and Curtis matrix based on the square root transformation [69]. The tests were based on the multivariate null hypothesis via the non-parametric statistical method PERMANOVA [70]. The PERMANOVA test was conducted on the average of the qPCR density data based on the country-species sample.

## Results

### Prevalence of co-infection with GpSGHV and *Wolbachia* in wild tsetse flies

Analysis of the *Wolbachia* and GpSGHV infection status for each individual tsetse adult in the previously reported data [7, 47, 58, 59] indicated that the single infection rate was 10.21% (*n* = 459) and 15.12% (*n* = 680) for GpSGHV and *Wolbachia*, respectively, over all taxa and locations combined (Additional file 4: Fig. S1A). No *Wolbachia* infection was found in two taxa, *G. f. fuscipes* and *G. p. palpalis*, and these were excluded from further examination (Table 1). A Cochran-Mantel-Haenszel test for repeated tests of independence showed that infection with GpSGHV and *Wolbachia* did not deviate from independence across all taxa (*χ*^2^_MH_ = 0.848, *df* = 1, n.s.), and individual Chi-squared tests for independence for each taxon did not show any significant deviation from independence at the Bonferroni corrected *α* = 0.00714 (Additional file 3: Table S2). The prevalence of co-infection of GpSGHV and *Wolbachia* (W^+^/V^+^) in wild tsetse populations varied based on the taxon and the location (Table 1 and Additional file 4: Fig. S1B). No co-infection was found in *G. brevipalpis, G. m. submorsitans*, and *G. p. gambiensis*, and co-infection was absent in many locations in the remaining taxa. However, a low prevalence of co-infection was found in *G. medicorum* (2%), *G. tachinoides* (0.7%), and *G. pallidipes* (0.2%). A relatively high prevalence of co-infection was only observed in *G. austeni* (26%) and *G. m. morsitans* (13%) (Additional file 4: Fig. S1B).

### Impact of co-infection (W^+^/V^+^) on GpSGHV, ***Wolbachia***, ***Sodalis***, and ***Wigglesworthia ***density

#### GpSGHV density

The GpSGHV qPCR data showed overall no statistically significant difference between flies with different infection patterns (*W*^+^/*V*^+^), (*W*^−^/*V*^+^), and (*W*^+^/*V*^−^) (*X*^*2*^ = 1.4625, *df* = 2, *P* = 0.481) regardless of tsetse taxon (Additional file 5: Fig. S2A). Moreover, no significant difference in GpSGHV copy number was observed between tsetse taxa (*X*^*2*^ = 0.752, *df* = 3, *P* = 0.861) (Additional file 4: Fig. S1A). However, a significant difference in the virus copy number was observed between different countries (*X*^*2*^ = 16.234, *df* = 4, *P* = 0.0027) where the virus copy number in the flies collected from Zambia was significantly lower than those collected from South Africa, Tanzania, and Zimbabwe (Additional file 1 and 6: Fig. S3A).

#### Wolbachia density

The copy number of *Wolbachia* infection was significantly different between tsetse taxa (*X*^*2*^ = 6.568, *df* = 2, *P* = 0.037) (Additional file 4: Fig. S1B), between the infection statuses (*X*^*2*^ = 23.723, *df* = 2, *P* < 0.001) (Additional file 5: Fig. S2B), and between the countries (*X*^*2*^ = 73.507, *df* = 3, *P* <  < 0.001) (Additional file 6: Fig. S3B). *Wolbachia* density was significantly higher in *G. m. morsitans* than in *G.austeni* (*t* = 2.029, *df* = 1, *P* = 0.0478). (Additional file 1 and 4: Fig. S1B).

Overall, a significant difference in *Wolbachia* density was observed in the flies with different infection patterns previously determined by conventional PCR, where flies with a (*W*^+^/*V*^−^) infection pattern showed significantly higher *Wolbachia* density than flies with a (*W*^+^/*V*^+^) infection pattern regardless of the tsetse species (*X*^*2*^ = 23.723, *df* = 2, *P* <  < 0.001). This trend was observed in *G. m. morsitans* (*t* = 3.184, *P* = 0.0022) (Additional file 1). The *Wolbachia* density was highest in the flies collected from Zambia (Additional file 6: Fig. S3B). Analyzing only the flies with co-infection (*W*^+^/*V*^+^) indicated that the *Wolbachia* density was statistically significantly higher in *G. m. morsitans* than in *G.austeni* (*t* = − 2.353, *df* = 1, *P* = 0.024) (Additional file 1 and 7: Fig. S4B).

#### Interaction between GpSGHV infection and *Wolbachia*, *Wigglesworthia*, and *Sodalis* infection

The qPCR results of both *Wigglesworthia* and *Sodalis* in tsetse adults with different infection patterns (*W*^+^/*V*^+^), (*W*^−^/*V*^+^), and (*W*^+^/*V*^−^) indicated that *Wigglesworthia* density varies significantly between different infection patterns (*X*^*2*^ = 10.706, *df* = 2, *P* = 0.0047) and its density in flies with co-infection (*W*^+^/*V*^+^) was significantly lower than in those with *Wolbachia* infection only (*W*^+^/*V*^−^) (*t* = 3.137, *df* = 2, *P* = 0.0024) but did not differ significantly from flies with virus infection only (*W*^−^/*V*^+^) (*t* = 1.656, *P* = 0.102) (Additional file 5: Fig. S2C). *Wigglesworthia* density varies also between tsetse taxa (*X*^*2*^ = 33.479, *df* = 4, *P* <  < 0.001) with higher density in *G. m. morsitans* and *G.pallidipes* than in *G. austeni* (Additional file 4: Fig. S1C) as well as between countries (*X*^*2*^ = 19.785, *df* = 3, *P* < 0.001) (Additional file 1 and 6: Fig. S3C).

**Fig. 1:**
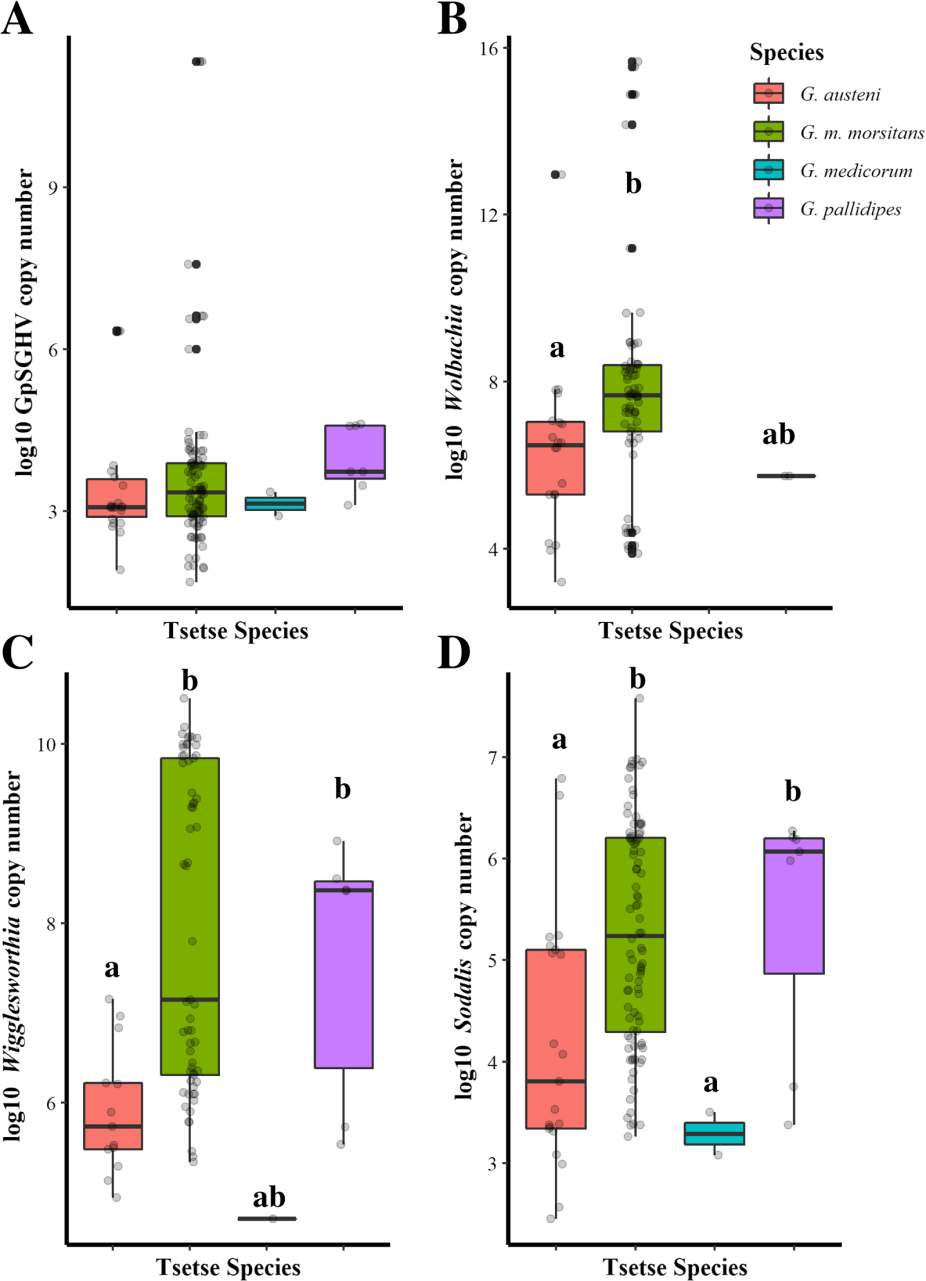
Density levels of GpSGHV (**A**), *Wolbachia* (**B**), *Wigglesworthia* (**C**), and *Sodalis* (**D**) in different tsetse species. The copy number was determined by qPCR. Values indicated by a different small letters differ significantly at the 5% level

*Sodalis* density also varies between tsetse taxa (*X*^*2*^ = 21.612, *df* = 3, *P* < 0.001) (Fig. 1D) and between countries (*X*^*2*^ = 21.179, *df* = 4, *P* < 0.001) (Additional file 6: Fig. S3D) but there was no significant difference between tsetse flies with different infection patterns (*X*^*2*^ = 0.63888, *df* = 2, *P* = 0.727) (Additional file 1 and 5: Fig. S2D).

**Fig. 2:**
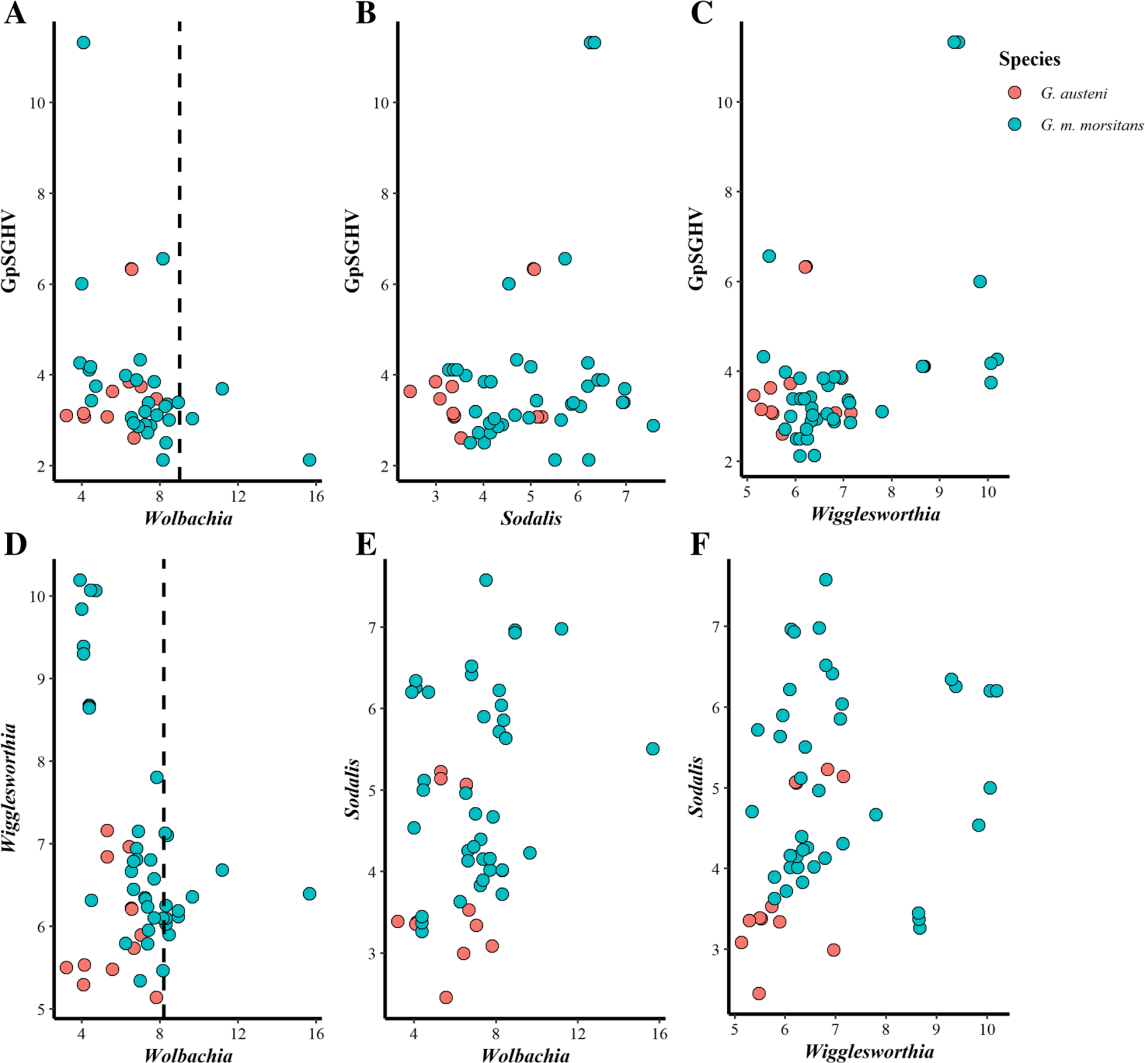
Interaction between the GpSGHV and tsetse endosymbionts *Wigglesworthia, Wolbachia*, and *Sodalis* in natural populations of *G. austeni* and *G. m. morsitans*. The density of tsetse symbionts was analyzed by qPCR, and the data of each two organisms were plotted in R. The density of GpSGHV was plotted versus the density of *Wolbachia* (**A**), *Sodalis* (**B**), and *Wigglesworthia* (**C**). The density of *Wigglesworthia* was plotted versus *Wolbachia* (**D**), and the density of *Sodalis* was plotted versus *Wolbachia* (**E**) and *Wigglesworthia* (**F**). Vertical bar A and D indicates the *Wolbachia* density at 10^9^ and 10^8.2^ copy number, respectively

Analyzing the pairwise correlation between the GpSGHV and each of the tsetse endosymbionts in *G. austeni* and *G. m. morsitans* (species with the highest number of flies with co-infection) indicated different types of correlation based on the insect taxa. In *G. m. morsitans*, the GpSGHV density has a significant negative correlation with *Wolbachia* density (*r* = − 0. 558, *t* = − 4.150, *df* = 38, *P* < 0.001). No flies were observed with high virus density (> 10^3.3^ copy number) when *Wolbachia* density was high (~ 10^7.3^ copy number), although this observation should be considered with caution as it is based on a small sample size. Contrary to *Wolbachia*, GpSGHV has a significant positive correlation with *Wigglesworthia* (*r* = 0.531, *t* = 3.868, *df* = 38, *P* < 0.001) but no correlation with *Sodalis* density (*r* = 0.203, *t* = 1.276, *df* = 38, *P* = 0.209). *Wolbachia* density also showed significant negative correlation with *Wigglesworthia* density (*r* = − 0.637, *t* = − 5.095, *df* = 38, *P* < 0.001). No flies with high *Wigglesworthia* density (~ 10^8^ copy number) were detected when *Wolbachia* density was high (> 10^7.3^ copy number). In contrast, *Sodalis* density did not show significant correlation with either *Wolbachia* (*r* = 0.193, *t* = 1.214, *df* = 38, *P* = 0.232) or *Wigglesworthia* densities (*r* = 0.072, *t* = 0.443, *df* = 38, *P* = 0.66) (Fig. 2, Additional file 1). In *G. austeni*, the only significant correlation was found to be positive between *Sodalis* and *Wigglesworthia* density (*r* = 0.602, *t* = 2.386, *df* = 10, *P* = 0.038) (Fig. 2, Additional file 1).

**Fig. 3:**
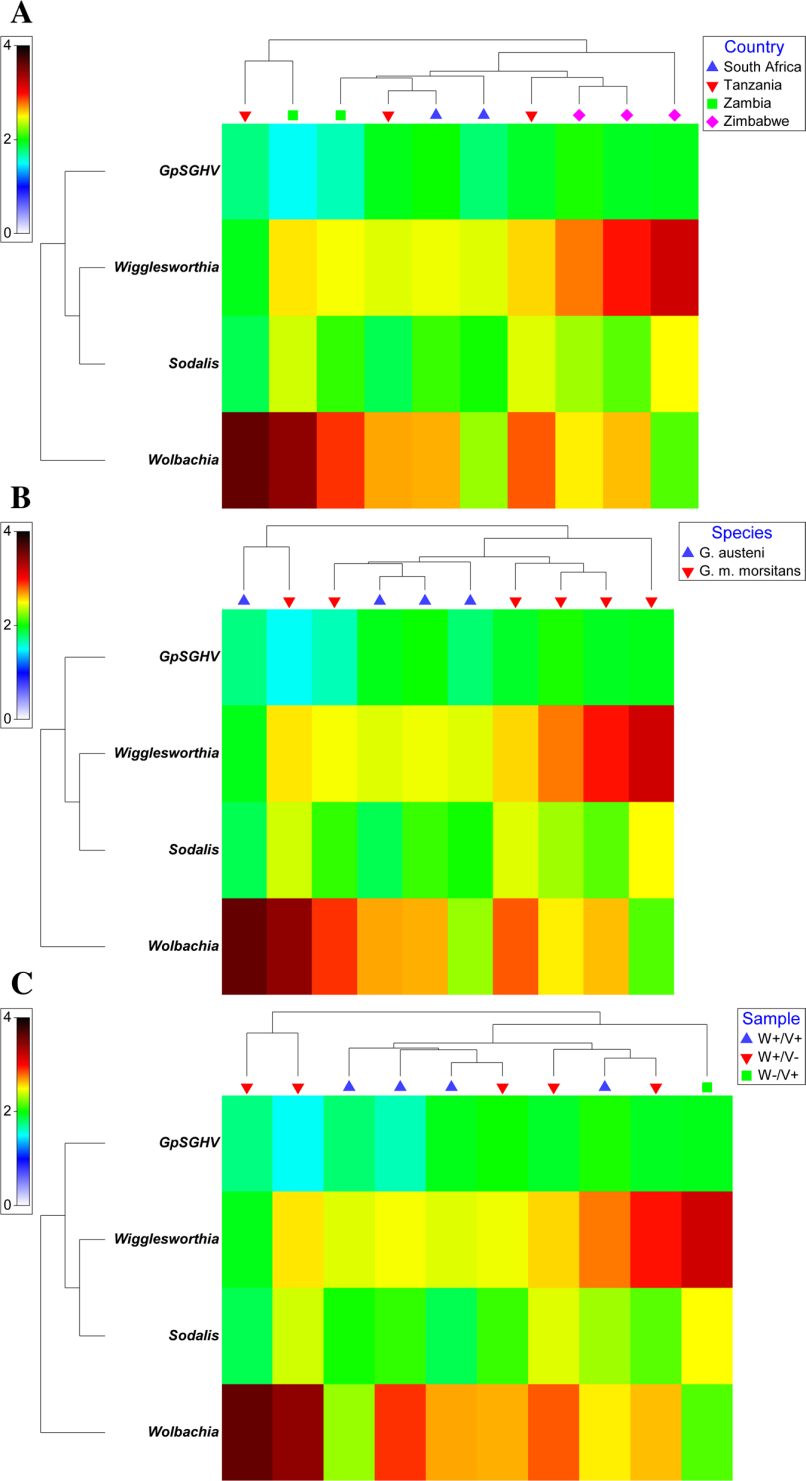
Relative density of GpSGHV, *Wigglesworthia*, *Sodalis*, and *Wolbachia* in *G. austeni* and *G. m. morsitans* field-collected tsetse flies. The density of GpSGHV and tsetse symbionts was analyzed by qPCR. Data were transformed to square root and averaged based on country (**A**), tsetse species (**B**), and infection status (Sample) (**C**). The top and the left of the graph indicate the group averaged Bray-Curtis similarity

**Fig. 4:**
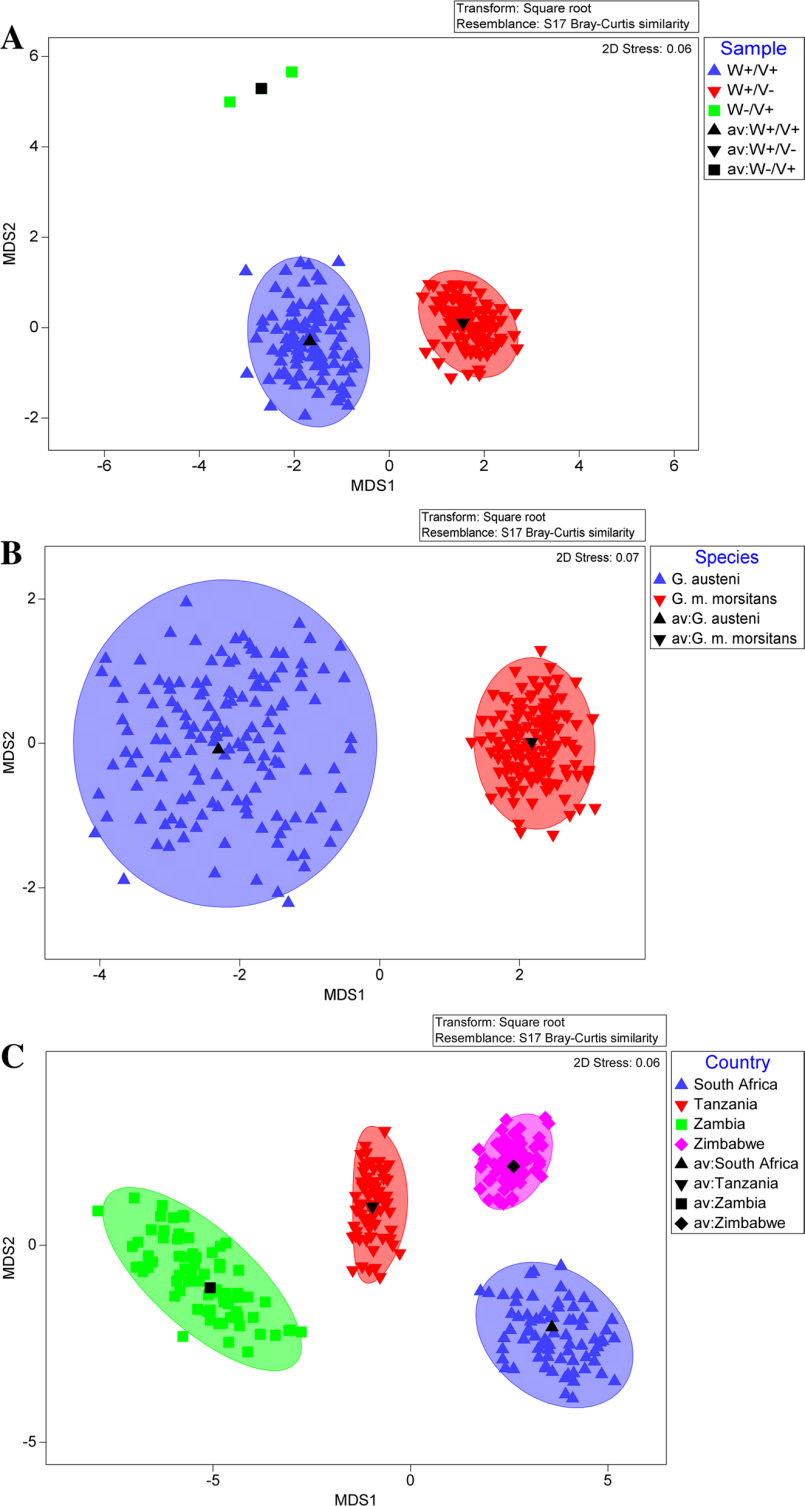
Metric multidimensional scaling (mMDS) of GpSGHV, *Wigglesworthia, Sodalis*, and *Wolbachia* relative density in field-collected tsetse flies. The mMDS of GpSGHV, *Wigglesworthia, Sodalis*, and *Wolbachia* relative density was performed in respect to infection status (Sample) (**A**), tsetse species (**B**), or country (**C**). *av *average

The qPCR results showed that *Wolbachia*-infected flies had relatively high *Wolbachia* density (median 10^7.3^ copies/fly) compared to the GpSGHV and other tsetse symbionts (*Wigglesworthia* and *Sodalis*) regardless of the species, country, or infection pattern (Fig. 3). The heat map analysis of the qPCR data of *G. austeni* and *G. m. morsitans* clearly indicates the contrast between *Wolbachia* copy number and *Wigglesworthia* copy number considering the infection pattern, tsetse taxa, or countries. In addition, it clearly shows the low copy number of GpSGHV in the samples showing a high *Wolbachia* copy number (Fig. 3, Additional file 8:Fig. S5). The bootstrap averages of the metric multidimensional scaling (mMDS) produced clusters based on the species, country, and infection pattern (Fig. 4). The PERMANOVA analysis of the density of GpSGH, *Wolbachia*, *Wigglesworthia*, and *Sodalis* based on the country, tsetse species, and infection pattern indicated that the clusters observed between infection pattern (*P* = 0.026) and country (*P* = 0.001) were statistically significant. The interaction between country and infection pattern was not statistically significant (*P* = 0.123) (Table 2).

**Table 2:** PERMANOVA table of results for country and infection status factors and their combinations

Source	*df*	SS	MS	Pseudo-F	P(perm)	Uniqueperms
Country	2	657.53	328.77	13.003	**0.001**	999
Species	0	0		No test		
Infection status	2	188.97	94.487	3.7369	**0.026**	998
Country × species	0	0		No test		
Country × infection status	2	100.59	50.296	1.9892	0.123	999
Species × infection status	0	0		No test		
Country × species × infection status	0	0		No test		
Res	104	2629.6	25.285			
Total	113	4681.3				

Within the table, statistically significant differences (*P* < 0.05) are shown in bold

*Perm(s)* permutations

## Discussion

The prevalence of GpSGHV and *Wolbachia* in natural tsetse populations clearly indicated that the two infections were independent (not correlated) in most of the tested tsetse species with only *G. m. morsitans* and *G. austeni* presenting a high proportion of co-infections. However, the number of co-infections originally determined by conventional PCR may have been underestimated with conventional PCR as the qPCR analysis carried out in the frame of the present study clearly indicated that a number of initially considered virus-free samples were found to be positive, albeit at low density. It should also be noted that the *Wolbachia* strains infecting *G. m. morsitans* and *G. austeni* are closely related but different, as has been shown by both MLST analysis and, more recently, genome sequencing [7, 40, 71].

Analysis of *G. morsitans* and *G. austeni* co-infected samples suggested that low density of GpSGHV is associated with high density of *Wolbachia*. Due to the low number of individuals showing this correlation, further analysis is required. Moreover, the screen of wild tsetse populations for GpSGHV and *Wolbachia* infection indicated that not all *Glossina* species harbor *Wolbachia* or GpSGHV. Furthermore, *Wolbachia* and GpSGHV prevalence was found to differ not only between different tsetse host species but also between different populations within the same tsetse species [7, 11, 47, 58, 59, 72].

The potential negative impact (antagonistic effect) of *Wolbachia* density on the GpSGHV density in natural tsetse populations is in agreement with the recent report on the interaction of *Wolbachia* and GpSGHV infection in colonized tsetse populations [54]. However, the number of tested flies was not equally distributed between the tsetse taxa and locations, which might explain the lack of detected co-infections in some taxa and, therefore, the low number of taxa (*G. austeni* and *G. m. morsitans*) used for investigating the interactions between the GpSGHV and tsetse symbionts. The negative correlation between *Wolbachia* and GpSGHV infections was also reported in wild-caught *G. f. fuscipes* collected from Uganda [72]. This conflicts with our findings as no *G. f. fuscipes* flies with GpSGHV were reported, which might be due to the low number of tested flies used in our study (*n* = 53).

Several reports have discussed and well documented the negative effect of *Wolbachia* on RNA viruses in different insect models such as mosquitoes and *Drosophila* [73–75], although there have also been reports about *Wolbachia* enhancement of both RNA and DNA viruses [76–78]. It is worth mentioning that the negative correlation of *Wolbachia* with GpSGHV was observed only when *Wolbachia* density was high as the results show the absence of high density (> 10^3.7^) GpSGHV infection with high-density *Wolbachia* infection (> 10^7.5^). However, at low *Wolbachia* density co-infection occurs with a prevalence of > 10%. Although our study indicated a correlation between high-density *Wolbachia* and low-density GpSGHV, previous reports suggested that the negative impact of *Wolbachia* on insect viruses is density dependent [76, 79].

The assessment of the infection density (copy number per fly) of all four microbes (GpSGHV, *Wolbachia*, *Wigglesworthia*, and *Sodalis*) in the same tsetse flies indicated that *Wolbachia* infection at high density has a significant negative correlation with *Wigglesworthia* infection in *G. m. morsitans* but not in *G. austeni*. However, the latter might be due to the low number of analyzed *G. austeni* flies (*n* = 21) compared to *G. m. morsitans* (*n* = 91). On the other hand, *Wolbachia* density levels do not correlate with *Sodalis*. The nature of the negative interaction between *Wolbachia* and *Wigglesworthia* is unclear. Whether this negative correlation between *Wolbachia* and *Wigglesworthia* is present in other tsetse species beyond *G. m. morsitans* remains to be seen.

The positive correlation between GpSGHV infection and *Wigglesworthia* infection observed in *G. m. morsitans* conflicts with the negative correlation observed in the same species of colonized flies [54]. This result might reflect a specific adaptation between a specific strain of *Wigglesworthia*, which reacts in a specific way to increase its density in the presence of GpSGHV as a manner to restore and enhance the host immune system against the virus infection [80]. The difference in the interaction between the GpSGHV and *Wigglesworthia* between the results of this study and the results of Demirbas-Uzel et al. [54] might be due to: (i) difference in the host strain/genotype as the *G. m. morsitans* individuals were collected from several countries in east Africa (Tanzania, Zambia, and Zimbabwe) while the colonized flies originated from Zimbabwe and have been maintained in the colony since 1997; (ii) different strain(s) of *Wigglesworthia* circulating in the field samples compared to the ones present in colonized flies [60]; (iii) different strain(s) of the GpSGHV in the field samples [58]; (iv) difference between field and laboratory conditions where the stress from handling the large number of flies in high density in the laboratory might negatively affect *Wigglesworthia* density levels and/or performance. The same reasons may also explain the difference observed between field and laboratory samples regarding the interactions between GpSGHV and *Sodalis*.

## Conclusions

The present study, despite its limitations regarding the size of samples and the lack of knowledge about the age, nutritional and trypanosome infection status, and environmental conditions at the time of collection of field specimens, shows a snapshot image of the density levels of tsetse symbionts and SGHV under field conditions and clearly indicates that the interactions/association between the tsetse host and its associated microbes are dynamic and likely species specific, and significant differences may exist between laboratory and field conditions. Further studies are needed to clarify the interaction between tsetse symbionts and GpSGHV under field conditions.

## Supplementary Information

**Additional file 1**: Interactions between tsetse endosymbionts and *Glossina pallidipes* salivary gland hypertrophy virus in wild tsetse populations.

**Additional file 2**: **Table S1.** List of primers used for quantitative PCR (qPCR) analyses in *Glossina* species.

**Additional file 3**: **Table S2.** Cochran-Mantel-Haenszel test for repeated tests of independence with continuity correction on the coingection of GpSGHV and *Wolbachia* in wild tsetse species.

**Additional file 4**: **Figure S1.** Prevalence of GpSGHV and *Wolbachia* co-infection in natural tsetse populations. A: In all tsetse species; B: in each tsetse species. GpSGHV and *Wolbachia* prevalence was determined by PCR as described previously [7,47].

**Additional file 5**: **Figure S2.** Density levels of GpSGHV (A), *Wolbachia* (B), *Wigglesworthia* (C), and *Sodalis* (D) determined by qPCR in tsetse flies with different GpSGHV and *Wolbachia* infection statuses. The copy number was determined by qPCR. Values indicated by a different small letter differ significantly at the 5% level. W^+^/V^+^: flies infected with both *Wolbachia* and GpSGHV; W^+^/V^-^: flies infected only with *Wolbachia*; W^-^/V^-+^: flies infected only with GpSGHV. GpSGHV and *Wolbachia* infection status was determined by conventional PCR as described previously [7,47].

**Additional file 6**: **Figure S3.** Density levels of GpSGHV (A), *Wolbachia* (B), *Wigglesworthia* (C), and *Sodalis* (D) in tsetse flies collected from different countries. The copy number was determined by qPCR. Values indicated by a different small letter differ significantly at the 5% level.

**Additional file 7: Figure S4.** Impact of GpSGHV and *Wolbachia *co-infection (W^+^/V^+^) on the density levels of GpSGHV (A), *Wolbachia* (B), *Wigglesworthia* (C), and *Sodalis* (D) in different tsetse species. The copy number was determined by qPCR. Values indicated by the same lower case letter do not differ significantly at the 5% level.

**Additional file 8: Figure S5.** Relative density of GpSGHV, *Wigglesworthia*, *Sodalis*, and *Wolbachia* in *G. austeni * and *G. m. morsitans* field-collected tsetse flies. The density of GpSGHV and tsetse symbionts was analyzed by qPCR. Data were transformed to square root and averaged based on countries and species (A), countries and infection status (sample) (B), and countries, species, and infection status (sample) (C). The top and the left of the graph indicate the group averaged Bray-Curtis similarity.

## Data Availability

Materials described in the paper, including all relevant raw data, are available in this link. https://dataverse.harvard.edu/dataset.xhtml?persistentId=doi:10.7910/DVN/X15PQF
