# Supplementary material for: Interactions between Glossina pallidipes salivary gland hypertrophy virus and tsetse endosymbionts in wild tsetse populations
Source: Parasit Vectors. 2022 Nov 29;15:447. doi: 10.1186/s13071-022-05536-9 (PMC9707009; doi:10.1186/s13071-022-05536-9)
Supplement: Supplementary file 1 — Additional file 1: Interactions between tsetse endosymbionts and Glossina pallidipes salivary gland hypertrophy virus in wild tsetse populations. [file 13071_2022_5536_MOESM1_ESM.docx]

Interactions between tsetse endosymbionts and Glossina pallidipes Salivary Gland Hypertrophy Virus in wild tsetse populations

Mouhamadou M. Dieng, Antonios A. Augustinos, Güler Demirbas-Uzel, Vangelis Doudoumis, Andrew G. Parker, George Tsiamis, Robert L. Mach, Kostas Bourtzis, and Adly M. M. Abd-Alla

18/06/2021

## Set up working directory and load needed library

setwd("C:/Users/abdallaa/OneDrive - IAEA/My_passport_6/Dieng_M/Virus_ymbiont_filed/raw_data2")

library(ggplot2)

## Warning: package 'ggplot2' was built under R version 4.1.3

library(gcookbook)
library(ggfortify)

## Warning: package 'ggfortify' was built under R version 4.1.3

library(datasets)
library(MASS)

## Warning: package 'MASS' was built under R version 4.1.3

library(survival)

## Warning: package 'survival' was built under R version 4.1.3

library(rmarkdown)

## Warning: package 'rmarkdown' was built under R version 4.1.3

library(knitr)

## Warning: package 'knitr' was built under R version 4.1.3

library(coxme)

## Loading required package: bdsmatrix

##
## Attaching package: 'bdsmatrix'

## The following object is masked from 'package:base':
##
## backsolve

library(lme4)

## Warning: package 'lme4' was built under R version 4.1.3

## Loading required package: Matrix

library(nlme)

## Warning: package 'nlme' was built under R version 4.1.3

##
## Attaching package: 'nlme'

## The following object is masked from 'package:lme4':
##
## lmList

library(lattice)
library(MuMIn)

## Warning: package 'MuMIn' was built under R version 4.1.3

## Registered S3 methods overwritten by 'MuMIn':
## method from
## formula.coxme coxme
## logLik.coxme coxme
## logLik.lmekin coxme

#install.packages("ggthemes") # Install
library(ggthemes) # Load
#install.packages("ggtext") # Install
library(ggtext) # Load
library(car)

## Warning: package 'car' was built under R version 4.1.3

## Loading required package: carData

## Warning: package 'carData' was built under R version 4.1.3

## Preparing figure 1 and its statistics

fig1 <- read.csv("virus_symb_all_20210613_all3.csv")
head(fig1)

## Country Species Samples GpSGHV Wolbachia Sodalis Wigglesworthia
## 1 South Africa G. austeni W+/V+ 3.071882 5.291369 5.226169 6.837400
## 2 South Africa G. austeni W+/V+ 3.631444 5.563006 2.453318 5.479647
## 3 South Africa G. austeni W+/V+ 3.070407 4.121888 3.376584 5.528074
## 4 South Africa G. austeni W+/V+ 3.071882 5.291369 5.138430 7.157453
## 5 South Africa G. austeni W+/V- 6.330414 6.542078 5.056997 6.218625
## 6 South Africa G. austeni W+/V- 6.322219 6.544068 5.070590 6.208117

summary(fig1)

## Country Species Samples GpSGHV
## Length:123 Length:123 Length:123 Min. : 1.685
## Class :character Class :character Class :character 1st Qu.: 2.917
## Mode :character Mode :character Mode :character Median : 3.313
## Mean : 3.672
## 3rd Qu.: 3.869
## Max. :11.321
##
## Wolbachia Sodalis Wigglesworthia
## Min. : 3.202 Min. :2.453 Min. : 4.708
## 1st Qu.: 6.412 1st Qu.:4.042 1st Qu.: 6.096
## Median : 7.360 Median :5.066 Median : 6.837
## Mean : 7.493 Mean :5.058 Mean : 7.538
## 3rd Qu.: 8.312 3rd Qu.:6.188 3rd Qu.: 9.343
## Max. :15.659 Max. :7.576 Max. :10.506
## NA's :30 NA's :1 NA's :42

fig1a<-ggplot(fig1,aes(x=Species,y=Wolbachia, fill=Species)) +
 geom_boxplot() + geom_jitter(width=0.1,alpha=0.2)

tiff("fig1a.tiff", width = 4, height = 4, units = 'in', res = 300)
plot(fig1a+theme_tufte() + theme(axis.line = element_line(size = 1, colour = "black")) + theme(legend.title = element_text(face = "bold")) + theme(legend.text = element_text(face = "italic"))) + theme(axis.text.x = element_blank()) + xlab(expression(bold("Tsetse Species"))) + ylab(expression (paste (bold("log10 "), bolditalic("Wolbachia"), bold(" copy number"))))

## Warning: Removed 30 rows containing non-finite values (stat_boxplot).

## Warning: Removed 30 rows containing missing values (geom_point).

## Warning: Removed 30 rows containing non-finite values (stat_boxplot).

## Warning: Removed 30 rows containing missing values (geom_point).

dev.off()

## png
## 2

#------------------------------------------------------------------

fig1b<-ggplot(fig1,aes(x=Species,y=Sodalis, fill=Species)) +
 geom_boxplot() + geom_jitter(width=0.1,alpha=0.2)

tiff("fig1b.tiff", width = 4, height = 4, units = 'in', res = 300)
plot(fig1b+theme_tufte() + theme(axis.line = element_line(size = 1, colour = "black")) + theme(legend.title = element_text(face = "bold")) + theme(legend.text = element_text(face = "italic"))) + theme(axis.text.x = element_blank()) + xlab(expression(bold("Tsetse Species"))) + ylab(expression (paste (bold("log10 "), bolditalic("Sodalis"), bold(" copy number"))))

## Warning: Removed 1 rows containing non-finite values (stat_boxplot).

## Warning: Removed 1 rows containing missing values (geom_point).

## Warning: Removed 1 rows containing non-finite values (stat_boxplot).

## Warning: Removed 1 rows containing missing values (geom_point).

dev.off()

## png
## 2

#------------------------------------------------------------------

fig1c<-ggplot(fig1,aes(x=Species,y=Wigglesworthia, fill=Species)) +
 geom_boxplot() + geom_jitter(width=0.1,alpha=0.2)

tiff("fig1c.tiff", width = 4, height = 4, units = 'in', res = 300)
plot(fig1c+theme_tufte() + theme(axis.line = element_line(size = 1, colour = "black")) + theme(legend.title = element_text(face = "bold")) + theme(legend.text = element_text(face = "italic"))) + theme(axis.text.x = element_blank()) + xlab(expression(bold("Tsetse Species"))) + ylab(expression (paste (bold("log10 "), bolditalic("Wigglesworthia"), bold(" copy number"))))

## Warning: Removed 42 rows containing non-finite values (stat_boxplot).

## Warning: Removed 42 rows containing missing values (geom_point).

## Warning: Removed 42 rows containing non-finite values (stat_boxplot).

## Warning: Removed 42 rows containing missing values (geom_point).

dev.off()

## png
## 2

#------------------------------------------------------------------

fig1d<-ggplot(fig1,aes(x=Species,y=GpSGHV, fill=Species)) +
 geom_boxplot() + geom_jitter(width=0.1,alpha=0.2)

tiff("fig1d.tiff", width = 4, height = 4, units = 'in', res = 300)
plot(fig1d+theme_tufte() + theme(axis.line = element_line(size = 1, colour = "black")) + theme(legend.title = element_text(face = "bold")) + theme(legend.text = element_text(face = "italic"))) + theme(axis.text.x = element_blank()) + xlab(expression(bold("Tsetse Species"))) + ylab(expression (bold("log10 GpSGHV copy number ")))
dev.off()

## png
## 2

#----------------------------------------------------
#statistics for Figure 1

wol <- glm (Wolbachia~Species, data = fig1)
summary(wol)

##
## Call:
## glm(formula = Wolbachia ~ Species, data = fig1)
##
## Deviance Residuals:
## Min 1Q Median 3Q Max
## -3.9776 -1.0464 -0.1582 0.5495 7.7915
##
## Coefficients:
## Estimate Std. Error t value Pr(>|t|)
## (Intercept) 6.3378 0.5705 11.109 <2e-16 ***
## SpeciesG. m. morsitans 1.5297 0.6459 2.368 0.020 *
## SpeciesG. pallidipes -0.5927 1.8922 -0.313 0.755
## ---
## Signif. codes: 0 '***' 0.001 '**' 0.01 '*' 0.05 '.' 0.1 ' ' 1
##
## (Dispersion parameter for gaussian family taken to be 6.510038)
##
## Null deviance: 628.66 on 92 degrees of freedom
## Residual deviance: 585.90 on 90 degrees of freedom
## (30 observations deleted due to missingness)
## AIC: 443.09
##
## Number of Fisher Scoring iterations: 2

Anova(wol)

## Analysis of Deviance Table (Type II tests)
##
## Response: Wolbachia
## LR Chisq Df Pr(>Chisq)
## Species 6.5678 2 0.03748 *
## ---
## Signif. codes: 0 '***' 0.001 '**' 0.01 '*' 0.05 '.' 0.1 ' ' 1

wol <- glm (Wolbachia~Samples, data = fig1)
summary(wol)

##
## Call:
## glm(formula = Wolbachia ~ Samples, data = fig1)
##
## Deviance Residuals:
## Min 1Q Median 3Q Max
## -4.6581 -1.3175 -0.4131 1.0681 7.1111
##
## Coefficients:
## Estimate Std. Error t value Pr(>|t|)
## (Intercept) 4.604 1.663 2.769 0.00683 **
## SamplesW+/V- 3.944 1.695 2.327 0.02219 *
## SamplesW+/V+ 1.688 1.704 0.991 0.32428
## ---
## Signif. codes: 0 '***' 0.001 '**' 0.01 '*' 0.05 '.' 0.1 ' ' 1
##
## (Dispersion parameter for gaussian family taken to be 5.527992)
##
## Null deviance: 628.66 on 92 degrees of freedom
## Residual deviance: 497.52 on 90 degrees of freedom
## (30 observations deleted due to missingness)
## AIC: 427.89
##
## Number of Fisher Scoring iterations: 2

Anova(wol)

## Analysis of Deviance Table (Type II tests)
##
## Response: Wolbachia
## LR Chisq Df Pr(>Chisq)
## Samples 23.723 2 7.057e-06 ***
## ---
## Signif. codes: 0 '***' 0.001 '**' 0.01 '*' 0.05 '.' 0.1 ' ' 1

fig1$Species=as.factor(fig1$Species)

fig1$Species <- relevel(fig1$Species, ref = "G. austeni")
wol <- glm (Wolbachia~Species, data = fig1)
summary(wol)

##
## Call:
## glm(formula = Wolbachia ~ Species, data = fig1)
##
## Deviance Residuals:
## Min 1Q Median 3Q Max
## -3.9776 -1.0464 -0.1582 0.5495 7.7915
##
## Coefficients:
## Estimate Std. Error t value Pr(>|t|)
## (Intercept) 6.3378 0.5705 11.109 <2e-16 ***
## SpeciesG. m. morsitans 1.5297 0.6459 2.368 0.020 *
## SpeciesG. pallidipes -0.5927 1.8922 -0.313 0.755
## ---
## Signif. codes: 0 '***' 0.001 '**' 0.01 '*' 0.05 '.' 0.1 ' ' 1
##
## (Dispersion parameter for gaussian family taken to be 6.510038)
##
## Null deviance: 628.66 on 92 degrees of freedom
## Residual deviance: 585.90 on 90 degrees of freedom
## (30 observations deleted due to missingness)
## AIC: 443.09
##
## Number of Fisher Scoring iterations: 2

fig1$Species <- relevel(fig1$Species, ref = "G. medicorum")
wol <- glm (Wolbachia~Species, data = fig1)
summary(wol)

##
## Call:
## glm(formula = Wolbachia ~ Species, data = fig1)
##
## Deviance Residuals:
## Min 1Q Median 3Q Max
## -3.9776 -1.0464 -0.1582 0.5495 7.7915
##
## Coefficients:
## Estimate Std. Error t value Pr(>|t|)
## (Intercept) 6.3378 0.5705 11.109 <2e-16 ***
## SpeciesG. m. morsitans 1.5297 0.6459 2.368 0.020 *
## SpeciesG. pallidipes -0.5927 1.8922 -0.313 0.755
## ---
## Signif. codes: 0 '***' 0.001 '**' 0.01 '*' 0.05 '.' 0.1 ' ' 1
##
## (Dispersion parameter for gaussian family taken to be 6.510038)
##
## Null deviance: 628.66 on 92 degrees of freedom
## Residual deviance: 585.90 on 90 degrees of freedom
## (30 observations deleted due to missingness)
## AIC: 443.09
##
## Number of Fisher Scoring iterations: 2

fig1$Species <- relevel(fig1$Species, ref = "G. m. morsitans")
wol <- glm (Wolbachia~Species, data = fig1)
summary(wol)

##
## Call:
## glm(formula = Wolbachia ~ Species, data = fig1)
##
## Deviance Residuals:
## Min 1Q Median 3Q Max
## -3.9776 -1.0464 -0.1582 0.5495 7.7915
##
## Coefficients:
## Estimate Std. Error t value Pr(>|t|)
## (Intercept) 7.8674 0.3028 25.982 <2e-16 ***
## SpeciesG. austeni -1.5297 0.6459 -2.368 0.020 *
## SpeciesG. pallidipes -2.1224 1.8294 -1.160 0.249
## ---
## Signif. codes: 0 '***' 0.001 '**' 0.01 '*' 0.05 '.' 0.1 ' ' 1
##
## (Dispersion parameter for gaussian family taken to be 6.510038)
##
## Null deviance: 628.66 on 92 degrees of freedom
## Residual deviance: 585.90 on 90 degrees of freedom
## (30 observations deleted due to missingness)
## AIC: 443.09
##
## Number of Fisher Scoring iterations: 2

fig1$Species <- relevel(fig1$Species, ref = "G. pallidipes")
wol <- glm (Wolbachia~Species, data = fig1)
summary(wol)

##
## Call:
## glm(formula = Wolbachia ~ Species, data = fig1)
##
## Deviance Residuals:
## Min 1Q Median 3Q Max
## -3.9776 -1.0464 -0.1582 0.5495 7.7915
##
## Coefficients:
## Estimate Std. Error t value Pr(>|t|)
## (Intercept) 5.7451 1.8042 3.184 0.00199 **
## SpeciesG. m. morsitans 2.1224 1.8294 1.160 0.24906
## SpeciesG. austeni 0.5927 1.8922 0.313 0.75483
## ---
## Signif. codes: 0 '***' 0.001 '**' 0.01 '*' 0.05 '.' 0.1 ' ' 1
##
## (Dispersion parameter for gaussian family taken to be 6.510038)
##
## Null deviance: 628.66 on 92 degrees of freedom
## Residual deviance: 585.90 on 90 degrees of freedom
## (30 observations deleted due to missingness)
## AIC: 443.09
##
## Number of Fisher Scoring iterations: 2

#--------------------------------------------------------
gpsghv <- glm (GpSGHV~Species, data = fig1)
summary(gpsghv)

##
## Call:
## glm(formula = GpSGHV ~ Species, data = fig1)
##
## Deviance Residuals:
## Min 1Q Median 3Q Max
## -2.0168 -0.7611 -0.3632 0.2073 7.6190
##
## Coefficients:
## Estimate Std. Error t value Pr(>|t|)
## (Intercept) 3.9713 0.6062 6.551 1.53e-09 ***
## SpeciesG. m. morsitans -0.2697 0.6288 -0.429 0.669
## SpeciesG. medicorum -0.8377 1.2859 -0.651 0.516
## SpeciesG. austeni -0.4708 0.6960 -0.676 0.500
## ---
## Signif. codes: 0 '***' 0.001 '**' 0.01 '*' 0.05 '.' 0.1 ' ' 1
##
## (Dispersion parameter for gaussian family taken to be 2.572183)
##
## Null deviance: 308.02 on 122 degrees of freedom
## Residual deviance: 306.09 on 119 degrees of freedom
## AIC: 471.2
##
## Number of Fisher Scoring iterations: 2

fig1$Species=as.factor(fig1$Species)
fig1$Species <- relevel(fig1$Species, ref = "G. austeni")
gpsghv <- glm (GpSGHV~Species, data = fig1)
summary(gpsghv)

##
## Call:
## glm(formula = GpSGHV ~ Species, data = fig1)
##
## Deviance Residuals:
## Min 1Q Median 3Q Max
## -2.0168 -0.7611 -0.3632 0.2073 7.6190
##
## Coefficients:
## Estimate Std. Error t value Pr(>|t|)
## (Intercept) 3.5005 0.3419 10.237 <2e-16 ***
## SpeciesG. pallidipes 0.4708 0.6960 0.676 0.500
## SpeciesG. m. morsitans 0.2011 0.3806 0.528 0.598
## SpeciesG. medicorum -0.3670 1.1845 -0.310 0.757
## ---
## Signif. codes: 0 '***' 0.001 '**' 0.01 '*' 0.05 '.' 0.1 ' ' 1
##
## (Dispersion parameter for gaussian family taken to be 2.572183)
##
## Null deviance: 308.02 on 122 degrees of freedom
## Residual deviance: 306.09 on 119 degrees of freedom
## AIC: 471.2
##
## Number of Fisher Scoring iterations: 2

fig1$Species <- relevel(fig1$Species, ref = "G. medicorum")
gpsghv <- glm (Wigglesworthia~Species, data = fig1)
summary(gpsghv)

##
## Call:
## glm(formula = Wigglesworthia ~ Species, data = fig1)
##
## Deviance Residuals:
## Min 1Q Median 3Q Max
## -2.5887 -1.5709 -0.1274 1.4151 2.5780
##
## Coefficients:
## Estimate Std. Error t value Pr(>|t|)
## (Intercept) 4.708 1.614 2.917 0.00463 **
## SpeciesG. austeni 1.206 1.675 0.720 0.47366
## SpeciesG. pallidipes 2.862 1.743 1.641 0.10478
## SpeciesG. m. morsitans 3.220 1.627 1.978 0.05146 .
## ---
## Signif. codes: 0 '***' 0.001 '**' 0.01 '*' 0.05 '.' 0.1 ' ' 1
##
## (Dispersion parameter for gaussian family taken to be 2.605508)
##
## Null deviance: 252.17 on 80 degrees of freedom
## Residual deviance: 200.62 on 77 degrees of freedom
## (42 observations deleted due to missingness)
## AIC: 313.33
##
## Number of Fisher Scoring iterations: 2

fig1$Species <- relevel(fig1$Species, ref = "G. m. morsitans")
gpsghv <- glm (GpSGHV~Species, data = fig1)
summary(gpsghv)

##
## Call:
## glm(formula = GpSGHV ~ Species, data = fig1)
##
## Deviance Residuals:
## Min 1Q Median 3Q Max
## -2.0168 -0.7611 -0.3632 0.2073 7.6190
##
## Coefficients:
## Estimate Std. Error t value Pr(>|t|)
## (Intercept) 3.7016 0.1672 22.138 <2e-16 ***
## SpeciesG. medicorum -0.5680 1.1463 -0.496 0.621
## SpeciesG. austeni -0.2011 0.3806 -0.528 0.598
## SpeciesG. pallidipes 0.2697 0.6288 0.429 0.669
## ---
## Signif. codes: 0 '***' 0.001 '**' 0.01 '*' 0.05 '.' 0.1 ' ' 1
##
## (Dispersion parameter for gaussian family taken to be 2.572183)
##
## Null deviance: 308.02 on 122 degrees of freedom
## Residual deviance: 306.09 on 119 degrees of freedom
## AIC: 471.2
##
## Number of Fisher Scoring iterations: 2

fig1$Species <- relevel(fig1$Species, ref = "G. pallidipes")
gpsghv <- glm (GpSGHV~Species, data = fig1)
summary(gpsghv)

##
## Call:
## glm(formula = GpSGHV ~ Species, data = fig1)
##
## Deviance Residuals:
## Min 1Q Median 3Q Max
## -2.0168 -0.7611 -0.3632 0.2073 7.6190
##
## Coefficients:
## Estimate Std. Error t value Pr(>|t|)
## (Intercept) 3.9713 0.6062 6.551 1.53e-09 ***
## SpeciesG. m. morsitans -0.2697 0.6288 -0.429 0.669
## SpeciesG. medicorum -0.8377 1.2859 -0.651 0.516
## SpeciesG. austeni -0.4708 0.6960 -0.676 0.500
## ---
## Signif. codes: 0 '***' 0.001 '**' 0.01 '*' 0.05 '.' 0.1 ' ' 1
##
## (Dispersion parameter for gaussian family taken to be 2.572183)
##
## Null deviance: 308.02 on 122 degrees of freedom
## Residual deviance: 306.09 on 119 degrees of freedom
## AIC: 471.2
##
## Number of Fisher Scoring iterations: 2

#-----------------------------------------------
wigg <- glm (Wigglesworthia~Species, data = fig1)
summary(wigg)

##
## Call:
## glm(formula = Wigglesworthia ~ Species, data = fig1)
##
## Deviance Residuals:
## Min 1Q Median 3Q Max
## -2.5887 -1.5709 -0.1274 1.4151 2.5780
##
## Coefficients:
## Estimate Std. Error t value Pr(>|t|)
## (Intercept) 7.5702 0.6590 11.488 <2e-16 ***
## SpeciesG. m. morsitans 0.3576 0.6906 0.518 0.606
## SpeciesG. medicorum -2.8619 1.7435 -1.641 0.105
## SpeciesG. austeni -1.6557 0.7967 -2.078 0.041 *
## ---
## Signif. codes: 0 '***' 0.001 '**' 0.01 '*' 0.05 '.' 0.1 ' ' 1
##
## (Dispersion parameter for gaussian family taken to be 2.605508)
##
## Null deviance: 252.17 on 80 degrees of freedom
## Residual deviance: 200.62 on 77 degrees of freedom
## (42 observations deleted due to missingness)
## AIC: 313.33
##
## Number of Fisher Scoring iterations: 2

fig1$Species=as.factor(fig1$Species)
fig1$Species <- relevel(fig1$Species, ref = "G. austeni")
wigg <- glm (Wigglesworthia~Species, data = fig1)
summary(wigg)

##
## Call:
## glm(formula = Wigglesworthia ~ Species, data = fig1)
##
## Deviance Residuals:
## Min 1Q Median 3Q Max
## -2.5887 -1.5709 -0.1274 1.4151 2.5780
##
## Coefficients:
## Estimate Std. Error t value Pr(>|t|)
## (Intercept) 5.9145 0.4477 13.211 < 2e-16 ***
## SpeciesG. pallidipes 1.6557 0.7967 2.078 0.041014 *
## SpeciesG. m. morsitans 2.0134 0.4931 4.083 0.000108 ***
## SpeciesG. medicorum -1.2062 1.6751 -0.720 0.473659
## ---
## Signif. codes: 0 '***' 0.001 '**' 0.01 '*' 0.05 '.' 0.1 ' ' 1
##
## (Dispersion parameter for gaussian family taken to be 2.605508)
##
## Null deviance: 252.17 on 80 degrees of freedom
## Residual deviance: 200.62 on 77 degrees of freedom
## (42 observations deleted due to missingness)
## AIC: 313.33
##
## Number of Fisher Scoring iterations: 2

fig1$Species <- relevel(fig1$Species, ref = "G. medicorum")
wigg <- glm (Wigglesworthia~Species, data = fig1)
summary(wigg)

##
## Call:
## glm(formula = Wigglesworthia ~ Species, data = fig1)
##
## Deviance Residuals:
## Min 1Q Median 3Q Max
## -2.5887 -1.5709 -0.1274 1.4151 2.5780
##
## Coefficients:
## Estimate Std. Error t value Pr(>|t|)
## (Intercept) 4.708 1.614 2.917 0.00463 **
## SpeciesG. austeni 1.206 1.675 0.720 0.47366
## SpeciesG. pallidipes 2.862 1.743 1.641 0.10478
## SpeciesG. m. morsitans 3.220 1.627 1.978 0.05146 .
## ---
## Signif. codes: 0 '***' 0.001 '**' 0.01 '*' 0.05 '.' 0.1 ' ' 1
##
## (Dispersion parameter for gaussian family taken to be 2.605508)
##
## Null deviance: 252.17 on 80 degrees of freedom
## Residual deviance: 200.62 on 77 degrees of freedom
## (42 observations deleted due to missingness)
## AIC: 313.33
##
## Number of Fisher Scoring iterations: 2

fig1$Species <- relevel(fig1$Species, ref = "G. m. morsitans")
wigg <- glm (Wigglesworthia~Species, data = fig1)
summary(wigg)

##
## Call:
## glm(formula = Wigglesworthia ~ Species, data = fig1)
##
## Deviance Residuals:
## Min 1Q Median 3Q Max
## -2.5887 -1.5709 -0.1274 1.4151 2.5780
##
## Coefficients:
## Estimate Std. Error t value Pr(>|t|)
## (Intercept) 7.9279 0.2067 38.360 < 2e-16 ***
## SpeciesG. medicorum -3.2195 1.6273 -1.978 0.051459 .
## SpeciesG. austeni -2.0134 0.4931 -4.083 0.000108 ***
## SpeciesG. pallidipes -0.3576 0.6906 -0.518 0.606042
## ---
## Signif. codes: 0 '***' 0.001 '**' 0.01 '*' 0.05 '.' 0.1 ' ' 1
##
## (Dispersion parameter for gaussian family taken to be 2.605508)
##
## Null deviance: 252.17 on 80 degrees of freedom
## Residual deviance: 200.62 on 77 degrees of freedom
## (42 observations deleted due to missingness)
## AIC: 313.33
##
## Number of Fisher Scoring iterations: 2

fig1$Species <- relevel(fig1$Species, ref = "G. pallidipes")
wigg <- glm (Wigglesworthia~Species, data = fig1)
summary(wigg)

##
## Call:
## glm(formula = Wigglesworthia ~ Species, data = fig1)
##
## Deviance Residuals:
## Min 1Q Median 3Q Max
## -2.5887 -1.5709 -0.1274 1.4151 2.5780
##
## Coefficients:
## Estimate Std. Error t value Pr(>|t|)
## (Intercept) 7.5702 0.6590 11.488 <2e-16 ***
## SpeciesG. m. morsitans 0.3576 0.6906 0.518 0.606
## SpeciesG. medicorum -2.8619 1.7435 -1.641 0.105
## SpeciesG. austeni -1.6557 0.7967 -2.078 0.041 *
## ---
## Signif. codes: 0 '***' 0.001 '**' 0.01 '*' 0.05 '.' 0.1 ' ' 1
##
## (Dispersion parameter for gaussian family taken to be 2.605508)
##
## Null deviance: 252.17 on 80 degrees of freedom
## Residual deviance: 200.62 on 77 degrees of freedom
## (42 observations deleted due to missingness)
## AIC: 313.33
##
## Number of Fisher Scoring iterations: 2

#----------------------------------------------------
fig1$Species=as.factor(fig1$Species)
fig1$Species <- relevel(fig1$Species, ref = "G. austeni")
sod <- glm (Sodalis~Species, data = fig1)
summary(sod)

##
## Call:
## glm(formula = Sodalis ~ Species, data = fig1)
##
## Deviance Residuals:
## Min 1Q Median 3Q Max
## -2.03030 -0.94315 -0.03394 0.92847 2.61290
##
## Coefficients:
## Estimate Std. Error t value Pr(>|t|)
## (Intercept) 4.1764 0.2466 16.938 < 2e-16 ***
## SpeciesG. pallidipes 1.2313 0.4931 2.497 0.013907 *
## SpeciesG. m. morsitans 1.0943 0.2733 4.004 0.000109 ***
## SpeciesG. medicorum -0.8866 0.8361 -1.060 0.291159
## ---
## Signif. codes: 0 '***' 0.001 '**' 0.01 '*' 0.05 '.' 0.1 ' ' 1
##
## (Dispersion parameter for gaussian family taken to be 1.276671)
##
## Null deviance: 178.24 on 121 degrees of freedom
## Residual deviance: 150.65 on 118 degrees of freedom
## (1 observation deleted due to missingness)
## AIC: 381.95
##
## Number of Fisher Scoring iterations: 2

fig1$Species <- relevel(fig1$Species, ref = "G. medicorum")
sod <- glm (Sodalis~Species, data = fig1)
summary(sod)

##
## Call:
## glm(formula = Sodalis ~ Species, data = fig1)
##
## Deviance Residuals:
## Min 1Q Median 3Q Max
## -2.03030 -0.94315 -0.03394 0.92847 2.61290
##
## Coefficients:
## Estimate Std. Error t value Pr(>|t|)
## (Intercept) 3.2898 0.7990 4.118 7.13e-05 ***
## SpeciesG. austeni 0.8866 0.8361 1.060 0.2912
## SpeciesG. pallidipes 2.1179 0.9059 2.338 0.0211 *
## SpeciesG. m. morsitans 1.9808 0.8076 2.453 0.0156 *
## ---
## Signif. codes: 0 '***' 0.001 '**' 0.01 '*' 0.05 '.' 0.1 ' ' 1
##
## (Dispersion parameter for gaussian family taken to be 1.276671)
##
## Null deviance: 178.24 on 121 degrees of freedom
## Residual deviance: 150.65 on 118 degrees of freedom
## (1 observation deleted due to missingness)
## AIC: 381.95
##
## Number of Fisher Scoring iterations: 2

fig1$Species <- relevel(fig1$Species, ref = "G. m. morsitans")
sod <- glm (Sodalis~Species, data = fig1)
summary(sod)

##
## Call:
## glm(formula = Sodalis ~ Species, data = fig1)
##
## Deviance Residuals:
## Min 1Q Median 3Q Max
## -2.03030 -0.94315 -0.03394 0.92847 2.61290
##
## Coefficients:
## Estimate Std. Error t value Pr(>|t|)
## (Intercept) 5.2707 0.1178 44.743 < 2e-16 ***
## SpeciesG. medicorum -1.9808 0.8076 -2.453 0.015639 *
## SpeciesG. austeni -1.0943 0.2733 -4.004 0.000109 ***
## SpeciesG. pallidipes 0.1370 0.4430 0.309 0.757597
## ---
## Signif. codes: 0 '***' 0.001 '**' 0.01 '*' 0.05 '.' 0.1 ' ' 1
##
## (Dispersion parameter for gaussian family taken to be 1.276671)
##
## Null deviance: 178.24 on 121 degrees of freedom
## Residual deviance: 150.65 on 118 degrees of freedom
## (1 observation deleted due to missingness)
## AIC: 381.95
##
## Number of Fisher Scoring iterations: 2

fig1$Species <- relevel(fig1$Species, ref = "G. pallidipes")
sod <- glm (Sodalis~Species, data = fig1)
summary(sod)

##
## Call:
## glm(formula = Sodalis ~ Species, data = fig1)
##
## Deviance Residuals:
## Min 1Q Median 3Q Max
## -2.03030 -0.94315 -0.03394 0.92847 2.61290
##
## Coefficients:
## Estimate Std. Error t value Pr(>|t|)
## (Intercept) 5.4077 0.4271 12.663 <2e-16 ***
## SpeciesG. m. morsitans -0.1370 0.4430 -0.309 0.7576
## SpeciesG. medicorum -2.1179 0.9059 -2.338 0.0211 *
## SpeciesG. austeni -1.2313 0.4931 -2.497 0.0139 *
## ---
## Signif. codes: 0 '***' 0.001 '**' 0.01 '*' 0.05 '.' 0.1 ' ' 1
##
## (Dispersion parameter for gaussian family taken to be 1.276671)
##
## Null deviance: 178.24 on 121 degrees of freedom
## Residual deviance: 150.65 on 118 degrees of freedom
## (1 observation deleted due to missingness)
## AIC: 381.95
##
## Number of Fisher Scoring iterations: 2

## Preparing supplementary figure 2 and its statistics

fig1 <- read.csv("virus_symb_all_20210613_all3.csv")
head(fig1)

## Country Species Samples GpSGHV Wolbachia Sodalis Wigglesworthia
## 1 South Africa G. austeni W+/V+ 3.071882 5.291369 5.226169 6.837400
## 2 South Africa G. austeni W+/V+ 3.631444 5.563006 2.453318 5.479647
## 3 South Africa G. austeni W+/V+ 3.070407 4.121888 3.376584 5.528074
## 4 South Africa G. austeni W+/V+ 3.071882 5.291369 5.138430 7.157453
## 5 South Africa G. austeni W+/V- 6.330414 6.542078 5.056997 6.218625
## 6 South Africa G. austeni W+/V- 6.322219 6.544068 5.070590 6.208117

summary(fig1)

## Country Species Samples GpSGHV
## Length:123 Length:123 Length:123 Min. : 1.685
## Class :character Class :character Class :character 1st Qu.: 2.917
## Mode :character Mode :character Mode :character Median : 3.313
## Mean : 3.672
## 3rd Qu.: 3.869
## Max. :11.321
##
## Wolbachia Sodalis Wigglesworthia
## Min. : 3.202 Min. :2.453 Min. : 4.708
## 1st Qu.: 6.412 1st Qu.:4.042 1st Qu.: 6.096
## Median : 7.360 Median :5.066 Median : 6.837
## Mean : 7.493 Mean :5.058 Mean : 7.538
## 3rd Qu.: 8.312 3rd Qu.:6.188 3rd Qu.: 9.343
## Max. :15.659 Max. :7.576 Max. :10.506
## NA's :30 NA's :1 NA's :42

figs2a<-ggplot(fig1,aes(x=Samples,y=Wolbachia, fill=Samples)) +
 geom_boxplot() + geom_jitter(width=0.1,alpha=0.2)

tiff("figs2a.tiff", width = 4, height = 4, units = 'in', res = 300)
plot(figs2a+theme_tufte() + theme(axis.line = element_line(size = 1, colour = "black")) + theme(legend.title = element_text(face = "bold")) + theme(legend.text = element_text(face = "italic"))) + theme(axis.text.x = element_blank()) + xlab(expression(bold("Infection Type"))) + ylab(expression (paste (bold("log10 "), bolditalic("Wolbachia"), bold(" copy number"))))

## Warning: Removed 30 rows containing non-finite values (stat_boxplot).

## Warning: Removed 30 rows containing missing values (geom_point).

## Warning: Removed 30 rows containing non-finite values (stat_boxplot).

## Warning: Removed 30 rows containing missing values (geom_point).

dev.off()

## png
## 2

#------------------------------------------------------------------

figs2b<-ggplot(fig1,aes(x=Samples,y=Sodalis, fill=Samples)) +
 geom_boxplot() + geom_jitter(width=0.1,alpha=0.2)

tiff("figs2b.tiff", width = 4, height = 4, units = 'in', res = 300)
plot(figs2b+theme_tufte() + theme(axis.line = element_line(size = 1, colour = "black")) + theme(legend.title = element_text(face = "bold")) + theme(legend.text = element_text(face = "italic"))) + theme(axis.text.x = element_blank()) + xlab(expression(bold("Infection Type"))) + ylab(expression (paste (bold("log10 "), bolditalic("Sodalis"), bold(" copy number"))))

## Warning: Removed 1 rows containing non-finite values (stat_boxplot).

## Warning: Removed 1 rows containing missing values (geom_point).

## Warning: Removed 1 rows containing non-finite values (stat_boxplot).

## Warning: Removed 1 rows containing missing values (geom_point).

dev.off()

## png
## 2

#------------------------------------------------------------------

figs2c<-ggplot(fig1,aes(x=Samples,y=Wigglesworthia, fill=Samples)) +
 geom_boxplot() + geom_jitter(width=0.1,alpha=0.2)

tiff("figs2c.tiff", width = 4, height = 4, units = 'in', res = 300)
plot(figs2c+theme_tufte() + theme(axis.line = element_line(size = 1, colour = "black")) + theme(legend.title = element_text(face = "bold")) + theme(legend.text = element_text(face = "italic"))) + theme(axis.text.x = element_blank()) + xlab(expression(bold("Infection Type"))) + ylab(expression (paste (bold("log10 "), bolditalic("Wigglesworthia"), bold(" copy number"))))

## Warning: Removed 42 rows containing non-finite values (stat_boxplot).

## Warning: Removed 42 rows containing missing values (geom_point).

## Warning: Removed 42 rows containing non-finite values (stat_boxplot).

## Warning: Removed 42 rows containing missing values (geom_point).

dev.off()

## png
## 2

#------------------------------------------------------------------

figs2d<-ggplot(fig1,aes(x=Samples,y=GpSGHV, fill=Samples)) +
 geom_boxplot() + geom_jitter(width=0.1,alpha=0.2)

tiff("figs2d.tiff", width = 4, height = 4, units = 'in', res = 300)
plot(figs2d+theme_tufte() + theme(axis.line = element_line(size = 1, colour = "black")) + theme(legend.title = element_text(face = "bold")) + theme(legend.text = element_text(face = "italic"))) + theme(axis.text.x = element_blank()) + xlab(expression(bold("Infection Type"))) + ylab(expression (bold("log10 GpSGHV copy number ")))
dev.off()

## png
## 2

#--------------------------------------------------------------
#Statistics for supplementary figure 2

fig1

## Country Species Samples GpSGHV Wolbachia Sodalis
## 1 South Africa G. austeni W+/V+ 3.071882 5.291369 5.226169
## 2 South Africa G. austeni W+/V+ 3.631444 5.563006 2.453318
## 3 South Africa G. austeni W+/V+ 3.070407 4.121888 3.376584
## 4 South Africa G. austeni W+/V+ 3.071882 5.291369 5.138430
## 5 South Africa G. austeni W+/V- 6.330414 6.542078 5.056997
## 6 South Africa G. austeni W+/V- 6.322219 6.544068 5.070590
## 7 South Africa G. austeni W+/V- 3.070407 7.722634 4.074324
## 8 South Africa G. austeni W+/V+ 3.071882 5.291369 5.240495
## 9 South Africa G. austeni W+/V- 3.082067 7.797960 4.176986
## 10 South Africa G. austeni W+/V- 6.340841 6.412293 5.103382
## 11 South Africa G. austeni W+/V- 2.606381 6.670246 3.527706
## 12 South Africa G. austeni W+/V+ 3.467164 7.808886 3.083206
## 13 South Africa G. austeni W+/V+ 3.098990 3.201943 3.387768
## 14 Tanzania G. austeni W+/V+ 3.735599 7.030195 3.340603
## 15 South Africa G. austeni W+/V+ 3.850033 6.418301 2.990989
## 16 South Africa G. austeni W+/V+ 3.152288 4.077731 3.357279
## 17 South Africa G. austeni W+/V- 1.905256 7.064458 3.804619
## 18 Tanzania G. austeni W+/V- 3.008600 12.956168 3.314423
## 19 South Africa G. austeni W+/V+ 2.775246 3.959995 NA
## 20 South Africa G. austeni W+/V+ 2.857332 6.989450 6.789324
## 21 South Africa G. austeni W+/V+ 2.716003 NA 6.623851
## 22 South Africa G. austeni W+/V+ 2.775246 NA 2.567822
## 23 Burkina Faso G. medicorum W-/V+ 3.353339 NA 3.501522
## 24 Burkina Faso G. medicorum W-/V+ 2.913814 NA 3.078152
## 25 Zambia G. m. morsitans W+/V- 3.695482 11.193125 6.979752
## 26 Zimbabwe G. m. morsitans W+/V- 4.331225 6.991226 4.705386
## 27 Zimbabwe G. m. morsitans W+/V+ 3.430720 4.483445 5.116050
## 28 Zimbabwe G. m. morsitans W+/V+ 2.938520 6.639486 4.256616
## 29 Zimbabwe G. m. morsitans W+/V+ 3.393751 8.936514 6.961294
## 30 Zimbabwe G. m. morsitans W+/V- 2.878522 7.510009 7.576463
## 31 Zimbabwe G. m. morsitans W+/V- 4.264818 3.889862 6.201792
## 32 Tanzania G. m. morsitans W+/V- 6.561578 8.147367 5.716086
## 33 Zambia G. m. morsitans W+/V+ 3.001734 8.466571 5.635447
## 34 Zimbabwe G. m. morsitans W+/V+ 3.385964 7.399328 5.896440
## 35 Zimbabwe G. m. morsitans W+/V- 3.186391 7.230449 3.827807
## 36 Zimbabwe G. m. morsitans W+/V+ 2.719331 7.360215 3.893365
## 37 Zimbabwe G. m. morsitans W+/V- 3.108565 7.845098 4.668694
## 38 Zimbabwe G. m. morsitans W-/V+ 3.748188 4.705864 6.200526
## 39 Zimbabwe G. m. morsitans W+/V+ 11.320562 4.071882 6.256775
## 40 Zimbabwe G. m. morsitans W+/V+ 3.880814 6.806180 6.414824
## 41 Zimbabwe G. m. morsitans W+/V+ 3.880814 6.806180 6.518537
## 42 Zimbabwe G. m. morsitans W+/V+ 3.393751 8.936514 6.929733
## 43 Zimbabwe G. m. morsitans W+/V- 6.001734 3.985875 4.535826
## 44 Zimbabwe G. m. morsitans W+/V+ 11.320562 4.071882 6.342526
## 45 Tanzania G. m. morsitans W+/V- 3.356408 8.372175 5.854843
## 46 Tanzania G. m. morsitans W+/V- 3.304491 8.268578 6.038572
## 47 Tanzania G. m. morsitans W+/V- 6.606381 8.058426 5.534126
## 48 Tanzania G. m. morsitans W+/V- 3.101747 7.673942 5.896745
## 49 Tanzania G. m. morsitans W+/V- 2.920123 7.265761 6.077336
## 50 Tanzania G. m. morsitans W+/V- 2.982271 7.662758 6.199656
## 51 Tanzania G. m. morsitans W+/V- 2.989450 7.651278 6.258655
## 52 Tanzania G. m. morsitans W+/V- 3.036629 8.409595 5.262641
## 53 Tanzania G. m. morsitans W+/V- 6.619093 8.227372 5.545066
## 54 Tanzania G. m. morsitans W+/V- 3.134814 7.643453 5.961087
## 55 Tanzania G. m. morsitans W+/V- 3.338456 8.335257 6.065603
## 56 Tanzania G. m. morsitans W+/V- 2.898725 7.247482 6.140878
## 57 Tanzania G. m. morsitans W+/V- 3.313023 8.423574 5.268142
## 58 Tanzania G. m. morsitans W+/V- 3.238548 8.416973 5.411659
## 59 Tanzania G. m. morsitans W+/V- 2.944483 7.274158 6.183001
## 60 Tanzania G. m. morsitans W+/V- 2.942504 7.635484 6.238228
## 61 Zambia G. m. morsitans W+/V- 2.517987 14.878522 6.338950
## 62 Zambia G. m. morsitans W+/V- 2.517987 14.878522 6.445490
## 63 Zambia G. m. morsitans W+/V- 2.343999 14.146128 6.789324
## 64 Zambia G. m. morsitans W+/V- 1.989450 15.534534 5.620234
## 65 Zambia G. m. morsitans W+/V- 2.517987 14.878522 6.340893
## 66 Zambia G. m. morsitans W+/V- 1.954243 8.864511 3.496597
## 67 Zimbabwe G. m. morsitans W+/V+ 3.543571 3.989450 5.210836
## 68 Zimbabwe G. m. morsitans W+/V- 4.406199 7.038223 4.700089
## 69 Zimbabwe G. m. morsitans W+/V+ 3.393751 8.936514 6.948944
## 70 Zimbabwe G. m. morsitans W+/V- 3.189771 9.643453 4.017676
## 71 Zimbabwe G. m. morsitans W+/V+ 3.055378 6.523746 5.061756
## 72 Zimbabwe G. m. morsitans W-/V+ 3.729165 4.501333 6.159304
## 73 Zimbabwe G. m. morsitans W+/V- 3.982271 6.248464 3.628272
## 74 Zimbabwe G. m. morsitans W+/V- 2.896526 7.261976 4.395513
## 75 Zimbabwe G. m. morsitans W+/V+ 3.055378 6.523746 4.963742
## 76 Zambia G. m. morsitans W+/V+ 2.496376 8.312177 3.718736
## 77 Zambia G. m. morsitans W+/V+ 2.496376 8.312177 4.014393
## 78 Zambia G. m. morsitans W+/V+ 2.496376 8.312177 4.016192
## 79 Zimbabwe G. m. morsitans W+/V+ 3.845098 7.709270 4.017656
## 80 Zimbabwe G. m. morsitans W+/V+ 2.719331 7.360215 4.150421
## 81 Zimbabwe G. m. morsitans W+/V+ 3.845098 7.709270 4.158713
## 82 Zimbabwe G. m. morsitans W+/V- 2.857332 6.900913 4.304863
## 83 Zimbabwe G. m. morsitans W+/V+ 4.112605 4.377306 3.264307
## 84 Zimbabwe G. m. morsitans W+/V+ 4.112605 4.377306 3.372475
## 85 Zimbabwe G. m. morsitans W+/V+ 4.112605 4.377306 3.446208
## 86 Zimbabwe G. m. morsitans W+/V+ 2.938520 6.639486 4.130066
## 87 Zimbabwe G. m. morsitans W+/V- 3.031812 9.655138 4.228599
## 88 Zimbabwe G. m. morsitans W+/V- 4.176091 4.449633 4.999776
## 89 Zimbabwe G. m. morsitans W+/V- 3.192010 7.803457 4.300718
## 90 Zimbabwe G. m. morsitans W+/V+ 3.845098 7.709270 4.133931
## 91 Zambia G. m. morsitans W+/V- 2.124504 15.658965 5.505514
## 92 Zambia G. m. morsitans W+/V- 2.121888 8.158362 6.220152
## 93 Zambia G. m. morsitans W+/V- 1.684845 7.835056 6.172877
## 94 Zambia G. m. morsitans W+/V- 1.934498 8.134814 6.348968
## 95 Zambia G. m. morsitans W+/V- 1.974972 8.905256 3.378617
## 96 Zimbabwe G. m. morsitans W+/V+ 7.571476 NA 3.388551
## 97 Zimbabwe G. m. morsitans W+/V+ 3.857332 NA 6.626950
## 98 Zimbabwe G. m. morsitans W+/V+ 3.857332 NA 6.676298
## 99 Zimbabwe G. m. morsitans W+/V+ 11.320562 NA 6.333494
## 100 Zimbabwe G. m. morsitans W+/V+ 2.775246 NA 6.894753
## 101 Zimbabwe G. m. morsitans W+/V+ 2.775246 NA 6.904206
## 102 Zimbabwe G. m. morsitans W+/V+ 2.775246 NA 6.919518
## 103 Zimbabwe G. m. morsitans W+/V- 3.453318 NA 4.031085
## 104 Zimbabwe G. m. morsitans W+/V- 3.372912 NA 4.429025
## 105 Zimbabwe G. m. morsitans W+/V- 3.460597 NA 4.482710
## 106 Zimbabwe G. m. morsitans W+/V- 3.946452 NA 4.786975
## 107 Zimbabwe G. m. morsitans W+/V- 4.052309 NA 4.916273
## 108 Zimbabwe G. m. morsitans W+/V- 4.401401 NA 4.712715
## 109 Zimbabwe G. m. morsitans W+/V- 4.139879 NA 5.090553
## 110 Zimbabwe G. m. morsitans W+/V- 3.896526 NA 4.893740
## 111 Zimbabwe G. m. morsitans W+/V- 4.465383 NA 4.822142
## 112 Zimbabwe G. m. morsitans W+/V- 3.824776 NA 4.938539
## 113 Zimbabwe G. m. morsitans W+/V- 3.631444 NA 3.987842
## 114 Zimbabwe G. m. morsitans W+/V- 3.681241 NA 4.183068
## 115 Zimbabwe G. m. morsitans W+/V- 2.792392 NA 4.449532
## 116 Zimbabwe G. m. morsitans W+/V- 4.115278 NA 4.863471
## 117 Tanzania G. pallidipes W+/V+ 3.725912 5.745075 6.190086
## 118 Tanzania G. pallidipes W+/V+ 3.725912 5.745075 6.211020
## 119 Tanzania G. pallidipes W-/V+ 4.610660 NA 5.980494
## 120 Tanzania G. pallidipes W-/V+ 4.577032 NA 6.067431
## 121 Tanzania G. pallidipes W-/V+ 4.583879 NA 6.272031
## 122 Tanzania G. pallidipes W-/V+ 3.468347 NA 3.377431
## 123 Zambia G. pallidipes W-/V+ 3.107210 NA 3.755588
## Wigglesworthia
## 1 6.837400
## 2 5.479647
## 3 5.528074
## 4 7.157453
## 5 6.218625
## 6 6.208117
## 7 NA
## 8 NA
## 9 NA
## 10 NA
## 11 5.733434
## 12 5.135913
## 13 5.497730
## 14 5.893921
## 15 6.963434
## 16 5.291318
## 17 NA
## 18 NA
## 19 NA
## 20 NA
## 21 NA
## 22 4.943486
## 23 4.708313
## 24 NA
## 25 6.678722
## 26 5.339160
## 27 6.310350
## 28 6.443300
## 29 6.116602
## 30 6.804350
## 31 10.191603
## 32 5.459354
## 33 5.899101
## 34 5.952105
## 35 6.344745
## 36 5.784220
## 37 7.800503
## 38 10.062864
## 39 9.388388
## 40 6.937702
## 41 6.805077
## 42 6.182330
## 43 9.838294
## 44 9.297119
## 45 7.098055
## 46 7.124997
## 47 NA
## 48 NA
## 49 NA
## 50 NA
## 51 NA
## 52 NA
## 53 NA
## 54 NA
## 55 NA
## 56 NA
## 57 NA
## 58 NA
## 59 NA
## 60 NA
## 61 NA
## 62 NA
## 63 NA
## 64 NA
## 65 NA
## 66 NA
## 67 NA
## 68 NA
## 69 NA
## 70 NA
## 71 NA
## 72 NA
## 73 5.789754
## 74 6.327612
## 75 6.663816
## 76 6.023311
## 77 6.096269
## 78 6.246218
## 79 6.575373
## 80 6.232853
## 81 6.097164
## 82 7.148731
## 83 8.673505
## 84 8.657838
## 85 8.641584
## 86 6.787858
## 87 6.356982
## 88 10.067865
## 89 NA
## 90 NA
## 91 6.393184
## 92 6.094564
## 93 NA
## 94 NA
## 95 NA
## 96 5.400720
## 97 9.051741
## 98 9.070961
## 99 9.454929
## 100 9.808075
## 101 9.869617
## 102 9.788180
## 103 9.291721
## 104 9.342932
## 105 9.329248
## 106 10.085327
## 107 10.505882
## 108 9.987575
## 109 9.996559
## 110 10.113915
## 111 9.959726
## 112 9.871988
## 113 9.995597
## 114 9.999424
## 115 9.864506
## 116 10.077351
## 117 5.537838
## 118 5.731851
## 119 8.500074
## 120 8.375135
## 121 NA
## 122 8.915657
## 123 8.360722

gpsghv <- glm (GpSGHV~Samples, data = fig1)
summary(gpsghv)

##
## Call:
## glm(formula = GpSGHV ~ Samples, data = fig1)
##
## Deviance Residuals:
## Min 1Q Median 3Q Max
## -1.8239 -0.8037 -0.3995 0.1796 7.4539
##
## Coefficients:
## Estimate Std. Error t value Pr(>|t|)
## (Intercept) 3.78796 0.53082 7.136 7.93e-11 ***
## SamplesW+/V- -0.27919 0.56638 -0.493 0.623
## SamplesW+/V+ 0.07866 0.57752 0.136 0.892
## ---
## Signif. codes: 0 '***' 0.001 '**' 0.01 '*' 0.05 '.' 0.1 ' ' 1
##
## (Dispersion parameter for gaussian family taken to be 2.53596)
##
## Null deviance: 308.02 on 122 degrees of freedom
## Residual deviance: 304.32 on 120 degrees of freedom
## AIC: 468.48
##
## Number of Fisher Scoring iterations: 2

Anova(gpsghv)

## Analysis of Deviance Table (Type II tests)
##
## Response: GpSGHV
## LR Chisq Df Pr(>Chisq)
## Samples 1.4625 2 0.4813

gpsghv <- glm (GpSGHV~Country, data = fig1)
summary(gpsghv)

##
## Call:
## glm(formula = GpSGHV ~ Country, data = fig1)
##
## Deviance Residuals:
## Min 1Q Median 3Q Max
## -1.6081 -0.7318 -0.3762 0.0860 7.2631
##
## Coefficients:
## Estimate Std. Error t value Pr(>|t|)
## (Intercept) 3.1336 1.0711 2.925 0.00413 **
## CountrySouth Africa 0.3798 1.1234 0.338 0.73591
## CountryTanzania 0.6554 1.1132 0.589 0.55713
## CountryZambia -0.7232 1.1324 -0.639 0.52427
## CountryZimbabwe 0.9239 1.0891 0.848 0.39800
## ---
## Signif. codes: 0 '***' 0.001 '**' 0.01 '*' 0.05 '.' 0.1 ' ' 1
##
## (Dispersion parameter for gaussian family taken to be 2.294679)
##
## Null deviance: 308.02 on 122 degrees of freedom
## Residual deviance: 270.77 on 118 degrees of freedom
## AIC: 458.12
##
## Number of Fisher Scoring iterations: 2

Anova(gpsghv)

## Analysis of Deviance Table (Type II tests)
##
## Response: GpSGHV
## LR Chisq Df Pr(>Chisq)
## Country 16.234 4 0.002721 **
## ---
## Signif. codes: 0 '***' 0.001 '**' 0.01 '*' 0.05 '.' 0.1 ' ' 1

gpsghv <- glm (GpSGHV~Species, data = fig1)
summary(gpsghv)

##
## Call:
## glm(formula = GpSGHV ~ Species, data = fig1)
##
## Deviance Residuals:
## Min 1Q Median 3Q Max
## -2.0168 -0.7611 -0.3632 0.2073 7.6190
##
## Coefficients:
## Estimate Std. Error t value Pr(>|t|)
## (Intercept) 3.5005 0.3419 10.237 <2e-16 ***
## SpeciesG. m. morsitans 0.2011 0.3806 0.528 0.598
## SpeciesG. medicorum -0.3670 1.1845 -0.310 0.757
## SpeciesG. pallidipes 0.4708 0.6960 0.676 0.500
## ---
## Signif. codes: 0 '***' 0.001 '**' 0.01 '*' 0.05 '.' 0.1 ' ' 1
##
## (Dispersion parameter for gaussian family taken to be 2.572183)
##
## Null deviance: 308.02 on 122 degrees of freedom
## Residual deviance: 306.09 on 119 degrees of freedom
## AIC: 471.2
##
## Number of Fisher Scoring iterations: 2

Anova(gpsghv)

## Analysis of Deviance Table (Type II tests)
##
## Response: GpSGHV
## LR Chisq Df Pr(>Chisq)
## Species 0.75201 3 0.8609

#--------------------------------------------------
wol <- glm (Wolbachia~Samples, data = fig1)
summary(wol)

##
## Call:
## glm(formula = Wolbachia ~ Samples, data = fig1)
##
## Deviance Residuals:
## Min 1Q Median 3Q Max
## -4.6581 -1.3175 -0.4131 1.0681 7.1111
##
## Coefficients:
## Estimate Std. Error t value Pr(>|t|)
## (Intercept) 4.604 1.663 2.769 0.00683 **
## SamplesW+/V- 3.944 1.695 2.327 0.02219 *
## SamplesW+/V+ 1.688 1.704 0.991 0.32428
## ---
## Signif. codes: 0 '***' 0.001 '**' 0.01 '*' 0.05 '.' 0.1 ' ' 1
##
## (Dispersion parameter for gaussian family taken to be 5.527992)
##
## Null deviance: 628.66 on 92 degrees of freedom
## Residual deviance: 497.52 on 90 degrees of freedom
## (30 observations deleted due to missingness)
## AIC: 427.89
##
## Number of Fisher Scoring iterations: 2

Anova(wol)

## Analysis of Deviance Table (Type II tests)
##
## Response: Wolbachia
## LR Chisq Df Pr(>Chisq)
## Samples 23.723 2 7.057e-06 ***
## ---
## Signif. codes: 0 '***' 0.001 '**' 0.01 '*' 0.05 '.' 0.1 ' ' 1

wol <- glm (Wolbachia~Country, data = fig1)
summary(wol)

##
## Call:
## glm(formula = Wolbachia ~ Country, data = fig1)
##
## Deviance Residuals:
## Min 1Q Median 3Q Max
## -3.1943 -1.9716 0.1638 0.8887 5.0424
##
## Coefficients:
## Estimate Std. Error t value Pr(>|t|)
## (Intercept) 5.9316 0.4636 12.795 < 2e-16 ***
## CountryTanzania 1.9822 0.6318 3.138 0.00231 **
## CountryZambia 5.0977 0.6758 7.543 3.72e-11 ***
## CountryZimbabwe 0.5791 0.5628 1.029 0.30630
## ---
## Signif. codes: 0 '***' 0.001 '**' 0.01 '*' 0.05 '.' 0.1 ' ' 1
##
## (Dispersion parameter for gaussian family taken to be 3.86852)
##
## Null deviance: 628.66 on 92 degrees of freedom
## Residual deviance: 344.30 on 89 degrees of freedom
## (30 observations deleted due to missingness)
## AIC: 395.65
##
## Number of Fisher Scoring iterations: 2

Anova(wol)

## Analysis of Deviance Table (Type II tests)
##
## Response: Wolbachia
## LR Chisq Df Pr(>Chisq)
## Country 73.507 3 7.571e-16 ***
## ---
## Signif. codes: 0 '***' 0.001 '**' 0.01 '*' 0.05 '.' 0.1 ' ' 1

wol <- glm (Wolbachia~Species, data = fig1)
summary(wol)

##
## Call:
## glm(formula = Wolbachia ~ Species, data = fig1)
##
## Deviance Residuals:
## Min 1Q Median 3Q Max
## -3.9776 -1.0464 -0.1582 0.5495 7.7915
##
## Coefficients:
## Estimate Std. Error t value Pr(>|t|)
## (Intercept) 6.3378 0.5705 11.109 <2e-16 ***
## SpeciesG. m. morsitans 1.5297 0.6459 2.368 0.020 *
## SpeciesG. pallidipes -0.5927 1.8922 -0.313 0.755
## ---
## Signif. codes: 0 '***' 0.001 '**' 0.01 '*' 0.05 '.' 0.1 ' ' 1
##
## (Dispersion parameter for gaussian family taken to be 6.510038)
##
## Null deviance: 628.66 on 92 degrees of freedom
## Residual deviance: 585.90 on 90 degrees of freedom
## (30 observations deleted due to missingness)
## AIC: 443.09
##
## Number of Fisher Scoring iterations: 2

Anova(wol)

## Analysis of Deviance Table (Type II tests)
##
## Response: Wolbachia
## LR Chisq Df Pr(>Chisq)
## Species 6.5678 2 0.03748 *
## ---
## Signif. codes: 0 '***' 0.001 '**' 0.01 '*' 0.05 '.' 0.1 ' ' 1

#wolgp <- subset(fig1, Species=="G. pallidipes")
#wol <- glm (Wolbachia~Samples, data = wolgp)
#summary(wol)

wolga <- subset(fig1, Species=="G. austeni")
wolga$Samples=as.factor(wolga$Samples)
wolga$Samples <- relevel(wolga$Samples, ref = "W+/V+")
wol <- glm (Wolbachia~Samples, data = wolga)
summary(wol)

##
## Call:
## glm(formula = Wolbachia ~ Samples, data = wolga)
##
## Deviance Residuals:
## Min 1Q Median 3Q Max
## -2.2185 -1.2034 -0.1291 0.3564 5.2424
##
## Coefficients:
## Estimate Std. Error t value Pr(>|t|)
## (Intercept) 5.4205 0.5083 10.664 3.29e-09 ***
## SamplesW+/V- 2.2933 0.8037 2.854 0.0106 *
## ---
## Signif. codes: 0 '***' 0.001 '**' 0.01 '*' 0.05 '.' 0.1 ' ' 1
##
## (Dispersion parameter for gaussian family taken to be 3.100161)
##
## Null deviance: 81.047 on 19 degrees of freedom
## Residual deviance: 55.803 on 18 degrees of freedom
## (2 observations deleted due to missingness)
## AIC: 83.279
##
## Number of Fisher Scoring iterations: 2

wolgmm <- subset(fig1, Species=="G. m. morsitans")
wolgmm$Samples=as.factor(wolgmm$Samples)
wolgmm$Samples <- relevel(wolgmm$Samples, ref = "W+/V+")
wol <- glm (Wolbachia~Samples, data = wolgmm)
summary(wol)

##
## Call:
## glm(formula = Wolbachia ~ Samples, data = wolgmm)
##
## Deviance Residuals:
## Min 1Q Median 3Q Max
## -4.8132 -1.4332 -0.3309 0.9462 6.9559
##
## Coefficients:
## Estimate Std. Error t value Pr(>|t|)
## (Intercept) 6.7364 0.4876 13.817 < 2e-16 ***
## SamplesW-/V+ -2.1328 1.8243 -1.169 0.24642
## SamplesW+/V- 1.9667 0.6176 3.184 0.00219 **
## ---
## Signif. codes: 0 '***' 0.001 '**' 0.01 '*' 0.05 '.' 0.1 ' ' 1
##
## (Dispersion parameter for gaussian family taken to be 6.180364)
##
## Null deviance: 504.86 on 70 degrees of freedom
## Residual deviance: 420.26 on 68 degrees of freedom
## (21 observations deleted due to missingness)
## AIC: 335.74
##
## Number of Fisher Scoring iterations: 2

#wolgp <- subset(fig1, Species=="G. pallidipes")
#wolgp$Samples=as.factor(wolgp$Samples)
#wolgp$Samples <- relevel(wolgp$Samples, ref = "W+/V+")
#wol <- glm (Wolbachia~Samples, data = wolgp)
#summary(wol)

fig1$Samples=as.factor(fig1$Samples)
fig1$Samples <- relevel(fig1$Samples, ref = "W+/V+")
wol <- glm (Wolbachia~Samples, data = fig1)
summary(wol)

##
## Call:
## glm(formula = Wolbachia ~ Samples, data = fig1)
##
## Deviance Residuals:
## Min 1Q Median 3Q Max
## -4.6581 -1.3175 -0.4131 1.0681 7.1111
##
## Coefficients:
## Estimate Std. Error t value Pr(>|t|)
## (Intercept) 6.2921 0.3718 16.925 < 2e-16 ***
## SamplesW-/V+ -1.6885 1.7036 -0.991 0.324
## SamplesW+/V- 2.2558 0.4966 4.543 1.72e-05 ***
## ---
## Signif. codes: 0 '***' 0.001 '**' 0.01 '*' 0.05 '.' 0.1 ' ' 1
##
## (Dispersion parameter for gaussian family taken to be 5.527992)
##
## Null deviance: 628.66 on 92 degrees of freedom
## Residual deviance: 497.52 on 90 degrees of freedom
## (30 observations deleted due to missingness)
## AIC: 427.89
##
## Number of Fisher Scoring iterations: 2

fig1$Samples <- relevel(fig1$Samples, ref = "W+/V-")
wol <- glm (Wolbachia~Samples, data = fig1)
summary(wol)

##
## Call:
## glm(formula = Wolbachia ~ Samples, data = fig1)
##
## Deviance Residuals:
## Min 1Q Median 3Q Max
## -4.6581 -1.3175 -0.4131 1.0681 7.1111
##
## Coefficients:
## Estimate Std. Error t value Pr(>|t|)
## (Intercept) 8.5479 0.3292 25.963 < 2e-16 ***
## SamplesW+/V+ -2.2558 0.4966 -4.543 1.72e-05 ***
## SamplesW-/V+ -3.9443 1.6948 -2.327 0.0222 *
## ---
## Signif. codes: 0 '***' 0.001 '**' 0.01 '*' 0.05 '.' 0.1 ' ' 1
##
## (Dispersion parameter for gaussian family taken to be 5.527992)
##
## Null deviance: 628.66 on 92 degrees of freedom
## Residual deviance: 497.52 on 90 degrees of freedom
## (30 observations deleted due to missingness)
## AIC: 427.89
##
## Number of Fisher Scoring iterations: 2

fig1$Samples <- relevel(fig1$Samples, ref = "W-/V+")
wol <- glm (Wolbachia~Samples, data = fig1)
summary(wol)

##
## Call:
## glm(formula = Wolbachia ~ Samples, data = fig1)
##
## Deviance Residuals:
## Min 1Q Median 3Q Max
## -4.6581 -1.3175 -0.4131 1.0681 7.1111
##
## Coefficients:
## Estimate Std. Error t value Pr(>|t|)
## (Intercept) 4.604 1.663 2.769 0.00683 **
## SamplesW+/V- 3.944 1.695 2.327 0.02219 *
## SamplesW+/V+ 1.688 1.704 0.991 0.32428
## ---
## Signif. codes: 0 '***' 0.001 '**' 0.01 '*' 0.05 '.' 0.1 ' ' 1
##
## (Dispersion parameter for gaussian family taken to be 5.527992)
##
## Null deviance: 628.66 on 92 degrees of freedom
## Residual deviance: 497.52 on 90 degrees of freedom
## (30 observations deleted due to missingness)
## AIC: 427.89
##
## Number of Fisher Scoring iterations: 2

#------------------------------------------------------
wigg <- glm (Wigglesworthia~Samples, data = fig1)
summary(wigg)

##
## Call:
## glm(formula = Wigglesworthia ~ Samples, data = fig1)
##
## Deviance Residuals:
## Min 1Q Median 3Q Max
## -3.4455 -1.3952 -0.3577 1.7405 2.9366
##
## Coefficients:
## Estimate Std. Error t value Pr(>|t|)
## (Intercept) 8.153794 0.688331 11.846 <2e-16 ***
## SamplesW+/V- 0.006218 0.746600 0.008 0.993
## SamplesW+/V+ -1.220761 0.736978 -1.656 0.102
## ---
## Signif. codes: 0 '***' 0.001 '**' 0.01 '*' 0.05 '.' 0.1 ' ' 1
##
## (Dispersion parameter for gaussian family taken to be 2.842799)
##
## Null deviance: 252.17 on 80 degrees of freedom
## Residual deviance: 221.74 on 78 degrees of freedom
## (42 observations deleted due to missingness)
## AIC: 319.44
##
## Number of Fisher Scoring iterations: 2

Anova(wigg)

## Analysis of Deviance Table (Type II tests)
##
## Response: Wigglesworthia
## LR Chisq Df Pr(>Chisq)
## Samples 10.706 2 0.004734 **
## ---
## Signif. codes: 0 '***' 0.001 '**' 0.01 '*' 0.05 '.' 0.1 ' ' 1

fig1$Samples=as.factor(fig1$Samples)
fig1$Samples <- relevel(fig1$Samples, ref = "W+/V+")
wigg <- glm (Wigglesworthia~Samples, data = fig1)
summary(wigg)

##
## Call:
## glm(formula = Wigglesworthia ~ Samples, data = fig1)
##
## Deviance Residuals:
## Min 1Q Median 3Q Max
## -3.4455 -1.3952 -0.3577 1.7405 2.9366
##
## Coefficients:
## Estimate Std. Error t value Pr(>|t|)
## (Intercept) 6.9330 0.2633 26.329 < 2e-16 ***
## SamplesW-/V+ 1.2208 0.7370 1.656 0.10165
## SamplesW+/V- 1.2270 0.3911 3.137 0.00241 **
## ---
## Signif. codes: 0 '***' 0.001 '**' 0.01 '*' 0.05 '.' 0.1 ' ' 1
##
## (Dispersion parameter for gaussian family taken to be 2.842799)
##
## Null deviance: 252.17 on 80 degrees of freedom
## Residual deviance: 221.74 on 78 degrees of freedom
## (42 observations deleted due to missingness)
## AIC: 319.44
##
## Number of Fisher Scoring iterations: 2

fig1$Samples <- relevel(fig1$Samples, ref = "W+/V-")
wigg <- glm (Wigglesworthia~Samples, data = fig1)
summary(wigg)

##
## Call:
## glm(formula = Wigglesworthia ~ Samples, data = fig1)
##
## Deviance Residuals:
## Min 1Q Median 3Q Max
## -3.4455 -1.3952 -0.3577 1.7405 2.9366
##
## Coefficients:
## Estimate Std. Error t value Pr(>|t|)
## (Intercept) 8.160012 0.289157 28.220 < 2e-16 ***
## SamplesW+/V+ -1.226978 0.391086 -3.137 0.00241 **
## SamplesW-/V+ -0.006218 0.746600 -0.008 0.99338
## ---
## Signif. codes: 0 '***' 0.001 '**' 0.01 '*' 0.05 '.' 0.1 ' ' 1
##
## (Dispersion parameter for gaussian family taken to be 2.842799)
##
## Null deviance: 252.17 on 80 degrees of freedom
## Residual deviance: 221.74 on 78 degrees of freedom
## (42 observations deleted due to missingness)
## AIC: 319.44
##
## Number of Fisher Scoring iterations: 2

fig1$Samples <- relevel(fig1$Samples, ref = "W-/V+")
wigg <- glm (Wigglesworthia~Samples, data = fig1)
summary(wigg)

##
## Call:
## glm(formula = Wigglesworthia ~ Samples, data = fig1)
##
## Deviance Residuals:
## Min 1Q Median 3Q Max
## -3.4455 -1.3952 -0.3577 1.7405 2.9366
##
## Coefficients:
## Estimate Std. Error t value Pr(>|t|)
## (Intercept) 8.153794 0.688331 11.846 <2e-16 ***
## SamplesW+/V- 0.006218 0.746600 0.008 0.993
## SamplesW+/V+ -1.220761 0.736978 -1.656 0.102
## ---
## Signif. codes: 0 '***' 0.001 '**' 0.01 '*' 0.05 '.' 0.1 ' ' 1
##
## (Dispersion parameter for gaussian family taken to be 2.842799)
##
## Null deviance: 252.17 on 80 degrees of freedom
## Residual deviance: 221.74 on 78 degrees of freedom
## (42 observations deleted due to missingness)
## AIC: 319.44
##
## Number of Fisher Scoring iterations: 2

wigg <- glm (Wigglesworthia~Country, data = fig1)
summary(wigg)

##
## Call:
## glm(formula = Wigglesworthia ~ Country, data = fig1)
##
## Deviance Residuals:
## Min 1Q Median 3Q Max
## -2.9057 -1.4218 0.1384 1.5404 2.2611
##
## Coefficients:
## Estimate Std. Error t value Pr(>|t|)
## (Intercept) 4.708 1.518 3.102 0.0027 **
## CountrySouth Africa 1.208 1.580 0.765 0.4468
## CountryTanzania 2.251 1.600 1.407 0.1634
## CountryZambia 1.766 1.610 1.097 0.2762
## CountryZimbabwe 3.537 1.533 2.308 0.0237 *
## ---
## Signif. codes: 0 '***' 0.001 '**' 0.01 '*' 0.05 '.' 0.1 ' ' 1
##
## (Dispersion parameter for gaussian family taken to be 2.303399)
##
## Null deviance: 252.17 on 80 degrees of freedom
## Residual deviance: 175.06 on 76 degrees of freedom
## (42 observations deleted due to missingness)
## AIC: 304.29
##
## Number of Fisher Scoring iterations: 2

Anova(wigg)

## Analysis of Deviance Table (Type II tests)
##
## Response: Wigglesworthia
## LR Chisq Df Pr(>Chisq)
## Country 33.479 4 9.53e-07 ***
## ---
## Signif. codes: 0 '***' 0.001 '**' 0.01 '*' 0.05 '.' 0.1 ' ' 1

wigg <- glm (Wigglesworthia~Species, data = fig1)
summary(wigg)

##
## Call:
## glm(formula = Wigglesworthia ~ Species, data = fig1)
##
## Deviance Residuals:
## Min 1Q Median 3Q Max
## -2.5887 -1.5709 -0.1274 1.4151 2.5780
##
## Coefficients:
## Estimate Std. Error t value Pr(>|t|)
## (Intercept) 5.9145 0.4477 13.211 < 2e-16 ***
## SpeciesG. m. morsitans 2.0134 0.4931 4.083 0.000108 ***
## SpeciesG. medicorum -1.2062 1.6751 -0.720 0.473659
## SpeciesG. pallidipes 1.6557 0.7967 2.078 0.041014 *
## ---
## Signif. codes: 0 '***' 0.001 '**' 0.01 '*' 0.05 '.' 0.1 ' ' 1
##
## (Dispersion parameter for gaussian family taken to be 2.605508)
##
## Null deviance: 252.17 on 80 degrees of freedom
## Residual deviance: 200.62 on 77 degrees of freedom
## (42 observations deleted due to missingness)
## AIC: 313.33
##
## Number of Fisher Scoring iterations: 2

Anova(wigg)

## Analysis of Deviance Table (Type II tests)
##
## Response: Wigglesworthia
## LR Chisq Df Pr(>Chisq)
## Species 19.785 3 0.0001881 ***
## ---
## Signif. codes: 0 '***' 0.001 '**' 0.01 '*' 0.05 '.' 0.1 ' ' 1

sod <- glm (Sodalis~Samples, data = fig1)
summary(sod)

##
## Call:
## glm(formula = Sodalis ~ Samples, data = fig1)
##
## Deviance Residuals:
## Min 1Q Median 3Q Max
## -2.5165 -0.9565 -0.0430 1.0756 2.4365
##
## Coefficients:
## Estimate Std. Error t value Pr(>|t|)
## (Intercept) 4.93250 0.40686 12.123 <2e-16 ***
## SamplesW+/V- 0.20747 0.43411 0.478 0.634
## SamplesW+/V+ 0.03731 0.44336 0.084 0.933
## ---
## Signif. codes: 0 '***' 0.001 '**' 0.01 '*' 0.05 '.' 0.1 ' ' 1
##
## (Dispersion parameter for gaussian family taken to be 1.489802)
##
## Null deviance: 178.24 on 121 degrees of freedom
## Residual deviance: 177.29 on 119 degrees of freedom
## (1 observation deleted due to missingness)
## AIC: 399.82
##
## Number of Fisher Scoring iterations: 2

Anova(sod)

## Analysis of Deviance Table (Type II tests)
##
## Response: Sodalis
## LR Chisq Df Pr(>Chisq)
## Samples 0.63888 2 0.7266

sod <- glm (Sodalis~Country, data = fig1)
summary(sod)

##
## Call:
## glm(formula = Sodalis ~ Country, data = fig1)
##
## Deviance Residuals:
## Min 1Q Median 3Q Max
## -2.30181 -0.87642 -0.07664 0.83645 2.52354
##
## Coefficients:
## Estimate Std. Error t value Pr(>|t|)
## (Intercept) 3.2898 0.8031 4.096 7.76e-05 ***
## CountrySouth Africa 0.9759 0.8443 1.156 0.2501
## CountryTanzania 2.3264 0.8346 2.787 0.0062 **
## CountryZambia 2.0500 0.8490 2.415 0.0173 *
## CountryZimbabwe 1.7649 0.8166 2.161 0.0327 *
## ---
## Signif. codes: 0 '***' 0.001 '**' 0.01 '*' 0.05 '.' 0.1 ' ' 1
##
## (Dispersion parameter for gaussian family taken to be 1.289906)
##
## Null deviance: 178.24 on 121 degrees of freedom
## Residual deviance: 150.92 on 117 degrees of freedom
## (1 observation deleted due to missingness)
## AIC: 384.17
##
## Number of Fisher Scoring iterations: 2

Anova(sod)

## Analysis of Deviance Table (Type II tests)
##
## Response: Sodalis
## LR Chisq Df Pr(>Chisq)
## Country 21.179 4 0.0002918 ***
## ---
## Signif. codes: 0 '***' 0.001 '**' 0.01 '*' 0.05 '.' 0.1 ' ' 1

sod <- glm (Sodalis~Species, data = fig1)
summary(sod)

##
## Call:
## glm(formula = Sodalis ~ Species, data = fig1)
##
## Deviance Residuals:
## Min 1Q Median 3Q Max
## -2.03030 -0.94315 -0.03394 0.92847 2.61290
##
## Coefficients:
## Estimate Std. Error t value Pr(>|t|)
## (Intercept) 4.1764 0.2466 16.938 < 2e-16 ***
## SpeciesG. m. morsitans 1.0943 0.2733 4.004 0.000109 ***
## SpeciesG. medicorum -0.8866 0.8361 -1.060 0.291159
## SpeciesG. pallidipes 1.2313 0.4931 2.497 0.013907 *
## ---
## Signif. codes: 0 '***' 0.001 '**' 0.01 '*' 0.05 '.' 0.1 ' ' 1
##
## (Dispersion parameter for gaussian family taken to be 1.276671)
##
## Null deviance: 178.24 on 121 degrees of freedom
## Residual deviance: 150.65 on 118 degrees of freedom
## (1 observation deleted due to missingness)
## AIC: 381.95
##
## Number of Fisher Scoring iterations: 2

Anova(sod)

## Analysis of Deviance Table (Type II tests)
##
## Response: Sodalis
## LR Chisq Df Pr(>Chisq)
## Species 21.612 3 7.856e-05 ***
## ---
## Signif. codes: 0 '***' 0.001 '**' 0.01 '*' 0.05 '.' 0.1 ' ' 1

## Preparing supplementary figure 3 and its statistics

fig1 <- read.csv("virus_symb_all_20210613_all3.csv")
head(fig1)

## Country Species Samples GpSGHV Wolbachia Sodalis Wigglesworthia
## 1 South Africa G. austeni W+/V+ 3.071882 5.291369 5.226169 6.837400
## 2 South Africa G. austeni W+/V+ 3.631444 5.563006 2.453318 5.479647
## 3 South Africa G. austeni W+/V+ 3.070407 4.121888 3.376584 5.528074
## 4 South Africa G. austeni W+/V+ 3.071882 5.291369 5.138430 7.157453
## 5 South Africa G. austeni W+/V- 6.330414 6.542078 5.056997 6.218625
## 6 South Africa G. austeni W+/V- 6.322219 6.544068 5.070590 6.208117

summary(fig1)

## Country Species Samples GpSGHV
## Length:123 Length:123 Length:123 Min. : 1.685
## Class :character Class :character Class :character 1st Qu.: 2.917
## Mode :character Mode :character Mode :character Median : 3.313
## Mean : 3.672
## 3rd Qu.: 3.869
## Max. :11.321
##
## Wolbachia Sodalis Wigglesworthia
## Min. : 3.202 Min. :2.453 Min. : 4.708
## 1st Qu.: 6.412 1st Qu.:4.042 1st Qu.: 6.096
## Median : 7.360 Median :5.066 Median : 6.837
## Mean : 7.493 Mean :5.058 Mean : 7.538
## 3rd Qu.: 8.312 3rd Qu.:6.188 3rd Qu.: 9.343
## Max. :15.659 Max. :7.576 Max. :10.506
## NA's :30 NA's :1 NA's :42

figs3a<-ggplot(fig1,aes(x=Country,y=Wolbachia, fill=Country)) +
 geom_boxplot() + geom_jitter(width=0.1,alpha=0.2)

tiff("figs3a.tiff", width = 4, height = 4, units = 'in', res = 300)
plot(figs3a+theme_tufte() + theme(axis.line = element_line(size = 1, colour = "black")) + theme(legend.title = element_text(face = "bold")) + theme(legend.text = element_text(face = "italic"))) + theme(axis.text.x = element_blank()) + xlab(expression(bold("Countries"))) + ylab(expression (paste (bold("log10 "), bolditalic("Wolbachia"), bold(" copy number"))))

## Warning: Removed 30 rows containing non-finite values (stat_boxplot).

## Warning: Removed 30 rows containing missing values (geom_point).

## Warning: Removed 30 rows containing non-finite values (stat_boxplot).

## Warning: Removed 30 rows containing missing values (geom_point).

dev.off()

## png
## 2

#------------------------------------------------------------------

figs3b<-ggplot(fig1,aes(x=Country,y=Sodalis, fill=Country)) +
 geom_boxplot() + geom_jitter(width=0.1,alpha=0.2)

tiff("figs3b.tiff", width = 4, height = 4, units = 'in', res = 300)
plot(figs3b+theme_tufte() + theme(axis.line = element_line(size = 1, colour = "black")) + theme(legend.title = element_text(face = "bold")) + theme(legend.text = element_text(face = "italic"))) + theme(axis.text.x = element_blank()) + xlab(expression(bold("Countries"))) + ylab(expression (paste (bold("log10 "), bolditalic("Sodalis"), bold(" copy number"))))

## Warning: Removed 1 rows containing non-finite values (stat_boxplot).

## Warning: Removed 1 rows containing missing values (geom_point).

## Warning: Removed 1 rows containing non-finite values (stat_boxplot).

## Warning: Removed 1 rows containing missing values (geom_point).

dev.off()

## png
## 2

#------------------------------------------------------------------

figs3c<-ggplot(fig1,aes(x=Country,y=Wigglesworthia, fill=Country)) +
 geom_boxplot() + geom_jitter(width=0.1,alpha=0.2)

tiff("figs3c.tiff", width = 4, height = 4, units = 'in', res = 300)
plot(figs3c+theme_tufte() + theme(axis.line = element_line(size = 1, colour = "black")) + theme(legend.title = element_text(face = "bold")) + theme(legend.text = element_text(face = "italic"))) + theme(axis.text.x = element_blank()) + xlab(expression(bold("Countries"))) + ylab(expression (paste (bold("log10 "), bolditalic("Wigglesworthia"), bold(" copy number"))))

## Warning: Removed 42 rows containing non-finite values (stat_boxplot).

## Warning: Removed 42 rows containing missing values (geom_point).

## Warning: Removed 42 rows containing non-finite values (stat_boxplot).

## Warning: Removed 42 rows containing missing values (geom_point).

dev.off()

## png
## 2

#------------------------------------------------------------------

figs3d<-ggplot(fig1,aes(x=Country,y=GpSGHV, fill=Country)) +
 geom_boxplot() + geom_jitter(width=0.1,alpha=0.2)

tiff("figs3d.tiff", width = 4, height = 4, units = 'in', res = 300)
plot(figs3d+theme_tufte() + theme(axis.line = element_line(size = 1, colour = "black")) + theme(legend.title = element_text(face = "bold")) + theme(legend.text = element_text(face = "italic"))) + theme(axis.text.x = element_blank()) + xlab(expression(bold("Countries"))) + ylab(expression (bold("log10 GpSGHV copy number ")))
dev.off()

## png
## 2

#------------------------------------------------------------------
#statistics for supplementary figure 3 (countries)

fig1

## Country Species Samples GpSGHV Wolbachia Sodalis
## 1 South Africa G. austeni W+/V+ 3.071882 5.291369 5.226169
## 2 South Africa G. austeni W+/V+ 3.631444 5.563006 2.453318
## 3 South Africa G. austeni W+/V+ 3.070407 4.121888 3.376584
## 4 South Africa G. austeni W+/V+ 3.071882 5.291369 5.138430
## 5 South Africa G. austeni W+/V- 6.330414 6.542078 5.056997
## 6 South Africa G. austeni W+/V- 6.322219 6.544068 5.070590
## 7 South Africa G. austeni W+/V- 3.070407 7.722634 4.074324
## 8 South Africa G. austeni W+/V+ 3.071882 5.291369 5.240495
## 9 South Africa G. austeni W+/V- 3.082067 7.797960 4.176986
## 10 South Africa G. austeni W+/V- 6.340841 6.412293 5.103382
## 11 South Africa G. austeni W+/V- 2.606381 6.670246 3.527706
## 12 South Africa G. austeni W+/V+ 3.467164 7.808886 3.083206
## 13 South Africa G. austeni W+/V+ 3.098990 3.201943 3.387768
## 14 Tanzania G. austeni W+/V+ 3.735599 7.030195 3.340603
## 15 South Africa G. austeni W+/V+ 3.850033 6.418301 2.990989
## 16 South Africa G. austeni W+/V+ 3.152288 4.077731 3.357279
## 17 South Africa G. austeni W+/V- 1.905256 7.064458 3.804619
## 18 Tanzania G. austeni W+/V- 3.008600 12.956168 3.314423
## 19 South Africa G. austeni W+/V+ 2.775246 3.959995 NA
## 20 South Africa G. austeni W+/V+ 2.857332 6.989450 6.789324
## 21 South Africa G. austeni W+/V+ 2.716003 NA 6.623851
## 22 South Africa G. austeni W+/V+ 2.775246 NA 2.567822
## 23 Burkina Faso G. medicorum W-/V+ 3.353339 NA 3.501522
## 24 Burkina Faso G. medicorum W-/V+ 2.913814 NA 3.078152
## 25 Zambia G. m. morsitans W+/V- 3.695482 11.193125 6.979752
## 26 Zimbabwe G. m. morsitans W+/V- 4.331225 6.991226 4.705386
## 27 Zimbabwe G. m. morsitans W+/V+ 3.430720 4.483445 5.116050
## 28 Zimbabwe G. m. morsitans W+/V+ 2.938520 6.639486 4.256616
## 29 Zimbabwe G. m. morsitans W+/V+ 3.393751 8.936514 6.961294
## 30 Zimbabwe G. m. morsitans W+/V- 2.878522 7.510009 7.576463
## 31 Zimbabwe G. m. morsitans W+/V- 4.264818 3.889862 6.201792
## 32 Tanzania G. m. morsitans W+/V- 6.561578 8.147367 5.716086
## 33 Zambia G. m. morsitans W+/V+ 3.001734 8.466571 5.635447
## 34 Zimbabwe G. m. morsitans W+/V+ 3.385964 7.399328 5.896440
## 35 Zimbabwe G. m. morsitans W+/V- 3.186391 7.230449 3.827807
## 36 Zimbabwe G. m. morsitans W+/V+ 2.719331 7.360215 3.893365
## 37 Zimbabwe G. m. morsitans W+/V- 3.108565 7.845098 4.668694
## 38 Zimbabwe G. m. morsitans W-/V+ 3.748188 4.705864 6.200526
## 39 Zimbabwe G. m. morsitans W+/V+ 11.320562 4.071882 6.256775
## 40 Zimbabwe G. m. morsitans W+/V+ 3.880814 6.806180 6.414824
## 41 Zimbabwe G. m. morsitans W+/V+ 3.880814 6.806180 6.518537
## 42 Zimbabwe G. m. morsitans W+/V+ 3.393751 8.936514 6.929733
## 43 Zimbabwe G. m. morsitans W+/V- 6.001734 3.985875 4.535826
## 44 Zimbabwe G. m. morsitans W+/V+ 11.320562 4.071882 6.342526
## 45 Tanzania G. m. morsitans W+/V- 3.356408 8.372175 5.854843
## 46 Tanzania G. m. morsitans W+/V- 3.304491 8.268578 6.038572
## 47 Tanzania G. m. morsitans W+/V- 6.606381 8.058426 5.534126
## 48 Tanzania G. m. morsitans W+/V- 3.101747 7.673942 5.896745
## 49 Tanzania G. m. morsitans W+/V- 2.920123 7.265761 6.077336
## 50 Tanzania G. m. morsitans W+/V- 2.982271 7.662758 6.199656
## 51 Tanzania G. m. morsitans W+/V- 2.989450 7.651278 6.258655
## 52 Tanzania G. m. morsitans W+/V- 3.036629 8.409595 5.262641
## 53 Tanzania G. m. morsitans W+/V- 6.619093 8.227372 5.545066
## 54 Tanzania G. m. morsitans W+/V- 3.134814 7.643453 5.961087
## 55 Tanzania G. m. morsitans W+/V- 3.338456 8.335257 6.065603
## 56 Tanzania G. m. morsitans W+/V- 2.898725 7.247482 6.140878
## 57 Tanzania G. m. morsitans W+/V- 3.313023 8.423574 5.268142
## 58 Tanzania G. m. morsitans W+/V- 3.238548 8.416973 5.411659
## 59 Tanzania G. m. morsitans W+/V- 2.944483 7.274158 6.183001
## 60 Tanzania G. m. morsitans W+/V- 2.942504 7.635484 6.238228
## 61 Zambia G. m. morsitans W+/V- 2.517987 14.878522 6.338950
## 62 Zambia G. m. morsitans W+/V- 2.517987 14.878522 6.445490
## 63 Zambia G. m. morsitans W+/V- 2.343999 14.146128 6.789324
## 64 Zambia G. m. morsitans W+/V- 1.989450 15.534534 5.620234
## 65 Zambia G. m. morsitans W+/V- 2.517987 14.878522 6.340893
## 66 Zambia G. m. morsitans W+/V- 1.954243 8.864511 3.496597
## 67 Zimbabwe G. m. morsitans W+/V+ 3.543571 3.989450 5.210836
## 68 Zimbabwe G. m. morsitans W+/V- 4.406199 7.038223 4.700089
## 69 Zimbabwe G. m. morsitans W+/V+ 3.393751 8.936514 6.948944
## 70 Zimbabwe G. m. morsitans W+/V- 3.189771 9.643453 4.017676
## 71 Zimbabwe G. m. morsitans W+/V+ 3.055378 6.523746 5.061756
## 72 Zimbabwe G. m. morsitans W-/V+ 3.729165 4.501333 6.159304
## 73 Zimbabwe G. m. morsitans W+/V- 3.982271 6.248464 3.628272
## 74 Zimbabwe G. m. morsitans W+/V- 2.896526 7.261976 4.395513
## 75 Zimbabwe G. m. morsitans W+/V+ 3.055378 6.523746 4.963742
## 76 Zambia G. m. morsitans W+/V+ 2.496376 8.312177 3.718736
## 77 Zambia G. m. morsitans W+/V+ 2.496376 8.312177 4.014393
## 78 Zambia G. m. morsitans W+/V+ 2.496376 8.312177 4.016192
## 79 Zimbabwe G. m. morsitans W+/V+ 3.845098 7.709270 4.017656
## 80 Zimbabwe G. m. morsitans W+/V+ 2.719331 7.360215 4.150421
## 81 Zimbabwe G. m. morsitans W+/V+ 3.845098 7.709270 4.158713
## 82 Zimbabwe G. m. morsitans W+/V- 2.857332 6.900913 4.304863
## 83 Zimbabwe G. m. morsitans W+/V+ 4.112605 4.377306 3.264307
## 84 Zimbabwe G. m. morsitans W+/V+ 4.112605 4.377306 3.372475
## 85 Zimbabwe G. m. morsitans W+/V+ 4.112605 4.377306 3.446208
## 86 Zimbabwe G. m. morsitans W+/V+ 2.938520 6.639486 4.130066
## 87 Zimbabwe G. m. morsitans W+/V- 3.031812 9.655138 4.228599
## 88 Zimbabwe G. m. morsitans W+/V- 4.176091 4.449633 4.999776
## 89 Zimbabwe G. m. morsitans W+/V- 3.192010 7.803457 4.300718
## 90 Zimbabwe G. m. morsitans W+/V+ 3.845098 7.709270 4.133931
## 91 Zambia G. m. morsitans W+/V- 2.124504 15.658965 5.505514
## 92 Zambia G. m. morsitans W+/V- 2.121888 8.158362 6.220152
## 93 Zambia G. m. morsitans W+/V- 1.684845 7.835056 6.172877
## 94 Zambia G. m. morsitans W+/V- 1.934498 8.134814 6.348968
## 95 Zambia G. m. morsitans W+/V- 1.974972 8.905256 3.378617
## 96 Zimbabwe G. m. morsitans W+/V+ 7.571476 NA 3.388551
## 97 Zimbabwe G. m. morsitans W+/V+ 3.857332 NA 6.626950
## 98 Zimbabwe G. m. morsitans W+/V+ 3.857332 NA 6.676298
## 99 Zimbabwe G. m. morsitans W+/V+ 11.320562 NA 6.333494
## 100 Zimbabwe G. m. morsitans W+/V+ 2.775246 NA 6.894753
## 101 Zimbabwe G. m. morsitans W+/V+ 2.775246 NA 6.904206
## 102 Zimbabwe G. m. morsitans W+/V+ 2.775246 NA 6.919518
## 103 Zimbabwe G. m. morsitans W+/V- 3.453318 NA 4.031085
## 104 Zimbabwe G. m. morsitans W+/V- 3.372912 NA 4.429025
## 105 Zimbabwe G. m. morsitans W+/V- 3.460597 NA 4.482710
## 106 Zimbabwe G. m. morsitans W+/V- 3.946452 NA 4.786975
## 107 Zimbabwe G. m. morsitans W+/V- 4.052309 NA 4.916273
## 108 Zimbabwe G. m. morsitans W+/V- 4.401401 NA 4.712715
## 109 Zimbabwe G. m. morsitans W+/V- 4.139879 NA 5.090553
## 110 Zimbabwe G. m. morsitans W+/V- 3.896526 NA 4.893740
## 111 Zimbabwe G. m. morsitans W+/V- 4.465383 NA 4.822142
## 112 Zimbabwe G. m. morsitans W+/V- 3.824776 NA 4.938539
## 113 Zimbabwe G. m. morsitans W+/V- 3.631444 NA 3.987842
## 114 Zimbabwe G. m. morsitans W+/V- 3.681241 NA 4.183068
## 115 Zimbabwe G. m. morsitans W+/V- 2.792392 NA 4.449532
## 116 Zimbabwe G. m. morsitans W+/V- 4.115278 NA 4.863471
## 117 Tanzania G. pallidipes W+/V+ 3.725912 5.745075 6.190086
## 118 Tanzania G. pallidipes W+/V+ 3.725912 5.745075 6.211020
## 119 Tanzania G. pallidipes W-/V+ 4.610660 NA 5.980494
## 120 Tanzania G. pallidipes W-/V+ 4.577032 NA 6.067431
## 121 Tanzania G. pallidipes W-/V+ 4.583879 NA 6.272031
## 122 Tanzania G. pallidipes W-/V+ 3.468347 NA 3.377431
## 123 Zambia G. pallidipes W-/V+ 3.107210 NA 3.755588
## Wigglesworthia
## 1 6.837400
## 2 5.479647
## 3 5.528074
## 4 7.157453
## 5 6.218625
## 6 6.208117
## 7 NA
## 8 NA
## 9 NA
## 10 NA
## 11 5.733434
## 12 5.135913
## 13 5.497730
## 14 5.893921
## 15 6.963434
## 16 5.291318
## 17 NA
## 18 NA
## 19 NA
## 20 NA
## 21 NA
## 22 4.943486
## 23 4.708313
## 24 NA
## 25 6.678722
## 26 5.339160
## 27 6.310350
## 28 6.443300
## 29 6.116602
## 30 6.804350
## 31 10.191603
## 32 5.459354
## 33 5.899101
## 34 5.952105
## 35 6.344745
## 36 5.784220
## 37 7.800503
## 38 10.062864
## 39 9.388388
## 40 6.937702
## 41 6.805077
## 42 6.182330
## 43 9.838294
## 44 9.297119
## 45 7.098055
## 46 7.124997
## 47 NA
## 48 NA
## 49 NA
## 50 NA
## 51 NA
## 52 NA
## 53 NA
## 54 NA
## 55 NA
## 56 NA
## 57 NA
## 58 NA
## 59 NA
## 60 NA
## 61 NA
## 62 NA
## 63 NA
## 64 NA
## 65 NA
## 66 NA
## 67 NA
## 68 NA
## 69 NA
## 70 NA
## 71 NA
## 72 NA
## 73 5.789754
## 74 6.327612
## 75 6.663816
## 76 6.023311
## 77 6.096269
## 78 6.246218
## 79 6.575373
## 80 6.232853
## 81 6.097164
## 82 7.148731
## 83 8.673505
## 84 8.657838
## 85 8.641584
## 86 6.787858
## 87 6.356982
## 88 10.067865
## 89 NA
## 90 NA
## 91 6.393184
## 92 6.094564
## 93 NA
## 94 NA
## 95 NA
## 96 5.400720
## 97 9.051741
## 98 9.070961
## 99 9.454929
## 100 9.808075
## 101 9.869617
## 102 9.788180
## 103 9.291721
## 104 9.342932
## 105 9.329248
## 106 10.085327
## 107 10.505882
## 108 9.987575
## 109 9.996559
## 110 10.113915
## 111 9.959726
## 112 9.871988
## 113 9.995597
## 114 9.999424
## 115 9.864506
## 116 10.077351
## 117 5.537838
## 118 5.731851
## 119 8.500074
## 120 8.375135
## 121 NA
## 122 8.915657
## 123 8.360722

#GpSGHV
fig1$Country=as.factor(fig1$Country)
fig1$Country <- relevel(fig1$Country, ref = "Burkina Faso")
gpsghv <- glm (GpSGHV~Country, data = fig1)
summary(gpsghv)

##
## Call:
## glm(formula = GpSGHV ~ Country, data = fig1)
##
## Deviance Residuals:
## Min 1Q Median 3Q Max
## -1.6081 -0.7318 -0.3762 0.0860 7.2631
##
## Coefficients:
## Estimate Std. Error t value Pr(>|t|)
## (Intercept) 3.1336 1.0711 2.925 0.00413 **
## CountrySouth Africa 0.3798 1.1234 0.338 0.73591
## CountryTanzania 0.6554 1.1132 0.589 0.55713
## CountryZambia -0.7232 1.1324 -0.639 0.52427
## CountryZimbabwe 0.9239 1.0891 0.848 0.39800
## ---
## Signif. codes: 0 '***' 0.001 '**' 0.01 '*' 0.05 '.' 0.1 ' ' 1
##
## (Dispersion parameter for gaussian family taken to be 2.294679)
##
## Null deviance: 308.02 on 122 degrees of freedom
## Residual deviance: 270.77 on 118 degrees of freedom
## AIC: 458.12
##
## Number of Fisher Scoring iterations: 2

fig1$Country <- relevel(fig1$Country, ref = "South Africa")
gpsghv <- glm (GpSGHV~Country, data = fig1)
summary(gpsghv)

##
## Call:
## glm(formula = GpSGHV ~ Country, data = fig1)
##
## Deviance Residuals:
## Min 1Q Median 3Q Max
## -1.6081 -0.7318 -0.3762 0.0860 7.2631
##
## Coefficients:
## Estimate Std. Error t value Pr(>|t|)
## (Intercept) 3.5134 0.3387 10.372 <2e-16 ***
## CountryBurkina Faso -0.3798 1.1234 -0.338 0.7359
## CountryTanzania 0.2756 0.4544 0.606 0.5454
## CountryZambia -1.1030 0.4997 -2.207 0.0292 *
## CountryZimbabwe 0.5441 0.3920 1.388 0.1677
## ---
## Signif. codes: 0 '***' 0.001 '**' 0.01 '*' 0.05 '.' 0.1 ' ' 1
##
## (Dispersion parameter for gaussian family taken to be 2.294679)
##
## Null deviance: 308.02 on 122 degrees of freedom
## Residual deviance: 270.77 on 118 degrees of freedom
## AIC: 458.12
##
## Number of Fisher Scoring iterations: 2

fig1$Country <- relevel(fig1$Country, ref = "Tanzania")
gpsghv <- glm (GpSGHV~Country, data = fig1)
summary(gpsghv)

##
## Call:
## glm(formula = GpSGHV ~ Country, data = fig1)
##
## Deviance Residuals:
## Min 1Q Median 3Q Max
## -1.6081 -0.7318 -0.3762 0.0860 7.2631
##
## Coefficients:
## Estimate Std. Error t value Pr(>|t|)
## (Intercept) 3.7890 0.3030 12.506 < 2e-16 ***
## CountrySouth Africa -0.2756 0.4544 -0.606 0.54535
## CountryBurkina Faso -0.6554 1.1132 -0.589 0.55713
## CountryZambia -1.3786 0.4762 -2.895 0.00452 **
## CountryZimbabwe 0.2685 0.3615 0.743 0.45914
## ---
## Signif. codes: 0 '***' 0.001 '**' 0.01 '*' 0.05 '.' 0.1 ' ' 1
##
## (Dispersion parameter for gaussian family taken to be 2.294679)
##
## Null deviance: 308.02 on 122 degrees of freedom
## Residual deviance: 270.77 on 118 degrees of freedom
## AIC: 458.12
##
## Number of Fisher Scoring iterations: 2

fig1$Country <- relevel(fig1$Country, ref = "Zambia")
gpsghv <- glm (GpSGHV~Country, data = fig1)
summary(gpsghv)

##
## Call:
## glm(formula = GpSGHV ~ Country, data = fig1)
##
## Deviance Residuals:
## Min 1Q Median 3Q Max
## -1.6081 -0.7318 -0.3762 0.0860 7.2631
##
## Coefficients:
## Estimate Std. Error t value Pr(>|t|)
## (Intercept) 2.4103 0.3674 6.561 1.49e-09 ***
## CountryTanzania 1.3786 0.4762 2.895 0.004518 **
## CountrySouth Africa 1.1030 0.4997 2.207 0.029228 *
## CountryBurkina Faso 0.7232 1.1324 0.639 0.524275
## CountryZimbabwe 1.6471 0.4170 3.950 0.000133 ***
## ---
## Signif. codes: 0 '***' 0.001 '**' 0.01 '*' 0.05 '.' 0.1 ' ' 1
##
## (Dispersion parameter for gaussian family taken to be 2.294679)
##
## Null deviance: 308.02 on 122 degrees of freedom
## Residual deviance: 270.77 on 118 degrees of freedom
## AIC: 458.12
##
## Number of Fisher Scoring iterations: 2

fig1$Country <- relevel(fig1$Country, ref = "Zimbabwe")
gpsghv <- glm (GpSGHV~Country, data = fig1)
summary(gpsghv)

##
## Call:
## glm(formula = GpSGHV ~ Country, data = fig1)
##
## Deviance Residuals:
## Min 1Q Median 3Q Max
## -1.6081 -0.7318 -0.3762 0.0860 7.2631
##
## Coefficients:
## Estimate Std. Error t value Pr(>|t|)
## (Intercept) 4.0575 0.1972 20.574 < 2e-16 ***
## CountryZambia -1.6471 0.4170 -3.950 0.000133 ***
## CountryTanzania -0.2685 0.3615 -0.743 0.459138
## CountrySouth Africa -0.5441 0.3920 -1.388 0.167697
## CountryBurkina Faso -0.9239 1.0891 -0.848 0.398001
## ---
## Signif. codes: 0 '***' 0.001 '**' 0.01 '*' 0.05 '.' 0.1 ' ' 1
##
## (Dispersion parameter for gaussian family taken to be 2.294679)
##
## Null deviance: 308.02 on 122 degrees of freedom
## Residual deviance: 270.77 on 118 degrees of freedom
## AIC: 458.12
##
## Number of Fisher Scoring iterations: 2

#---------------------------------------------------------
#wolbachia
fig1$Country <- relevel(fig1$Country, ref = "Burkina Faso")
wol <- glm (Wolbachia~Country, data = fig1)
summary(wol)

##
## Call:
## glm(formula = Wolbachia ~ Country, data = fig1)
##
## Deviance Residuals:
## Min 1Q Median 3Q Max
## -3.1943 -1.9716 0.1638 0.8887 5.0424
##
## Coefficients:
## Estimate Std. Error t value Pr(>|t|)
## (Intercept) 6.5107 0.3191 20.405 < 2e-16 ***
## CountryZambia 4.5187 0.5862 7.709 1.71e-11 ***
## CountryTanzania 1.4031 0.5348 2.624 0.0102 *
## CountrySouth Africa -0.5791 0.5628 -1.029 0.3063
## ---
## Signif. codes: 0 '***' 0.001 '**' 0.01 '*' 0.05 '.' 0.1 ' ' 1
##
## (Dispersion parameter for gaussian family taken to be 3.86852)
##
## Null deviance: 628.66 on 92 degrees of freedom
## Residual deviance: 344.30 on 89 degrees of freedom
## (30 observations deleted due to missingness)
## AIC: 395.65
##
## Number of Fisher Scoring iterations: 2

fig1$Country <- relevel(fig1$Country, ref = "South Africa")
wol <- glm (Wolbachia~Country, data = fig1)
summary(wol)

##
## Call:
## glm(formula = Wolbachia ~ Country, data = fig1)
##
## Deviance Residuals:
## Min 1Q Median 3Q Max
## -3.1943 -1.9716 0.1638 0.8887 5.0424
##
## Coefficients:
## Estimate Std. Error t value Pr(>|t|)
## (Intercept) 5.9316 0.4636 12.795 < 2e-16 ***
## CountryZimbabwe 0.5791 0.5628 1.029 0.30630
## CountryZambia 5.0977 0.6758 7.543 3.72e-11 ***
## CountryTanzania 1.9822 0.6318 3.138 0.00231 **
## ---
## Signif. codes: 0 '***' 0.001 '**' 0.01 '*' 0.05 '.' 0.1 ' ' 1
##
## (Dispersion parameter for gaussian family taken to be 3.86852)
##
## Null deviance: 628.66 on 92 degrees of freedom
## Residual deviance: 344.30 on 89 degrees of freedom
## (30 observations deleted due to missingness)
## AIC: 395.65
##
## Number of Fisher Scoring iterations: 2

fig1$Country <- relevel(fig1$Country, ref = "Tanzania")
wol <- glm (Wolbachia~Country, data = fig1)
summary(wol)

##
## Call:
## glm(formula = Wolbachia ~ Country, data = fig1)
##
## Deviance Residuals:
## Min 1Q Median 3Q Max
## -3.1943 -1.9716 0.1638 0.8887 5.0424
##
## Coefficients:
## Estimate Std. Error t value Pr(>|t|)
## (Intercept) 7.9138 0.4292 18.438 < 2e-16 ***
## CountrySouth Africa -1.9822 0.6318 -3.138 0.00231 **
## CountryZimbabwe -1.4031 0.5348 -2.624 0.01024 *
## CountryZambia 3.1155 0.6527 4.773 7.07e-06 ***
## ---
## Signif. codes: 0 '***' 0.001 '**' 0.01 '*' 0.05 '.' 0.1 ' ' 1
##
## (Dispersion parameter for gaussian family taken to be 3.86852)
##
## Null deviance: 628.66 on 92 degrees of freedom
## Residual deviance: 344.30 on 89 degrees of freedom
## (30 observations deleted due to missingness)
## AIC: 395.65
##
## Number of Fisher Scoring iterations: 2

fig1$Country <- relevel(fig1$Country, ref = "Zambia")
wol <- glm (Wolbachia~Country, data = fig1)
summary(wol)

##
## Call:
## glm(formula = Wolbachia ~ Country, data = fig1)
##
## Deviance Residuals:
## Min 1Q Median 3Q Max
## -3.1943 -1.9716 0.1638 0.8887 5.0424
##
## Coefficients:
## Estimate Std. Error t value Pr(>|t|)
## (Intercept) 11.0293 0.4917 22.430 < 2e-16 ***
## CountryTanzania -3.1155 0.6527 -4.773 7.07e-06 ***
## CountrySouth Africa -5.0977 0.6758 -7.543 3.72e-11 ***
## CountryZimbabwe -4.5187 0.5862 -7.709 1.71e-11 ***
## ---
## Signif. codes: 0 '***' 0.001 '**' 0.01 '*' 0.05 '.' 0.1 ' ' 1
##
## (Dispersion parameter for gaussian family taken to be 3.86852)
##
## Null deviance: 628.66 on 92 degrees of freedom
## Residual deviance: 344.30 on 89 degrees of freedom
## (30 observations deleted due to missingness)
## AIC: 395.65
##
## Number of Fisher Scoring iterations: 2

fig1$Country <- relevel(fig1$Country, ref = "Zimbabwe")
wol <- glm (Wolbachia~Country, data = fig1)
summary(wol)

##
## Call:
## glm(formula = Wolbachia ~ Country, data = fig1)
##
## Deviance Residuals:
## Min 1Q Median 3Q Max
## -3.1943 -1.9716 0.1638 0.8887 5.0424
##
## Coefficients:
## Estimate Std. Error t value Pr(>|t|)
## (Intercept) 6.5107 0.3191 20.405 < 2e-16 ***
## CountryZambia 4.5187 0.5862 7.709 1.71e-11 ***
## CountryTanzania 1.4031 0.5348 2.624 0.0102 *
## CountrySouth Africa -0.5791 0.5628 -1.029 0.3063
## ---
## Signif. codes: 0 '***' 0.001 '**' 0.01 '*' 0.05 '.' 0.1 ' ' 1
##
## (Dispersion parameter for gaussian family taken to be 3.86852)
##
## Null deviance: 628.66 on 92 degrees of freedom
## Residual deviance: 344.30 on 89 degrees of freedom
## (30 observations deleted due to missingness)
## AIC: 395.65
##
## Number of Fisher Scoring iterations: 2

#--------------------------------------------------------
#Wigglesworthia
fig1$Country <- relevel(fig1$Country, ref = "Burkina Faso")
wigg <- glm (Wigglesworthia~Country, data = fig1)
summary(wigg)

##
## Call:
## glm(formula = Wigglesworthia ~ Country, data = fig1)
##
## Deviance Residuals:
## Min 1Q Median 3Q Max
## -2.9057 -1.4218 0.1384 1.5404 2.2611
##
## Coefficients:
## Estimate Std. Error t value Pr(>|t|)
## (Intercept) 4.708 1.518 3.102 0.0027 **
## CountryZimbabwe 3.537 1.533 2.308 0.0237 *
## CountryZambia 1.766 1.610 1.097 0.2762
## CountryTanzania 2.251 1.600 1.407 0.1634
## CountrySouth Africa 1.208 1.580 0.765 0.4468
## ---
## Signif. codes: 0 '***' 0.001 '**' 0.01 '*' 0.05 '.' 0.1 ' ' 1
##
## (Dispersion parameter for gaussian family taken to be 2.303399)
##
## Null deviance: 252.17 on 80 degrees of freedom
## Residual deviance: 175.06 on 76 degrees of freedom
## (42 observations deleted due to missingness)
## AIC: 304.29
##
## Number of Fisher Scoring iterations: 2

fig1$Country <- relevel(fig1$Country, ref = "South Africa")
wigg <- glm (Wigglesworthia~Country, data = fig1)
summary(wigg)

##
## Call:
## glm(formula = Wigglesworthia ~ Country, data = fig1)
##
## Deviance Residuals:
## Min 1Q Median 3Q Max
## -2.9057 -1.4218 0.1384 1.5404 2.2611
##
## Coefficients:
## Estimate Std. Error t value Pr(>|t|)
## (Intercept) 5.9162 0.4381 13.504 < 2e-16 ***
## CountryBurkina Faso -1.2079 1.5797 -0.765 0.447
## CountryZimbabwe 2.3286 0.4869 4.782 8.32e-06 ***
## CountryZambia 0.5578 0.6927 0.805 0.423
## CountryTanzania 1.0434 0.6692 1.559 0.123
## ---
## Signif. codes: 0 '***' 0.001 '**' 0.01 '*' 0.05 '.' 0.1 ' ' 1
##
## (Dispersion parameter for gaussian family taken to be 2.303399)
##
## Null deviance: 252.17 on 80 degrees of freedom
## Residual deviance: 175.06 on 76 degrees of freedom
## (42 observations deleted due to missingness)
## AIC: 304.29
##
## Number of Fisher Scoring iterations: 2

fig1$Country <- relevel(fig1$Country, ref = "Tanzania")
wigg <- glm (Wigglesworthia~Country, data = fig1)
summary(wigg)

##
## Call:
## glm(formula = Wigglesworthia ~ Country, data = fig1)
##
## Deviance Residuals:
## Min 1Q Median 3Q Max
## -2.9057 -1.4218 0.1384 1.5404 2.2611
##
## Coefficients:
## Estimate Std. Error t value Pr(>|t|)
## (Intercept) 6.9597 0.5059 13.757 <2e-16 ***
## CountrySouth Africa -1.0434 0.6692 -1.559 0.1231
## CountryBurkina Faso -2.2513 1.5998 -1.407 0.1634
## CountryZimbabwe 1.2852 0.5487 2.342 0.0218 *
## CountryZambia -0.4856 0.7375 -0.659 0.5122
## ---
## Signif. codes: 0 '***' 0.001 '**' 0.01 '*' 0.05 '.' 0.1 ' ' 1
##
## (Dispersion parameter for gaussian family taken to be 2.303399)
##
## Null deviance: 252.17 on 80 degrees of freedom
## Residual deviance: 175.06 on 76 degrees of freedom
## (42 observations deleted due to missingness)
## AIC: 304.29
##
## Number of Fisher Scoring iterations: 2

fig1$Country <- relevel(fig1$Country, ref = "Zambia")
wigg <- glm (Wigglesworthia~Country, data = fig1)
summary(wigg)

##
## Call:
## glm(formula = Wigglesworthia ~ Country, data = fig1)
##
## Deviance Residuals:
## Min 1Q Median 3Q Max
## -2.9057 -1.4218 0.1384 1.5404 2.2611
##
## Coefficients:
## Estimate Std. Error t value Pr(>|t|)
## (Intercept) 6.4740 0.5366 12.065 < 2e-16 ***
## CountryTanzania 0.4856 0.7375 0.659 0.51219
## CountrySouth Africa -0.5578 0.6927 -0.805 0.42321
## CountryBurkina Faso -1.7657 1.6098 -1.097 0.27616
## CountryZimbabwe 1.7708 0.5771 3.068 0.00298 **
## ---
## Signif. codes: 0 '***' 0.001 '**' 0.01 '*' 0.05 '.' 0.1 ' ' 1
##
## (Dispersion parameter for gaussian family taken to be 2.303399)
##
## Null deviance: 252.17 on 80 degrees of freedom
## Residual deviance: 175.06 on 76 degrees of freedom
## (42 observations deleted due to missingness)
## AIC: 304.29
##
## Number of Fisher Scoring iterations: 2

fig1$Country <- relevel(fig1$Country, ref = "Zimbabwe")
wigg <- glm (Wigglesworthia~Country, data = fig1)
summary(wigg)

##
## Call:
## glm(formula = Wigglesworthia ~ Country, data = fig1)
##
## Deviance Residuals:
## Min 1Q Median 3Q Max
## -2.9057 -1.4218 0.1384 1.5404 2.2611
##
## Coefficients:
## Estimate Std. Error t value Pr(>|t|)
## (Intercept) 8.2448 0.2125 38.796 < 2e-16 ***
## CountryZambia -1.7708 0.5771 -3.068 0.00298 **
## CountryTanzania -1.2852 0.5487 -2.342 0.02180 *
## CountrySouth Africa -2.3286 0.4869 -4.782 8.32e-06 ***
## CountryBurkina Faso -3.5365 1.5325 -2.308 0.02374 *
## ---
## Signif. codes: 0 '***' 0.001 '**' 0.01 '*' 0.05 '.' 0.1 ' ' 1
##
## (Dispersion parameter for gaussian family taken to be 2.303399)
##
## Null deviance: 252.17 on 80 degrees of freedom
## Residual deviance: 175.06 on 76 degrees of freedom
## (42 observations deleted due to missingness)
## AIC: 304.29
##
## Number of Fisher Scoring iterations: 2

#------------------------------------------------------------

fig1$Country <- relevel(fig1$Country, ref = "Burkina Faso")
sod <- glm (Sodalis~Country, data = fig1)
summary(sod)

##
## Call:
## glm(formula = Sodalis ~ Country, data = fig1)
##
## Deviance Residuals:
## Min 1Q Median 3Q Max
## -2.30181 -0.87642 -0.07664 0.83645 2.52354
##
## Coefficients:
## Estimate Std. Error t value Pr(>|t|)
## (Intercept) 3.2898 0.8031 4.096 7.76e-05 ***
## CountryZimbabwe 1.7649 0.8166 2.161 0.0327 *
## CountryZambia 2.0500 0.8490 2.415 0.0173 *
## CountryTanzania 2.3264 0.8346 2.787 0.0062 **
## CountrySouth Africa 0.9759 0.8443 1.156 0.2501
## ---
## Signif. codes: 0 '***' 0.001 '**' 0.01 '*' 0.05 '.' 0.1 ' ' 1
##
## (Dispersion parameter for gaussian family taken to be 1.289906)
##
## Null deviance: 178.24 on 121 degrees of freedom
## Residual deviance: 150.92 on 117 degrees of freedom
## (1 observation deleted due to missingness)
## AIC: 384.17
##
## Number of Fisher Scoring iterations: 2

fig1$Country <- relevel(fig1$Country, ref = "South Africa")
sod <- glm (Sodalis~Country, data = fig1)
summary(sod)

##
## Call:
## glm(formula = Sodalis ~ Country, data = fig1)
##
## Deviance Residuals:
## Min 1Q Median 3Q Max
## -2.30181 -0.87642 -0.07664 0.83645 2.52354
##
## Coefficients:
## Estimate Std. Error t value Pr(>|t|)
## (Intercept) 4.2658 0.2606 16.372 < 2e-16 ***
## CountryBurkina Faso -0.9759 0.8443 -1.156 0.250069
## CountryZimbabwe 0.7889 0.2996 2.633 0.009596 **
## CountryZambia 1.0741 0.3792 2.833 0.005436 **
## CountryTanzania 1.3505 0.3457 3.907 0.000157 ***
## ---
## Signif. codes: 0 '***' 0.001 '**' 0.01 '*' 0.05 '.' 0.1 ' ' 1
##
## (Dispersion parameter for gaussian family taken to be 1.289906)
##
## Null deviance: 178.24 on 121 degrees of freedom
## Residual deviance: 150.92 on 117 degrees of freedom
## (1 observation deleted due to missingness)
## AIC: 384.17
##
## Number of Fisher Scoring iterations: 2

fig1$Country <- relevel(fig1$Country, ref = "Tanzania")
sod <- glm (Sodalis~Country, data = fig1)
summary(sod)

##
## Call:
## glm(formula = Sodalis ~ Country, data = fig1)
##
## Deviance Residuals:
## Min 1Q Median 3Q Max
## -2.30181 -0.87642 -0.07664 0.83645 2.52354
##
## Coefficients:
## Estimate Std. Error t value Pr(>|t|)
## (Intercept) 5.6162 0.2271 24.725 < 2e-16 ***
## CountrySouth Africa -1.3505 0.3457 -3.907 0.000157 ***
## CountryBurkina Faso -2.3264 0.8346 -2.787 0.006201 **
## CountryZimbabwe -0.5615 0.2710 -2.072 0.040483 *
## CountryZambia -0.2764 0.3570 -0.774 0.440453
## ---
## Signif. codes: 0 '***' 0.001 '**' 0.01 '*' 0.05 '.' 0.1 ' ' 1
##
## (Dispersion parameter for gaussian family taken to be 1.289906)
##
## Null deviance: 178.24 on 121 degrees of freedom
## Residual deviance: 150.92 on 117 degrees of freedom
## (1 observation deleted due to missingness)
## AIC: 384.17
##
## Number of Fisher Scoring iterations: 2

fig1$Country <- relevel(fig1$Country, ref = "Zambia")
sod <- glm (Sodalis~Country, data = fig1)
summary(sod)

##
## Call:
## glm(formula = Sodalis ~ Country, data = fig1)
##
## Deviance Residuals:
## Min 1Q Median 3Q Max
## -2.30181 -0.87642 -0.07664 0.83645 2.52354
##
## Coefficients:
## Estimate Std. Error t value Pr(>|t|)
## (Intercept) 5.3399 0.2755 19.385 < 2e-16 ***
## CountryTanzania 0.2764 0.3570 0.774 0.44045
## CountrySouth Africa -1.0741 0.3792 -2.833 0.00544 **
## CountryBurkina Faso -2.0500 0.8490 -2.415 0.01730 *
## CountryZimbabwe -0.2852 0.3126 -0.912 0.36359
## ---
## Signif. codes: 0 '***' 0.001 '**' 0.01 '*' 0.05 '.' 0.1 ' ' 1
##
## (Dispersion parameter for gaussian family taken to be 1.289906)
##
## Null deviance: 178.24 on 121 degrees of freedom
## Residual deviance: 150.92 on 117 degrees of freedom
## (1 observation deleted due to missingness)
## AIC: 384.17
##
## Number of Fisher Scoring iterations: 2

fig1$Country <- relevel(fig1$Country, ref = "Zimbabwe")
sod <- glm (Sodalis~Country, data = fig1)
summary(sod)

##
## Call:
## glm(formula = Sodalis ~ Country, data = fig1)
##
## Deviance Residuals:
## Min 1Q Median 3Q Max
## -2.30181 -0.87642 -0.07664 0.83645 2.52354
##
## Coefficients:
## Estimate Std. Error t value Pr(>|t|)
## (Intercept) 5.0547 0.1479 34.186 <2e-16 ***
## CountryZambia 0.2852 0.3126 0.912 0.3636
## CountryTanzania 0.5615 0.2710 2.072 0.0405 *
## CountrySouth Africa -0.7889 0.2996 -2.633 0.0096 **
## CountryBurkina Faso -1.7649 0.8166 -2.161 0.0327 *
## ---
## Signif. codes: 0 '***' 0.001 '**' 0.01 '*' 0.05 '.' 0.1 ' ' 1
##
## (Dispersion parameter for gaussian family taken to be 1.289906)
##
## Null deviance: 178.24 on 121 degrees of freedom
## Residual deviance: 150.92 on 117 degrees of freedom
## (1 observation deleted due to missingness)
## AIC: 384.17
##
## Number of Fisher Scoring iterations: 2

## Prepare supplementary figure 4 and its statistics

fig1 <- read.csv("virus_symb_all_20210613_all3.csv")
fig1

## Country Species Samples GpSGHV Wolbachia Sodalis
## 1 South Africa G. austeni W+/V+ 3.071882 5.291369 5.226169
## 2 South Africa G. austeni W+/V+ 3.631444 5.563006 2.453318
## 3 South Africa G. austeni W+/V+ 3.070407 4.121888 3.376584
## 4 South Africa G. austeni W+/V+ 3.071882 5.291369 5.138430
## 5 South Africa G. austeni W+/V- 6.330414 6.542078 5.056997
## 6 South Africa G. austeni W+/V- 6.322219 6.544068 5.070590
## 7 South Africa G. austeni W+/V- 3.070407 7.722634 4.074324
## 8 South Africa G. austeni W+/V+ 3.071882 5.291369 5.240495
## 9 South Africa G. austeni W+/V- 3.082067 7.797960 4.176986
## 10 South Africa G. austeni W+/V- 6.340841 6.412293 5.103382
## 11 South Africa G. austeni W+/V- 2.606381 6.670246 3.527706
## 12 South Africa G. austeni W+/V+ 3.467164 7.808886 3.083206
## 13 South Africa G. austeni W+/V+ 3.098990 3.201943 3.387768
## 14 Tanzania G. austeni W+/V+ 3.735599 7.030195 3.340603
## 15 South Africa G. austeni W+/V+ 3.850033 6.418301 2.990989
## 16 South Africa G. austeni W+/V+ 3.152288 4.077731 3.357279
## 17 South Africa G. austeni W+/V- 1.905256 7.064458 3.804619
## 18 Tanzania G. austeni W+/V- 3.008600 12.956168 3.314423
## 19 South Africa G. austeni W+/V+ 2.775246 3.959995 NA
## 20 South Africa G. austeni W+/V+ 2.857332 6.989450 6.789324
## 21 South Africa G. austeni W+/V+ 2.716003 NA 6.623851
## 22 South Africa G. austeni W+/V+ 2.775246 NA 2.567822
## 23 Burkina Faso G. medicorum W-/V+ 3.353339 NA 3.501522
## 24 Burkina Faso G. medicorum W-/V+ 2.913814 NA 3.078152
## 25 Zambia G. m. morsitans W+/V- 3.695482 11.193125 6.979752
## 26 Zimbabwe G. m. morsitans W+/V- 4.331225 6.991226 4.705386
## 27 Zimbabwe G. m. morsitans W+/V+ 3.430720 4.483445 5.116050
## 28 Zimbabwe G. m. morsitans W+/V+ 2.938520 6.639486 4.256616
## 29 Zimbabwe G. m. morsitans W+/V+ 3.393751 8.936514 6.961294
## 30 Zimbabwe G. m. morsitans W+/V- 2.878522 7.510009 7.576463
## 31 Zimbabwe G. m. morsitans W+/V- 4.264818 3.889862 6.201792
## 32 Tanzania G. m. morsitans W+/V- 6.561578 8.147367 5.716086
## 33 Zambia G. m. morsitans W+/V+ 3.001734 8.466571 5.635447
## 34 Zimbabwe G. m. morsitans W+/V+ 3.385964 7.399328 5.896440
## 35 Zimbabwe G. m. morsitans W+/V- 3.186391 7.230449 3.827807
## 36 Zimbabwe G. m. morsitans W+/V+ 2.719331 7.360215 3.893365
## 37 Zimbabwe G. m. morsitans W+/V- 3.108565 7.845098 4.668694
## 38 Zimbabwe G. m. morsitans W-/V+ 3.748188 4.705864 6.200526
## 39 Zimbabwe G. m. morsitans W+/V+ 11.320562 4.071882 6.256775
## 40 Zimbabwe G. m. morsitans W+/V+ 3.880814 6.806180 6.414824
## 41 Zimbabwe G. m. morsitans W+/V+ 3.880814 6.806180 6.518537
## 42 Zimbabwe G. m. morsitans W+/V+ 3.393751 8.936514 6.929733
## 43 Zimbabwe G. m. morsitans W+/V- 6.001734 3.985875 4.535826
## 44 Zimbabwe G. m. morsitans W+/V+ 11.320562 4.071882 6.342526
## 45 Tanzania G. m. morsitans W+/V- 3.356408 8.372175 5.854843
## 46 Tanzania G. m. morsitans W+/V- 3.304491 8.268578 6.038572
## 47 Tanzania G. m. morsitans W+/V- 6.606381 8.058426 5.534126
## 48 Tanzania G. m. morsitans W+/V- 3.101747 7.673942 5.896745
## 49 Tanzania G. m. morsitans W+/V- 2.920123 7.265761 6.077336
## 50 Tanzania G. m. morsitans W+/V- 2.982271 7.662758 6.199656
## 51 Tanzania G. m. morsitans W+/V- 2.989450 7.651278 6.258655
## 52 Tanzania G. m. morsitans W+/V- 3.036629 8.409595 5.262641
## 53 Tanzania G. m. morsitans W+/V- 6.619093 8.227372 5.545066
## 54 Tanzania G. m. morsitans W+/V- 3.134814 7.643453 5.961087
## 55 Tanzania G. m. morsitans W+/V- 3.338456 8.335257 6.065603
## 56 Tanzania G. m. morsitans W+/V- 2.898725 7.247482 6.140878
## 57 Tanzania G. m. morsitans W+/V- 3.313023 8.423574 5.268142
## 58 Tanzania G. m. morsitans W+/V- 3.238548 8.416973 5.411659
## 59 Tanzania G. m. morsitans W+/V- 2.944483 7.274158 6.183001
## 60 Tanzania G. m. morsitans W+/V- 2.942504 7.635484 6.238228
## 61 Zambia G. m. morsitans W+/V- 2.517987 14.878522 6.338950
## 62 Zambia G. m. morsitans W+/V- 2.517987 14.878522 6.445490
## 63 Zambia G. m. morsitans W+/V- 2.343999 14.146128 6.789324
## 64 Zambia G. m. morsitans W+/V- 1.989450 15.534534 5.620234
## 65 Zambia G. m. morsitans W+/V- 2.517987 14.878522 6.340893
## 66 Zambia G. m. morsitans W+/V- 1.954243 8.864511 3.496597
## 67 Zimbabwe G. m. morsitans W+/V+ 3.543571 3.989450 5.210836
## 68 Zimbabwe G. m. morsitans W+/V- 4.406199 7.038223 4.700089
## 69 Zimbabwe G. m. morsitans W+/V+ 3.393751 8.936514 6.948944
## 70 Zimbabwe G. m. morsitans W+/V- 3.189771 9.643453 4.017676
## 71 Zimbabwe G. m. morsitans W+/V+ 3.055378 6.523746 5.061756
## 72 Zimbabwe G. m. morsitans W-/V+ 3.729165 4.501333 6.159304
## 73 Zimbabwe G. m. morsitans W+/V- 3.982271 6.248464 3.628272
## 74 Zimbabwe G. m. morsitans W+/V- 2.896526 7.261976 4.395513
## 75 Zimbabwe G. m. morsitans W+/V+ 3.055378 6.523746 4.963742
## 76 Zambia G. m. morsitans W+/V+ 2.496376 8.312177 3.718736
## 77 Zambia G. m. morsitans W+/V+ 2.496376 8.312177 4.014393
## 78 Zambia G. m. morsitans W+/V+ 2.496376 8.312177 4.016192
## 79 Zimbabwe G. m. morsitans W+/V+ 3.845098 7.709270 4.017656
## 80 Zimbabwe G. m. morsitans W+/V+ 2.719331 7.360215 4.150421
## 81 Zimbabwe G. m. morsitans W+/V+ 3.845098 7.709270 4.158713
## 82 Zimbabwe G. m. morsitans W+/V- 2.857332 6.900913 4.304863
## 83 Zimbabwe G. m. morsitans W+/V+ 4.112605 4.377306 3.264307
## 84 Zimbabwe G. m. morsitans W+/V+ 4.112605 4.377306 3.372475
## 85 Zimbabwe G. m. morsitans W+/V+ 4.112605 4.377306 3.446208
## 86 Zimbabwe G. m. morsitans W+/V+ 2.938520 6.639486 4.130066
## 87 Zimbabwe G. m. morsitans W+/V- 3.031812 9.655138 4.228599
## 88 Zimbabwe G. m. morsitans W+/V- 4.176091 4.449633 4.999776
## 89 Zimbabwe G. m. morsitans W+/V- 3.192010 7.803457 4.300718
## 90 Zimbabwe G. m. morsitans W+/V+ 3.845098 7.709270 4.133931
## 91 Zambia G. m. morsitans W+/V- 2.124504 15.658965 5.505514
## 92 Zambia G. m. morsitans W+/V- 2.121888 8.158362 6.220152
## 93 Zambia G. m. morsitans W+/V- 1.684845 7.835056 6.172877
## 94 Zambia G. m. morsitans W+/V- 1.934498 8.134814 6.348968
## 95 Zambia G. m. morsitans W+/V- 1.974972 8.905256 3.378617
## 96 Zimbabwe G. m. morsitans W+/V+ 7.571476 NA 3.388551
## 97 Zimbabwe G. m. morsitans W+/V+ 3.857332 NA 6.626950
## 98 Zimbabwe G. m. morsitans W+/V+ 3.857332 NA 6.676298
## 99 Zimbabwe G. m. morsitans W+/V+ 11.320562 NA 6.333494
## 100 Zimbabwe G. m. morsitans W+/V+ 2.775246 NA 6.894753
## 101 Zimbabwe G. m. morsitans W+/V+ 2.775246 NA 6.904206
## 102 Zimbabwe G. m. morsitans W+/V+ 2.775246 NA 6.919518
## 103 Zimbabwe G. m. morsitans W+/V- 3.453318 NA 4.031085
## 104 Zimbabwe G. m. morsitans W+/V- 3.372912 NA 4.429025
## 105 Zimbabwe G. m. morsitans W+/V- 3.460597 NA 4.482710
## 106 Zimbabwe G. m. morsitans W+/V- 3.946452 NA 4.786975
## 107 Zimbabwe G. m. morsitans W+/V- 4.052309 NA 4.916273
## 108 Zimbabwe G. m. morsitans W+/V- 4.401401 NA 4.712715
## 109 Zimbabwe G. m. morsitans W+/V- 4.139879 NA 5.090553
## 110 Zimbabwe G. m. morsitans W+/V- 3.896526 NA 4.893740
## 111 Zimbabwe G. m. morsitans W+/V- 4.465383 NA 4.822142
## 112 Zimbabwe G. m. morsitans W+/V- 3.824776 NA 4.938539
## 113 Zimbabwe G. m. morsitans W+/V- 3.631444 NA 3.987842
## 114 Zimbabwe G. m. morsitans W+/V- 3.681241 NA 4.183068
## 115 Zimbabwe G. m. morsitans W+/V- 2.792392 NA 4.449532
## 116 Zimbabwe G. m. morsitans W+/V- 4.115278 NA 4.863471
## 117 Tanzania G. pallidipes W+/V+ 3.725912 5.745075 6.190086
## 118 Tanzania G. pallidipes W+/V+ 3.725912 5.745075 6.211020
## 119 Tanzania G. pallidipes W-/V+ 4.610660 NA 5.980494
## 120 Tanzania G. pallidipes W-/V+ 4.577032 NA 6.067431
## 121 Tanzania G. pallidipes W-/V+ 4.583879 NA 6.272031
## 122 Tanzania G. pallidipes W-/V+ 3.468347 NA 3.377431
## 123 Zambia G. pallidipes W-/V+ 3.107210 NA 3.755588
## Wigglesworthia
## 1 6.837400
## 2 5.479647
## 3 5.528074
## 4 7.157453
## 5 6.218625
## 6 6.208117
## 7 NA
## 8 NA
## 9 NA
## 10 NA
## 11 5.733434
## 12 5.135913
## 13 5.497730
## 14 5.893921
## 15 6.963434
## 16 5.291318
## 17 NA
## 18 NA
## 19 NA
## 20 NA
## 21 NA
## 22 4.943486
## 23 4.708313
## 24 NA
## 25 6.678722
## 26 5.339160
## 27 6.310350
## 28 6.443300
## 29 6.116602
## 30 6.804350
## 31 10.191603
## 32 5.459354
## 33 5.899101
## 34 5.952105
## 35 6.344745
## 36 5.784220
## 37 7.800503
## 38 10.062864
## 39 9.388388
## 40 6.937702
## 41 6.805077
## 42 6.182330
## 43 9.838294
## 44 9.297119
## 45 7.098055
## 46 7.124997
## 47 NA
## 48 NA
## 49 NA
## 50 NA
## 51 NA
## 52 NA
## 53 NA
## 54 NA
## 55 NA
## 56 NA
## 57 NA
## 58 NA
## 59 NA
## 60 NA
## 61 NA
## 62 NA
## 63 NA
## 64 NA
## 65 NA
## 66 NA
## 67 NA
## 68 NA
## 69 NA
## 70 NA
## 71 NA
## 72 NA
## 73 5.789754
## 74 6.327612
## 75 6.663816
## 76 6.023311
## 77 6.096269
## 78 6.246218
## 79 6.575373
## 80 6.232853
## 81 6.097164
## 82 7.148731
## 83 8.673505
## 84 8.657838
## 85 8.641584
## 86 6.787858
## 87 6.356982
## 88 10.067865
## 89 NA
## 90 NA
## 91 6.393184
## 92 6.094564
## 93 NA
## 94 NA
## 95 NA
## 96 5.400720
## 97 9.051741
## 98 9.070961
## 99 9.454929
## 100 9.808075
## 101 9.869617
## 102 9.788180
## 103 9.291721
## 104 9.342932
## 105 9.329248
## 106 10.085327
## 107 10.505882
## 108 9.987575
## 109 9.996559
## 110 10.113915
## 111 9.959726
## 112 9.871988
## 113 9.995597
## 114 9.999424
## 115 9.864506
## 116 10.077351
## 117 5.537838
## 118 5.731851
## 119 8.500074
## 120 8.375135
## 121 NA
## 122 8.915657
## 123 8.360722

coinf <- subset(fig1, Samples=="W+/V+")
head(coinf)

## Country Species Samples GpSGHV Wolbachia Sodalis Wigglesworthia
## 1 South Africa G. austeni W+/V+ 3.071882 5.291369 5.226169 6.837400
## 2 South Africa G. austeni W+/V+ 3.631444 5.563006 2.453318 5.479647
## 3 South Africa G. austeni W+/V+ 3.070407 4.121888 3.376584 5.528074
## 4 South Africa G. austeni W+/V+ 3.071882 5.291369 5.138430 7.157453
## 8 South Africa G. austeni W+/V+ 3.071882 5.291369 5.240495 NA
## 12 South Africa G. austeni W+/V+ 3.467164 7.808886 3.083206 5.135913

summary(coinf)

## Country Species Samples GpSGHV
## Length:49 Length:49 Length:49 Min. : 2.496
## Class :character Class :character Class :character 1st Qu.: 2.939
## Mode :character Mode :character Mode :character Median : 3.394
## Mean : 3.867
## 3rd Qu.: 3.845
## Max. :11.321
##
## Wolbachia Sodalis Wigglesworthia
## Min. :3.202 Min. :2.453 Min. :4.943
## 1st Qu.:4.457 1st Qu.:3.651 1st Qu.:5.894
## Median :6.582 Median :5.089 Median :6.310
## Mean :6.292 Mean :4.970 Mean :6.933
## 3rd Qu.:7.709 3rd Qu.:6.361 3rd Qu.:8.642
## Max. :8.937 Max. :6.961 Max. :9.870
## NA's :9 NA's :1 NA's :8

coinf

## Country Species Samples GpSGHV Wolbachia Sodalis
## 1 South Africa G. austeni W+/V+ 3.071882 5.291369 5.226169
## 2 South Africa G. austeni W+/V+ 3.631444 5.563006 2.453318
## 3 South Africa G. austeni W+/V+ 3.070407 4.121888 3.376584
## 4 South Africa G. austeni W+/V+ 3.071882 5.291369 5.138430
## 8 South Africa G. austeni W+/V+ 3.071882 5.291369 5.240495
## 12 South Africa G. austeni W+/V+ 3.467164 7.808886 3.083206
## 13 South Africa G. austeni W+/V+ 3.098990 3.201943 3.387768
## 14 Tanzania G. austeni W+/V+ 3.735599 7.030195 3.340603
## 15 South Africa G. austeni W+/V+ 3.850033 6.418301 2.990989
## 16 South Africa G. austeni W+/V+ 3.152288 4.077731 3.357279
## 19 South Africa G. austeni W+/V+ 2.775246 3.959995 NA
## 20 South Africa G. austeni W+/V+ 2.857332 6.989450 6.789324
## 21 South Africa G. austeni W+/V+ 2.716003 NA 6.623851
## 22 South Africa G. austeni W+/V+ 2.775246 NA 2.567822
## 27 Zimbabwe G. m. morsitans W+/V+ 3.430720 4.483445 5.116050
## 28 Zimbabwe G. m. morsitans W+/V+ 2.938520 6.639486 4.256616
## 29 Zimbabwe G. m. morsitans W+/V+ 3.393751 8.936514 6.961294
## 33 Zambia G. m. morsitans W+/V+ 3.001734 8.466571 5.635447
## 34 Zimbabwe G. m. morsitans W+/V+ 3.385964 7.399328 5.896440
## 36 Zimbabwe G. m. morsitans W+/V+ 2.719331 7.360215 3.893365
## 39 Zimbabwe G. m. morsitans W+/V+ 11.320562 4.071882 6.256775
## 40 Zimbabwe G. m. morsitans W+/V+ 3.880814 6.806180 6.414824
## 41 Zimbabwe G. m. morsitans W+/V+ 3.880814 6.806180 6.518537
## 42 Zimbabwe G. m. morsitans W+/V+ 3.393751 8.936514 6.929733
## 44 Zimbabwe G. m. morsitans W+/V+ 11.320562 4.071882 6.342526
## 67 Zimbabwe G. m. morsitans W+/V+ 3.543571 3.989450 5.210836
## 69 Zimbabwe G. m. morsitans W+/V+ 3.393751 8.936514 6.948944
## 71 Zimbabwe G. m. morsitans W+/V+ 3.055378 6.523746 5.061756
## 75 Zimbabwe G. m. morsitans W+/V+ 3.055378 6.523746 4.963742
## 76 Zambia G. m. morsitans W+/V+ 2.496376 8.312177 3.718736
## 77 Zambia G. m. morsitans W+/V+ 2.496376 8.312177 4.014393
## 78 Zambia G. m. morsitans W+/V+ 2.496376 8.312177 4.016192
## 79 Zimbabwe G. m. morsitans W+/V+ 3.845098 7.709270 4.017656
## 80 Zimbabwe G. m. morsitans W+/V+ 2.719331 7.360215 4.150421
## 81 Zimbabwe G. m. morsitans W+/V+ 3.845098 7.709270 4.158713
## 83 Zimbabwe G. m. morsitans W+/V+ 4.112605 4.377306 3.264307
## 84 Zimbabwe G. m. morsitans W+/V+ 4.112605 4.377306 3.372475
## 85 Zimbabwe G. m. morsitans W+/V+ 4.112605 4.377306 3.446208
## 86 Zimbabwe G. m. morsitans W+/V+ 2.938520 6.639486 4.130066
## 90 Zimbabwe G. m. morsitans W+/V+ 3.845098 7.709270 4.133931
## 96 Zimbabwe G. m. morsitans W+/V+ 7.571476 NA 3.388551
## 97 Zimbabwe G. m. morsitans W+/V+ 3.857332 NA 6.626950
## 98 Zimbabwe G. m. morsitans W+/V+ 3.857332 NA 6.676298
## 99 Zimbabwe G. m. morsitans W+/V+ 11.320562 NA 6.333494
## 100 Zimbabwe G. m. morsitans W+/V+ 2.775246 NA 6.894753
## 101 Zimbabwe G. m. morsitans W+/V+ 2.775246 NA 6.904206
## 102 Zimbabwe G. m. morsitans W+/V+ 2.775246 NA 6.919518
## 117 Tanzania G. pallidipes W+/V+ 3.725912 5.745075 6.190086
## 118 Tanzania G. pallidipes W+/V+ 3.725912 5.745075 6.211020
## Wigglesworthia
## 1 6.837400
## 2 5.479647
## 3 5.528074
## 4 7.157453
## 8 NA
## 12 5.135913
## 13 5.497730
## 14 5.893921
## 15 6.963434
## 16 5.291318
## 19 NA
## 20 NA
## 21 NA
## 22 4.943486
## 27 6.310350
## 28 6.443300
## 29 6.116602
## 33 5.899101
## 34 5.952105
## 36 5.784220
## 39 9.388388
## 40 6.937702
## 41 6.805077
## 42 6.182330
## 44 9.297119
## 67 NA
## 69 NA
## 71 NA
## 75 6.663816
## 76 6.023311
## 77 6.096269
## 78 6.246218
## 79 6.575373
## 80 6.232853
## 81 6.097164
## 83 8.673505
## 84 8.657838
## 85 8.641584
## 86 6.787858
## 90 NA
## 96 5.400720
## 97 9.051741
## 98 9.070961
## 99 9.454929
## 100 9.808075
## 101 9.869617
## 102 9.788180
## 117 5.537838
## 118 5.731851

figs4a<-ggplot(coinf,aes(x=Species,y=Wolbachia, fill=Species)) +
 geom_boxplot() + geom_jitter(width=0.1,alpha=0.2)

tiff("figs4a.tiff", width = 4, height = 4, units = 'in', res = 300)
plot(figs4a+theme_tufte() + theme(axis.line = element_line(size = 1, colour = "black")) + theme(legend.title = element_text(face = "bold")) + theme(legend.text = element_text(face = "italic"))) + theme(axis.text.x = element_blank()) + xlab(expression(bold("Tsetse Species"))) + ylab(expression (paste (bold("log10 "), bolditalic("Wolbachia"), bold(" copy number"))))

## Warning: Removed 9 rows containing non-finite values (stat_boxplot).

## Warning: Removed 9 rows containing missing values (geom_point).

## Warning: Removed 9 rows containing non-finite values (stat_boxplot).

## Warning: Removed 9 rows containing missing values (geom_point).

dev.off()

## png
## 2

#------------------------------------------------------------------

figs4b<-ggplot(coinf,aes(x=Species,y=Sodalis, fill=Species)) +
 geom_boxplot() + geom_jitter(width=0.1,alpha=0.2)

tiff("figs4b.tiff", width = 4, height = 4, units = 'in', res = 300)
plot(figs4b+theme_tufte() + theme(axis.line = element_line(size = 1, colour = "black")) + theme(legend.title = element_text(face = "bold")) + theme(legend.text = element_text(face = "italic"))) + theme(axis.text.x = element_blank()) + xlab(expression(bold("Tsetse Species"))) + ylab(expression (paste (bold("log10 "), bolditalic("Sodalis"), bold(" copy number"))))

## Warning: Removed 1 rows containing non-finite values (stat_boxplot).

## Warning: Removed 1 rows containing missing values (geom_point).

## Warning: Removed 1 rows containing non-finite values (stat_boxplot).

## Warning: Removed 1 rows containing missing values (geom_point).

dev.off()

## png
## 2

#------------------------------------------------------------------

figs4c<-ggplot(coinf,aes(x=Species,y=Wigglesworthia, fill=Species)) +
 geom_boxplot() + geom_jitter(width=0.1,alpha=0.2)

tiff("figs4c.tiff", width = 4, height = 4, units = 'in', res = 300)
plot(figs4c+theme_tufte() + theme(axis.line = element_line(size = 1, colour = "black")) + theme(legend.title = element_text(face = "bold")) + theme(legend.text = element_text(face = "italic"))) + theme(axis.text.x = element_blank()) + xlab(expression(bold("Tsetse Species"))) + ylab(expression (paste (bold("log10 "), bolditalic("Wigglesworthia"), bold(" copy number"))))

## Warning: Removed 8 rows containing non-finite values (stat_boxplot).

## Warning: Removed 8 rows containing missing values (geom_point).

## Warning: Removed 8 rows containing non-finite values (stat_boxplot).

## Warning: Removed 8 rows containing missing values (geom_point).

dev.off()

## png
## 2

#------------------------------------------------------------------

figs4d<-ggplot(coinf,aes(x=Species,y=GpSGHV, fill=Species)) +
 geom_boxplot() + geom_jitter(width=0.1,alpha=0.2)

tiff("figs4d.tiff", width = 4, height = 4, units = 'in', res = 300)
plot(figs4d+theme_tufte() + theme(axis.line = element_line(size = 1, colour = "black")) + theme(legend.title = element_text(face = "bold")) + theme(legend.text = element_text(face = "italic"))) + theme(axis.text.x = element_blank()) + xlab(expression(bold("Tsetse Species"))) + ylab(expression (bold("log10 GpSGHV copy number ")))
dev.off()

## png
## 2

#------------------------------------------------------------
#Statistics for supplementary figure 4 (coinfection only (W+/V+))

coinf$Species=as.factor(coinf$Species)
coinf$Species <- relevel(coinf$Species, ref = "G. austeni")
gpsghv <- glm (GpSGHV~Species, data = coinf)
summary(gpsghv)

##
## Call:
## glm(formula = GpSGHV ~ Species, data = coinf)
##
## Deviance Residuals:
## Min 1Q Median 3Q Max
## -1.6754 -1.1164 -0.3266 -0.0591 7.1488
##
## Coefficients:
## Estimate Std. Error t value Pr(>|t|)
## (Intercept) 3.1675 0.5518 5.740 7.08e-07 ***
## SpeciesG. m. morsitans 1.0042 0.6585 1.525 0.134
## SpeciesG. pallidipes 0.5584 1.5607 0.358 0.722
## ---
## Signif. codes: 0 '***' 0.001 '**' 0.01 '*' 0.05 '.' 0.1 ' ' 1
##
## (Dispersion parameter for gaussian family taken to be 4.262741)
##
## Null deviance: 206.04 on 48 degrees of freedom
## Residual deviance: 196.09 on 46 degrees of freedom
## AIC: 215.01
##
## Number of Fisher Scoring iterations: 2

Anova(gpsghv)

## Analysis of Deviance Table (Type II tests)
##
## Response: GpSGHV
## LR Chisq Df Pr(>Chisq)
## Species 2.3351 2 0.3111

coinf$Species <- relevel(coinf$Species, ref = "G. m. morsitans")
gpsghv <- glm (GpSGHV~Species, data = coinf)
summary(gpsghv)

##
## Call:
## glm(formula = GpSGHV ~ Species, data = coinf)
##
## Deviance Residuals:
## Min 1Q Median 3Q Max
## -1.6754 -1.1164 -0.3266 -0.0591 7.1488
##
## Coefficients:
## Estimate Std. Error t value Pr(>|t|)
## (Intercept) 4.1717 0.3594 11.607 2.89e-15 ***
## SpeciesG. austeni -1.0042 0.6585 -1.525 0.134
## SpeciesG. pallidipes -0.4458 1.5035 -0.297 0.768
## ---
## Signif. codes: 0 '***' 0.001 '**' 0.01 '*' 0.05 '.' 0.1 ' ' 1
##
## (Dispersion parameter for gaussian family taken to be 4.262741)
##
## Null deviance: 206.04 on 48 degrees of freedom
## Residual deviance: 196.09 on 46 degrees of freedom
## AIC: 215.01
##
## Number of Fisher Scoring iterations: 2

coinf$Species <- relevel(coinf$Species, ref = "G. pallidipes")
gpsghv <- glm (GpSGHV~Species, data = coinf)
summary(gpsghv)

##
## Call:
## glm(formula = GpSGHV ~ Species, data = coinf)
##
## Deviance Residuals:
## Min 1Q Median 3Q Max
## -1.6754 -1.1164 -0.3266 -0.0591 7.1488
##
## Coefficients:
## Estimate Std. Error t value Pr(>|t|)
## (Intercept) 3.7259 1.4599 2.552 0.0141 *
## SpeciesG. m. morsitans 0.4458 1.5035 0.297 0.7682
## SpeciesG. austeni -0.5584 1.5607 -0.358 0.7222
## ---
## Signif. codes: 0 '***' 0.001 '**' 0.01 '*' 0.05 '.' 0.1 ' ' 1
##
## (Dispersion parameter for gaussian family taken to be 4.262741)
##
## Null deviance: 206.04 on 48 degrees of freedom
## Residual deviance: 196.09 on 46 degrees of freedom
## AIC: 215.01
##
## Number of Fisher Scoring iterations: 2

#-----------------------------------------------------------------
#wolbachia
coinf$Species=as.factor(coinf$Species)
coinf$Species <- relevel(coinf$Species, ref = "G. austeni")
wol <- glm (Wolbachia~Species, data = coinf)
summary(wol)

##
## Call:
## glm(formula = Wolbachia ~ Species, data = coinf)
##
## Deviance Residuals:
## Min 1Q Median 3Q Max
## -2.74700 -1.30961 0.03487 1.14063 2.38843
##
## Coefficients:
## Estimate Std. Error t value Pr(>|t|)
## (Intercept) 5.4205 0.4626 11.717 5.16e-14 ***
## SpeciesG. pallidipes 0.3246 1.2240 0.265 0.792
## SpeciesG. m. morsitans 1.3160 0.5593 2.353 0.024 *
## ---
## Signif. codes: 0 '***' 0.001 '**' 0.01 '*' 0.05 '.' 0.1 ' ' 1
##
## (Dispersion parameter for gaussian family taken to be 2.568236)
##
## Null deviance: 109.874 on 39 degrees of freedom
## Residual deviance: 95.025 on 37 degrees of freedom
## (9 observations deleted due to missingness)
## AIC: 156.13
##
## Number of Fisher Scoring iterations: 2

Anova(wol)

## Analysis of Deviance Table (Type II tests)
##
## Response: Wolbachia
## LR Chisq Df Pr(>Chisq)
## Species 5.7818 2 0.05553 .
## ---
## Signif. codes: 0 '***' 0.001 '**' 0.01 '*' 0.05 '.' 0.1 ' ' 1

coinf$Species <- relevel(coinf$Species, ref = "G. m. morsitans")
wol <- glm (Wolbachia~Species, data = coinf)
summary(wol)

##
## Call:
## glm(formula = Wolbachia ~ Species, data = coinf)
##
## Deviance Residuals:
## Min 1Q Median 3Q Max
## -2.74700 -1.30961 0.03487 1.14063 2.38843
##
## Coefficients:
## Estimate Std. Error t value Pr(>|t|)
## (Intercept) 6.7364 0.3143 21.434 <2e-16 ***
## SpeciesG. austeni -1.3160 0.5593 -2.353 0.024 *
## SpeciesG. pallidipes -0.9914 1.1760 -0.843 0.405
## ---
## Signif. codes: 0 '***' 0.001 '**' 0.01 '*' 0.05 '.' 0.1 ' ' 1
##
## (Dispersion parameter for gaussian family taken to be 2.568236)
##
## Null deviance: 109.874 on 39 degrees of freedom
## Residual deviance: 95.025 on 37 degrees of freedom
## (9 observations deleted due to missingness)
## AIC: 156.13
##
## Number of Fisher Scoring iterations: 2

coinf$Species <- relevel(coinf$Species, ref = "G. pallidipes")
wol <- glm (Wolbachia~Species, data = coinf)
summary(wol)

##
## Call:
## glm(formula = Wolbachia ~ Species, data = coinf)
##
## Deviance Residuals:
## Min 1Q Median 3Q Max
## -2.74700 -1.30961 0.03487 1.14063 2.38843
##
## Coefficients:
## Estimate Std. Error t value Pr(>|t|)
## (Intercept) 5.7451 1.1332 5.070 1.14e-05 ***
## SpeciesG. m. morsitans 0.9914 1.1760 0.843 0.405
## SpeciesG. austeni -0.3246 1.2240 -0.265 0.792
## ---
## Signif. codes: 0 '***' 0.001 '**' 0.01 '*' 0.05 '.' 0.1 ' ' 1
##
## (Dispersion parameter for gaussian family taken to be 2.568236)
##
## Null deviance: 109.874 on 39 degrees of freedom
## Residual deviance: 95.025 on 37 degrees of freedom
## (9 observations deleted due to missingness)
## AIC: 156.13
##
## Number of Fisher Scoring iterations: 2

#-----------------------------------------------------------------
#Wigglesworthia
coinf$Species=as.factor(coinf$Species)
coinf$Species <- relevel(coinf$Species, ref = "G. austeni")
wigg <- glm (Wigglesworthia~Species, data = coinf)
summary(wigg)

##
## Call:
## glm(formula = Wigglesworthia ~ Species, data = coinf)
##
## Deviance Residuals:
## Min 1Q Median 3Q Max
## -1.9874 -1.1419 -0.4505 1.2697 2.4815
##
## Coefficients:
## Estimate Std. Error t value Pr(>|t|)
## (Intercept) 5.8728 0.4351 13.499 4.46e-16 ***
## SpeciesG. pallidipes -0.2380 1.0657 -0.223 0.8245
## SpeciesG. m. morsitans 1.5153 0.5045 3.003 0.0047 **
## ---
## Signif. codes: 0 '***' 0.001 '**' 0.01 '*' 0.05 '.' 0.1 ' ' 1
##
## (Dispersion parameter for gaussian family taken to be 1.892801)
##
## Null deviance: 92.544 on 40 degrees of freedom
## Residual deviance: 71.926 on 38 degrees of freedom
## (8 observations deleted due to missingness)
## AIC: 147.4
##
## Number of Fisher Scoring iterations: 2

Anova(gpsghv)

## Analysis of Deviance Table (Type II tests)
##
## Response: GpSGHV
## LR Chisq Df Pr(>Chisq)
## Species 2.3351 2 0.3111

coinf$Species <- relevel(coinf$Species, ref = "G. m. morsitans")
wigg <- glm (Wigglesworthia~Species, data = coinf)
summary(wigg)

##
## Call:
## glm(formula = Wigglesworthia ~ Species, data = coinf)
##
## Deviance Residuals:
## Min 1Q Median 3Q Max
## -1.9874 -1.1419 -0.4505 1.2697 2.4815
##
## Coefficients:
## Estimate Std. Error t value Pr(>|t|)
## (Intercept) 7.3881 0.2555 28.919 <2e-16 ***
## SpeciesG. austeni -1.5153 0.5045 -3.003 0.0047 **
## SpeciesG. pallidipes -1.7533 1.0058 -1.743 0.0894 .
## ---
## Signif. codes: 0 '***' 0.001 '**' 0.01 '*' 0.05 '.' 0.1 ' ' 1
##
## (Dispersion parameter for gaussian family taken to be 1.892801)
##
## Null deviance: 92.544 on 40 degrees of freedom
## Residual deviance: 71.926 on 38 degrees of freedom
## (8 observations deleted due to missingness)
## AIC: 147.4
##
## Number of Fisher Scoring iterations: 2

coinf$Species <- relevel(coinf$Species, ref = "G. pallidipes")
wigg <- glm (Wigglesworthia~Species, data = coinf)
summary(wigg)

##
## Call:
## glm(formula = Wigglesworthia ~ Species, data = coinf)
##
## Deviance Residuals:
## Min 1Q Median 3Q Max
## -1.9874 -1.1419 -0.4505 1.2697 2.4815
##
## Coefficients:
## Estimate Std. Error t value Pr(>|t|)
## (Intercept) 5.6348 0.9728 5.792 1.1e-06 ***
## SpeciesG. m. morsitans 1.7533 1.0058 1.743 0.0894 .
## SpeciesG. austeni 0.2380 1.0657 0.223 0.8245
## ---
## Signif. codes: 0 '***' 0.001 '**' 0.01 '*' 0.05 '.' 0.1 ' ' 1
##
## (Dispersion parameter for gaussian family taken to be 1.892801)
##
## Null deviance: 92.544 on 40 degrees of freedom
## Residual deviance: 71.926 on 38 degrees of freedom
## (8 observations deleted due to missingness)
## AIC: 147.4
##
## Number of Fisher Scoring iterations: 2

#------------------------------------------------------------------

#Sodalis
coinf$Species=as.factor(coinf$Species)
coinf$Species <- relevel(coinf$Species, ref = "G. austeni")
sod <- glm (Sodalis~Species, data = coinf)
summary(sod)

##
## Call:
## glm(formula = Sodalis ~ Species, data = coinf)
##
## Deviance Residuals:
## Min 1Q Median 3Q Max
## -1.9652 -1.1071 -0.1406 1.1358 2.6681
##
## Coefficients:
## Estimate Std. Error t value Pr(>|t|)
## (Intercept) 4.1212 0.3775 10.916 3.11e-14 ***
## SpeciesG. pallidipes 2.0793 1.0339 2.011 0.0503 .
## SpeciesG. m. morsitans 1.1083 0.4457 2.486 0.0167 *
## ---
## Signif. codes: 0 '***' 0.001 '**' 0.01 '*' 0.05 '.' 0.1 ' ' 1
##
## (Dispersion parameter for gaussian family taken to be 1.852986)
##
## Null deviance: 98.001 on 47 degrees of freedom
## Residual deviance: 83.384 on 45 degrees of freedom
## (1 observation deleted due to missingness)
## AIC: 170.73
##
## Number of Fisher Scoring iterations: 2

Anova(sod)

## Analysis of Deviance Table (Type II tests)
##
## Response: Sodalis
## LR Chisq Df Pr(>Chisq)
## Species 7.8881 2 0.01937 *
## ---
## Signif. codes: 0 '***' 0.001 '**' 0.01 '*' 0.05 '.' 0.1 ' ' 1

coinf$Species <- relevel(coinf$Species, ref = "G. m. morsitans")
sod <- glm (Sodalis~Species, data = coinf)
summary(sod)

##
## Call:
## glm(formula = Sodalis ~ Species, data = coinf)
##
## Deviance Residuals:
## Min 1Q Median 3Q Max
## -1.9652 -1.1071 -0.1406 1.1358 2.6681
##
## Coefficients:
## Estimate Std. Error t value Pr(>|t|)
## (Intercept) 5.2295 0.2370 22.069 <2e-16 ***
## SpeciesG. austeni -1.1083 0.4457 -2.486 0.0167 *
## SpeciesG. pallidipes 0.9710 0.9913 0.980 0.3325
## ---
## Signif. codes: 0 '***' 0.001 '**' 0.01 '*' 0.05 '.' 0.1 ' ' 1
##
## (Dispersion parameter for gaussian family taken to be 1.852986)
##
## Null deviance: 98.001 on 47 degrees of freedom
## Residual deviance: 83.384 on 45 degrees of freedom
## (1 observation deleted due to missingness)
## AIC: 170.73
##
## Number of Fisher Scoring iterations: 2

coinf$Species <- relevel(coinf$Species, ref = "G. pallidipes")
sod <- glm (Wolbachia~Species, data = coinf)
summary(sod)

##
## Call:
## glm(formula = Wolbachia ~ Species, data = coinf)
##
## Deviance Residuals:
## Min 1Q Median 3Q Max
## -2.74700 -1.30961 0.03487 1.14063 2.38843
##
## Coefficients:
## Estimate Std. Error t value Pr(>|t|)
## (Intercept) 5.7451 1.1332 5.070 1.14e-05 ***
## SpeciesG. m. morsitans 0.9914 1.1760 0.843 0.405
## SpeciesG. austeni -0.3246 1.2240 -0.265 0.792
## ---
## Signif. codes: 0 '***' 0.001 '**' 0.01 '*' 0.05 '.' 0.1 ' ' 1
##
## (Dispersion parameter for gaussian family taken to be 2.568236)
##
## Null deviance: 109.874 on 39 degrees of freedom
## Residual deviance: 95.025 on 37 degrees of freedom
## (9 observations deleted due to missingness)
## AIC: 156.13
##
## Number of Fisher Scoring iterations: 2

## Preparing Figure 2 and its statistics

fig2 <- read.csv("virus_symb_all_20210527_2m2_gmm_ga_2prime.csv")
fig2=na.omit(fig2)
fig2

## ID Country Species Sample GpSGHV Wolbachia Sodalis
## 2 2 South Africa G. austeni W+/V+ 3.071882 5.291369 5.226169
## 3 3 South Africa G. austeni W+/V+ 3.631444 5.563006 2.453318
## 4 4 South Africa G. austeni W+/V+ 3.070407 4.121888 3.376584
## 5 5 South Africa G. austeni W+/V+ 3.071882 5.291369 5.138430
## 6 6 South Africa G. austeni W+/V- 6.330414 6.542078 5.056997
## 7 7 South Africa G. austeni W+/V- 6.322219 6.544068 5.070590
## 13 13 South Africa G. austeni W+/V- 2.606381 6.670246 3.527706
## 14 14 South Africa G. austeni W+/V+ 3.467164 7.808886 3.083206
## 15 15 South Africa G. austeni W+/V+ 3.098990 3.201943 3.387768
## 16 16 South Africa G. austeni W+/V+ 3.850033 6.418301 2.990989
## 17 17 South Africa G. austeni W+/V+ 3.152288 4.077731 3.357279
## 22 22 Tanzania G. austeni W+/V+ 3.735599 7.030195 3.340603
## 23 23 Tanzania G. m. morsitans W+/V- 6.561578 8.147367 5.716086
## 24 24 Tanzania G. m. morsitans W+/V- 3.356408 8.372175 5.854843
## 25 25 Tanzania G. m. morsitans W+/V- 3.304491 8.268578 6.038572
## 41 41 Zambia G. m. morsitans W+/V- 3.695482 11.193125 6.979752
## 42 42 Zambia G. m. morsitans W+/V+ 3.001734 8.466571 5.635447
## 48 48 Zambia G. m. morsitans W+/V+ 2.496376 8.312177 3.718736
## 49 49 Zambia G. m. morsitans W+/V+ 2.496376 8.312177 4.014393
## 50 50 Zambia G. m. morsitans W+/V+ 2.496376 8.312177 4.016192
## 51 51 Zambia G. m. morsitans W+/V- 2.124504 15.658965 5.505514
## 52 52 Zambia G. m. morsitans W+/V- 2.121888 8.158362 6.220152
## 56 56 Zimbabwe G. m. morsitans W+/V- 4.331225 6.991226 4.705386
## 57 57 Zimbabwe G. m. morsitans W+/V+ 3.430720 4.483445 5.116050
## 58 58 Zimbabwe G. m. morsitans W+/V+ 3.385964 7.399328 5.896440
## 61 61 Zimbabwe G. m. morsitans W+/V- 3.982271 6.248464 3.628272
## 65 65 Zimbabwe G. m. morsitans W+/V+ 2.938520 6.639486 4.256616
## 66 66 Zimbabwe G. m. morsitans W+/V+ 3.393751 8.936514 6.961294
## 67 67 Zimbabwe G. m. morsitans W+/V- 2.878522 7.510009 7.576463
## 68 68 Zimbabwe G. m. morsitans W+/V- 3.186391 7.230449 3.827807
## 69 69 Zimbabwe G. m. morsitans W+/V+ 2.719331 7.360215 3.893365
## 70 70 Zimbabwe G. m. morsitans W+/V- 3.108565 7.845098 4.668694
## 71 71 Zimbabwe G. m. morsitans W-/V+ 3.748188 4.705864 6.200526
## 72 72 Zimbabwe G. m. morsitans W+/V+ 11.320562 4.071882 6.256775
## 73 73 Zimbabwe G. m. morsitans W+/V+ 3.880814 6.806180 6.414824
## 74 74 Zimbabwe G. m. morsitans W+/V+ 3.880814 6.806180 6.518537
## 75 75 Zimbabwe G. m. morsitans W+/V+ 3.393751 8.936514 6.929733
## 76 76 Zimbabwe G. m. morsitans W+/V+ 11.320562 4.071882 6.342526
## 81 81 Zimbabwe G. m. morsitans W+/V- 2.896526 7.261976 4.395513
## 82 82 Zimbabwe G. m. morsitans W+/V+ 3.055378 6.523746 4.963742
## 83 83 Zimbabwe G. m. morsitans W+/V+ 3.845098 7.709270 4.017656
## 84 84 Zimbabwe G. m. morsitans W+/V+ 2.719331 7.360215 4.150421
## 85 85 Zimbabwe G. m. morsitans W+/V+ 3.845098 7.709270 4.158713
## 86 86 Zimbabwe G. m. morsitans W+/V- 2.857332 6.900913 4.304863
## 87 87 Zimbabwe G. m. morsitans W+/V+ 2.938520 6.639486 4.130066
## 88 88 Zimbabwe G. m. morsitans W+/V- 3.031812 9.655138 4.228599
## 95 95 Zimbabwe G. m. morsitans W+/V- 4.264818 3.889862 6.201792
## 96 96 Zimbabwe G. m. morsitans W+/V- 6.001734 3.985875 4.535826
## 97 97 Zimbabwe G. m. morsitans W+/V+ 4.112605 4.377306 3.264307
## 98 98 Zimbabwe G. m. morsitans W+/V+ 4.112605 4.377306 3.372475
## 99 99 Zimbabwe G. m. morsitans W+/V+ 4.112605 4.377306 3.446208
## 100 100 Zimbabwe G. m. morsitans W+/V- 4.176091 4.449633 4.999776
## Wigglesworthia
## 2 6.837400
## 3 5.479647
## 4 5.528074
## 5 7.157453
## 6 6.218625
## 7 6.208117
## 13 5.733434
## 14 5.135913
## 15 5.497730
## 16 6.963434
## 17 5.291318
## 22 5.893921
## 23 5.459354
## 24 7.098055
## 25 7.124997
## 41 6.678722
## 42 5.899101
## 48 6.023311
## 49 6.096269
## 50 6.246218
## 51 6.393184
## 52 6.094564
## 56 5.339160
## 57 6.310350
## 58 5.952105
## 61 5.789754
## 65 6.443300
## 66 6.116602
## 67 6.804350
## 68 6.344745
## 69 5.784220
## 70 7.800503
## 71 10.062864
## 72 9.388388
## 73 6.937702
## 74 6.805077
## 75 6.182330
## 76 9.297119
## 81 6.327612
## 82 6.663816
## 83 6.575373
## 84 6.232853
## 85 6.097164
## 86 7.148731
## 87 6.787858
## 88 6.356982
## 95 10.191603
## 96 9.838294
## 97 8.673505
## 98 8.657838
## 99 8.641584
## 100 10.067865

fig2a<-ggplot(fig2, aes(Wolbachia, GpSGHV, col = Species, fill = Species)) +
 geom_point(size = 3, shape = 21, col = "black") +
 geom_vline(xintercept = 9, color = "black", size=1)+
 xlab(expression(italic("Wolbachia")))
fig2a


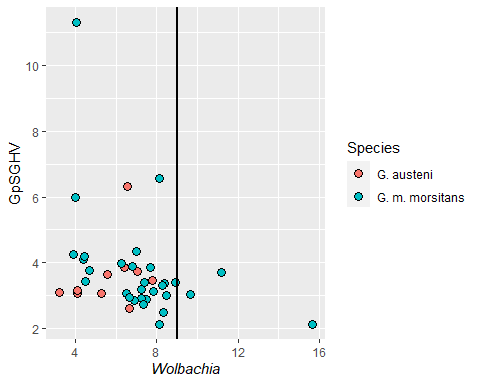


tiff("fig2a.tiff", width = 4, height = 4, units = 'in', res = 300)
plot(fig2a+theme_tufte()+ theme(axis.line = element_line(size = 1, colour = "black")) + theme(legend.title = element_text(face = "bold")) + theme(legend.text = element_text(face = "italic"))) + xlab(expression(bolditalic("Wolbachia"))) + ylab(expression(bold("GpSGHV")))
dev.off()

## png
## 2

#----------------------------------------------------------

fig2b<-ggplot(fig2, aes(Sodalis, GpSGHV, col = Species, fill = Species)) +
 geom_point(size = 3, shape = 21, col = "black") +
 xlab(expression(italic("Sodalis")))
fig2b


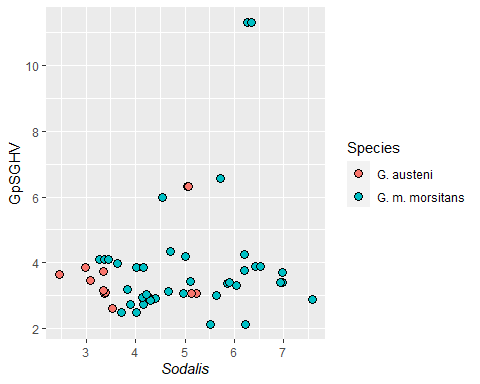


tiff("fig2b2.tiff", width = 4, height = 4, units = 'in', res = 300)
plot(fig2b+theme_tufte() + theme(axis.line = element_line(size = 1, colour = "black")) + theme(legend.title = element_text(face = "bold")) + theme(legend.text = element_text(face = "italic")))+ xlab(expression(bolditalic("Sodalis"))) + ylab(expression(bold("GpSGHV")))
dev.off()

## png
## 2

#----------------------------------------------------------
fig2c<-ggplot(fig2, aes(Wigglesworthia, GpSGHV, col = Species, fill = Species)) +
 geom_point(size = 3, shape = 21, col = "black") +
 xlab(expression(italic("Wigglesworthia")))
fig2c


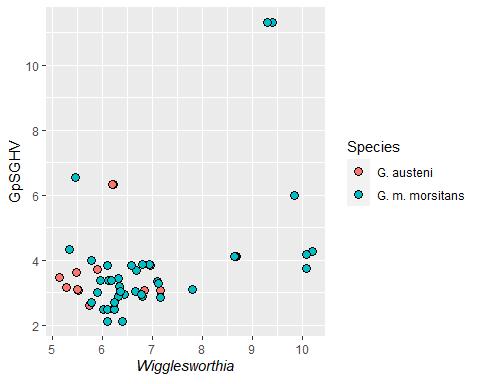


tiff("fig2c.tiff", width = 4, height = 4, units = 'in', res = 300)
plot(fig2c+theme_tufte() + theme(axis.line = element_line(size = 1, colour = "black")) + theme(legend.title = element_text(face = "bold")) + theme(legend.text = element_text(face = "italic")))+ xlab(expression(bolditalic("Wigglesworthia"))) + ylab(expression(bold("GpSGHV")))
dev.off()

## png
## 2

#----------------------------------------------------------

fig2d<-ggplot(fig2, aes(Wigglesworthia, Sodalis, col = Species, fill = Species)) +
 geom_point(size = 3, shape = 21, col = "black") +
 xlab(expression(italic("Wigglesworthia")))
fig2d


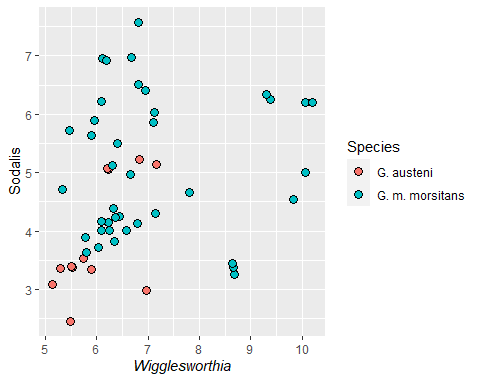


tiff("fig2d.tiff", width = 4, height = 4, units = 'in', res = 300)
plot(fig2d+theme_tufte() + theme(axis.line = element_line(size = 1, colour = "black")) + theme(legend.title = element_text(face = "bold")) + theme(legend.text = element_text(face = "italic"))) + xlab(expression(bolditalic("Wigglesworthia"))) + ylab(expression(bolditalic("Sodalis")))
dev.off()

## png
## 2

#----------------------------------------------------------
fig2e<-ggplot(fig2, aes(Wolbachia, Sodalis, col = Species, fill = Species)) +
 geom_point(size = 3, shape = 21, col = "black") +
 xlab(expression(italic("Wolbachia")))
fig2e


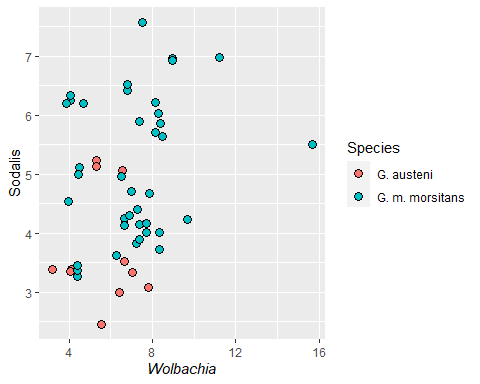


tiff("fig2e.tiff", width = 4, height = 4, units = 'in', res = 300)
plot(fig2e+theme_tufte() + theme(axis.line = element_line(size = 1, colour = "black")) + theme(legend.title = element_text(face = "bold")) + theme(legend.text = element_text(face = "italic"))) + xlab(expression(bolditalic("Wolbachia"))) + ylab(expression(bolditalic("Sodalis")))
dev.off()

## png
## 2

#----------------------------------------------------------

fig2f<-ggplot(fig2, aes(Wolbachia, Wigglesworthia, col = Species, fill = Species)) +
 geom_point(size = 3, shape = 21, col = "black") +
 geom_vline(xintercept = 8.2, color = "black", size=1)+
 xlab(expression(italic("Wolbachia")))
fig2f


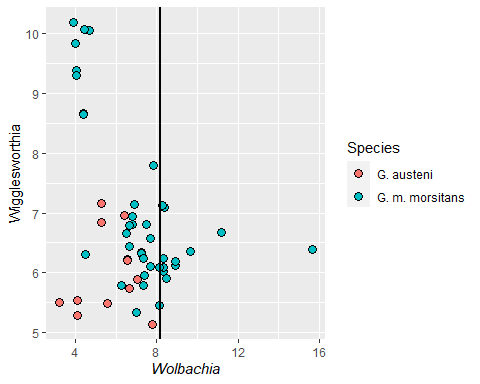


tiff("fig2f2.tiff", width = 4, height = 4, units = 'in', res = 300)
plot(fig2f+theme_tufte() + theme(axis.line = element_line(size = 1, colour = "black")) + theme(legend.title = element_text(face = "bold")) + theme(legend.text = element_text(face = "italic"))) + xlab(expression(bolditalic("Wolbachia"))) + ylab(expression(bolditalic("Wigglesworthia")))
dev.off()

## png
## 2

#Wolbachia~Sodalis (positive = 0.2595844)
cor.test(fig2$Wolbachia, log(fig2$Sodalis), method = "pearson")

##
## Pearson's product-moment correlation
##
## data: fig2$Wolbachia and log(fig2$Sodalis)
## t = 1.9469, df = 50, p-value = 0.05718
## alternative hypothesis: true correlation is not equal to 0
## 95 percent confidence interval:
## -0.008029974 0.501987423
## sample estimates:
## cor
## 0.2654521

#Wolbachia~virus (negative = -0.3705659)
cor.test(fig2$Wolbachia, log(fig2$GpSGHV), method = "pearson")

##
## Pearson's product-moment correlation
##
## data: fig2$Wolbachia and log(fig2$GpSGHV)
## t = -3.392, df = 50, p-value = 0.001364
## alternative hypothesis: true correlation is not equal to 0
## 95 percent confidence interval:
## -0.6309434 -0.1809766
## sample estimates:
## cor
## -0.4325156

#Wolbachia~Wigglesworthia (negative = -0.4280967 )
cor.test(fig2$Wolbachia, fig2$Wigglesworthia, method = "pearson")

##
## Pearson's product-moment correlation
##
## data: fig2$Wolbachia and fig2$Wigglesworthia
## t = -3.3496, df = 50, p-value = 0.001546
## alternative hypothesis: true correlation is not equal to 0
## 95 percent confidence interval:
## -0.6276680 -0.1757261
## sample estimates:
## cor
## -0.4280967

#virus_Wiggelsworthis (positive = 0.4431483)
cor.test(log(fig2$GpSGHV), fig2$Wigglesworthia, method = "pearson")

##
## Pearson's product-moment correlation
##
## data: log(fig2$GpSGHV) and fig2$Wigglesworthia
## t = 3.6087, df = 50, p-value = 0.0007108
## alternative hypothesis: true correlation is not equal to 0
## 95 percent confidence interval:
## 0.2073993 0.6471864
## sample estimates:
## cor
## 0.4545712

#Virus~Sodalis (positive = 0.2401435 )
cor.test(log(fig2$GpSGHV), log(fig2$Sodalis), method = "pearson")

##
## Pearson's product-moment correlation
##
## data: log(fig2$GpSGHV) and log(fig2$Sodalis)
## t = 1.4339, df = 50, p-value = 0.1578
## alternative hypothesis: true correlation is not equal to 0
## 95 percent confidence interval:
## -0.07841395 0.44737568
## sample estimates:
## cor
## 0.1987392

#Sodalis~Wigglesworthis (positive = 0.2575499)
cor.test(log(fig2$Sodalis), fig2$Wigglesworthia, method = "pearson")

##
## Pearson's product-moment correlation
##
## data: log(fig2$Sodalis) and fig2$Wigglesworthia
## t = 1.9647, df = 50, p-value = 0.05502
## alternative hypothesis: true correlation is not equal to 0
## 95 percent confidence interval:
## -0.005605601 0.503798752
## sample estimates:
## cor
## 0.2677042

fig2_ga <- subset(fig2, Species=="G. austeni")
fig2_ga

## ID Country Species Sample GpSGHV Wolbachia Sodalis Wigglesworthia
## 2 2 South Africa G. austeni W+/V+ 3.071882 5.291369 5.226169 6.837400
## 3 3 South Africa G. austeni W+/V+ 3.631444 5.563006 2.453318 5.479647
## 4 4 South Africa G. austeni W+/V+ 3.070407 4.121888 3.376584 5.528074
## 5 5 South Africa G. austeni W+/V+ 3.071882 5.291369 5.138430 7.157453
## 6 6 South Africa G. austeni W+/V- 6.330414 6.542078 5.056997 6.218625
## 7 7 South Africa G. austeni W+/V- 6.322219 6.544068 5.070590 6.208117
## 13 13 South Africa G. austeni W+/V- 2.606381 6.670246 3.527706 5.733434
## 14 14 South Africa G. austeni W+/V+ 3.467164 7.808886 3.083206 5.135913
## 15 15 South Africa G. austeni W+/V+ 3.098990 3.201943 3.387768 5.497730
## 16 16 South Africa G. austeni W+/V+ 3.850033 6.418301 2.990989 6.963434
## 17 17 South Africa G. austeni W+/V+ 3.152288 4.077731 3.357279 5.291318
## 22 22 Tanzania G. austeni W+/V+ 3.735599 7.030195 3.340603 5.893921

#Wolbachia~virus (negative = -0.3705659)
cor.test(fig2_ga$Wolbachia, log(fig2_ga$GpSGHV), method = "pearson")

##
## Pearson's product-moment correlation
##
## data: fig2_ga$Wolbachia and log(fig2_ga$GpSGHV)
## t = 1.3122, df = 10, p-value = 0.2188
## alternative hypothesis: true correlation is not equal to 0
## 95 percent confidence interval:
## -0.2443904 0.7845916
## sample estimates:
## cor
## 0.3832667

#virus_Wiggelsworthis (positive = 0.4431483)
cor.test(log(fig2_ga$GpSGHV), fig2_ga$Wigglesworthia, method = "pearson")

##
## Pearson's product-moment correlation
##
## data: log(fig2_ga$GpSGHV) and fig2_ga$Wigglesworthia
## t = 0.52401, df = 10, p-value = 0.6117
## alternative hypothesis: true correlation is not equal to 0
## 95 percent confidence interval:
## -0.4529178 0.6741314
## sample estimates:
## cor
## 0.1634763

#Virus~Sodalis (positive = 0.2401435 )
cor.test(log(fig2_ga$GpSGHV), log(fig2_ga$Sodalis), method = "pearson")

##
## Pearson's product-moment correlation
##
## data: log(fig2_ga$GpSGHV) and log(fig2_ga$Sodalis)
## t = 1.2505, df = 10, p-value = 0.2396
## alternative hypothesis: true correlation is not equal to 0
## 95 percent confidence interval:
## -0.2613164 0.7775411
## sample estimates:
## cor
## 0.3677343

#Wolbachia~Wigglesworthia (negative = -0.4280967 )
cor.test(fig2_ga$Wolbachia, fig2_ga$Wigglesworthia, method = "pearson")

##
## Pearson's product-moment correlation
##
## data: fig2_ga$Wolbachia and fig2_ga$Wigglesworthia
## t = 0.38071, df = 10, p-value = 0.7114
## alternative hypothesis: true correlation is not equal to 0
## 95 percent confidence interval:
## -0.4878384 0.6489155
## sample estimates:
## cor
## 0.1195275

#Wolbachia~Sodalis (positive = 0.2595844)
cor.test(fig2_ga$Wolbachia, log(fig2_ga$Sodalis), method = "pearson")

##
## Pearson's product-moment correlation
##
## data: fig2_ga$Wolbachia and log(fig2_ga$Sodalis)
## t = 0.092087, df = 10, p-value = 0.9284
## alternative hypothesis: true correlation is not equal to 0
## 95 percent confidence interval:
## -0.5540490 0.5931018
## sample estimates:
## cor
## 0.02910811

#Sodalis~Wigglesworthis (positive = 0.2575499)
cor.test(log(fig2_ga$Sodalis), fig2_ga$Wigglesworthia, method = "pearson")

##
## Pearson's product-moment correlation
##
## data: log(fig2_ga$Sodalis) and fig2_ga$Wigglesworthia
## t = 2.3865, df = 10, p-value = 0.03819
## alternative hypothesis: true correlation is not equal to 0
## 95 percent confidence interval:
## 0.04352898 0.87410029
## sample estimates:
## cor
## 0.6023823

fig2_gmm <- subset(fig2, Species=="G. m. morsitans")
fig2_gmm

## ID Country Species Sample GpSGHV Wolbachia Sodalis
## 23 23 Tanzania G. m. morsitans W+/V- 6.561578 8.147367 5.716086
## 24 24 Tanzania G. m. morsitans W+/V- 3.356408 8.372175 5.854843
## 25 25 Tanzania G. m. morsitans W+/V- 3.304491 8.268578 6.038572
## 41 41 Zambia G. m. morsitans W+/V- 3.695482 11.193125 6.979752
## 42 42 Zambia G. m. morsitans W+/V+ 3.001734 8.466571 5.635447
## 48 48 Zambia G. m. morsitans W+/V+ 2.496376 8.312177 3.718736
## 49 49 Zambia G. m. morsitans W+/V+ 2.496376 8.312177 4.014393
## 50 50 Zambia G. m. morsitans W+/V+ 2.496376 8.312177 4.016192
## 51 51 Zambia G. m. morsitans W+/V- 2.124504 15.658965 5.505514
## 52 52 Zambia G. m. morsitans W+/V- 2.121888 8.158362 6.220152
## 56 56 Zimbabwe G. m. morsitans W+/V- 4.331225 6.991226 4.705386
## 57 57 Zimbabwe G. m. morsitans W+/V+ 3.430720 4.483445 5.116050
## 58 58 Zimbabwe G. m. morsitans W+/V+ 3.385964 7.399328 5.896440
## 61 61 Zimbabwe G. m. morsitans W+/V- 3.982271 6.248464 3.628272
## 65 65 Zimbabwe G. m. morsitans W+/V+ 2.938520 6.639486 4.256616
## 66 66 Zimbabwe G. m. morsitans W+/V+ 3.393751 8.936514 6.961294
## 67 67 Zimbabwe G. m. morsitans W+/V- 2.878522 7.510009 7.576463
## 68 68 Zimbabwe G. m. morsitans W+/V- 3.186391 7.230449 3.827807
## 69 69 Zimbabwe G. m. morsitans W+/V+ 2.719331 7.360215 3.893365
## 70 70 Zimbabwe G. m. morsitans W+/V- 3.108565 7.845098 4.668694
## 71 71 Zimbabwe G. m. morsitans W-/V+ 3.748188 4.705864 6.200526
## 72 72 Zimbabwe G. m. morsitans W+/V+ 11.320562 4.071882 6.256775
## 73 73 Zimbabwe G. m. morsitans W+/V+ 3.880814 6.806180 6.414824
## 74 74 Zimbabwe G. m. morsitans W+/V+ 3.880814 6.806180 6.518537
## 75 75 Zimbabwe G. m. morsitans W+/V+ 3.393751 8.936514 6.929733
## 76 76 Zimbabwe G. m. morsitans W+/V+ 11.320562 4.071882 6.342526
## 81 81 Zimbabwe G. m. morsitans W+/V- 2.896526 7.261976 4.395513
## 82 82 Zimbabwe G. m. morsitans W+/V+ 3.055378 6.523746 4.963742
## 83 83 Zimbabwe G. m. morsitans W+/V+ 3.845098 7.709270 4.017656
## 84 84 Zimbabwe G. m. morsitans W+/V+ 2.719331 7.360215 4.150421
## 85 85 Zimbabwe G. m. morsitans W+/V+ 3.845098 7.709270 4.158713
## 86 86 Zimbabwe G. m. morsitans W+/V- 2.857332 6.900913 4.304863
## 87 87 Zimbabwe G. m. morsitans W+/V+ 2.938520 6.639486 4.130066
## 88 88 Zimbabwe G. m. morsitans W+/V- 3.031812 9.655138 4.228599
## 95 95 Zimbabwe G. m. morsitans W+/V- 4.264818 3.889862 6.201792
## 96 96 Zimbabwe G. m. morsitans W+/V- 6.001734 3.985875 4.535826
## 97 97 Zimbabwe G. m. morsitans W+/V+ 4.112605 4.377306 3.264307
## 98 98 Zimbabwe G. m. morsitans W+/V+ 4.112605 4.377306 3.372475
## 99 99 Zimbabwe G. m. morsitans W+/V+ 4.112605 4.377306 3.446208
## 100 100 Zimbabwe G. m. morsitans W+/V- 4.176091 4.449633 4.999776
## Wigglesworthia
## 23 5.459354
## 24 7.098055
## 25 7.124997
## 41 6.678722
## 42 5.899101
## 48 6.023311
## 49 6.096269
## 50 6.246218
## 51 6.393184
## 52 6.094564
## 56 5.339160
## 57 6.310350
## 58 5.952105
## 61 5.789754
## 65 6.443300
## 66 6.116602
## 67 6.804350
## 68 6.344745
## 69 5.784220
## 70 7.800503
## 71 10.062864
## 72 9.388388
## 73 6.937702
## 74 6.805077
## 75 6.182330
## 76 9.297119
## 81 6.327612
## 82 6.663816
## 83 6.575373
## 84 6.232853
## 85 6.097164
## 86 7.148731
## 87 6.787858
## 88 6.356982
## 95 10.191603
## 96 9.838294
## 97 8.673505
## 98 8.657838
## 99 8.641584
## 100 10.067865

#Wolbachia~virus (negative = -0.3705659)
cor.test(fig2_gmm$Wolbachia, log(fig2_gmm$GpSGHV), method = "pearson")

##
## Pearson's product-moment correlation
##
## data: fig2_gmm$Wolbachia and log(fig2_gmm$GpSGHV)
## t = -4.1505, df = 38, p-value = 0.0001802
## alternative hypothesis: true correlation is not equal to 0
## 95 percent confidence interval:
## -0.7410802 -0.2990164
## sample estimates:
## cor
## -0.5585032

#virus_Wiggelsworthis (positive = 0.4431483)
cor.test(log(fig2_gmm$GpSGHV), fig2_gmm$Wigglesworthia, method = "pearson")

##
## Pearson's product-moment correlation
##
## data: log(fig2_gmm$GpSGHV) and fig2_gmm$Wigglesworthia
## t = 3.8686, df = 38, p-value = 0.0004162
## alternative hypothesis: true correlation is not equal to 0
## 95 percent confidence interval:
## 0.2637253 0.7233043
## sample estimates:
## cor
## 0.531565

#Virus~Sodalis (positive = 0.2401435 )
cor.test(log(fig2_gmm$GpSGHV), log(fig2_gmm$Sodalis), method = "pearson")

##
## Pearson's product-moment correlation
##
## data: log(fig2_gmm$GpSGHV) and log(fig2_gmm$Sodalis)
## t = 1.2759, df = 38, p-value = 0.2097
## alternative hypothesis: true correlation is not equal to 0
## 95 percent confidence interval:
## -0.1161646 0.4836522
## sample estimates:
## cor
## 0.2026789

#Wolbachia~Wigglesworthia (negative = -0.4280967 )
cor.test(fig2_gmm$Wolbachia, fig2_gmm$Wigglesworthia)

##
## Pearson's product-moment correlation
##
## data: fig2_gmm$Wolbachia and fig2_gmm$Wigglesworthia
## t = -5.0948, df = 38, p-value = 9.87e-06
## alternative hypothesis: true correlation is not equal to 0
## 95 percent confidence interval:
## -0.7914991 -0.4061568
## sample estimates:
## cor
## -0.6370638

#Wolbachia~Sodalis (positive = 0.2595844)
cor.test(fig2_gmm$Wolbachia, log(fig2_gmm$Sodalis), method = "pearson")

##
## Pearson's product-moment correlation
##
## data: fig2_gmm$Wolbachia and log(fig2_gmm$Sodalis)
## t = 1.2136, df = 38, p-value = 0.2324
## alternative hypothesis: true correlation is not equal to 0
## 95 percent confidence interval:
## -0.1259167 0.4760340
## sample estimates:
## cor
## 0.1931696

#Sodalis~Wigglesworthis (positive = 0.2575499)
cor.test(log(fig2_gmm$Sodalis), fig2_gmm$Wigglesworthia, method = "pearson")

##
## Pearson's product-moment correlation
##
## data: log(fig2_gmm$Sodalis) and fig2_gmm$Wigglesworthia
## t = 0.44342, df = 38, p-value = 0.66
## alternative hypothesis: true correlation is not equal to 0
## 95 percent confidence interval:
## -0.2452439 0.3748775
## sample estimates:
## cor
## 0.07174661

#------------------------------------------------------


wol <- glm (Wolbachia~Species, data = fig2)
summary(wol)

##
## Call:
## glm(formula = Wolbachia ~ Species, data = fig2)
##
## Deviance Residuals:
## Min 1Q Median 3Q Max
## -3.2217 -1.0452 0.1996 1.0744 8.5474
##
## Coefficients:
## Estimate Std. Error t value Pr(>|t|)
## (Intercept) 5.7134 0.6044 9.453 1.03e-12 ***
## SpeciesG. m. morsitans 1.3981 0.6891 2.029 0.0478 *
## ---
## Signif. codes: 0 '***' 0.001 '**' 0.01 '*' 0.05 '.' 0.1 ' ' 1
##
## (Dispersion parameter for gaussian family taken to be 4.383742)
##
## Null deviance: 237.23 on 51 degrees of freedom
## Residual deviance: 219.19 on 50 degrees of freedom
## AIC: 228.38
##
## Number of Fisher Scoring iterations: 2

wigg <- glm (Wigglesworthia~Species, data = fig2)
summary(wigg)

##
## Call:
## glm(formula = Wigglesworthia ~ Species, data = fig2)
##
## Deviance Residuals:
## Min 1Q Median 3Q Max
## -1.7292 -0.8415 -0.4359 0.3504 3.1233
##
## Coefficients:
## Estimate Std. Error t value Pr(>|t|)
## (Intercept) 5.9954 0.3680 16.291 <2e-16 ***
## SpeciesG. m. morsitans 1.0729 0.4196 2.557 0.0136 *
## ---
## Signif. codes: 0 '***' 0.001 '**' 0.01 '*' 0.05 '.' 0.1 ' ' 1
##
## (Dispersion parameter for gaussian family taken to be 1.6253)
##
## Null deviance: 91.891 on 51 degrees of freedom
## Residual deviance: 81.265 on 50 degrees of freedom
## AIC: 176.79
##
## Number of Fisher Scoring iterations: 2

## data

## data
