# Supplementary material for: Interactions between Glossina pallidipes salivary gland hypertrophy virus and tsetse endosymbionts in wild tsetse populations
Source: Parasit Vectors. 2022 Nov 29;15:447. doi: 10.1186/s13071-022-05536-9 (PMC9707009; doi:10.1186/s13071-022-05536-9)
Supplement: Supplementary file 2 — Additional file 2: Table S1. List of primers used for quantitative PCR (qPCR) analyses in Glossina species. [file 13071_2022_5536_MOESM2_ESM.docx]

**Additional Table S1.** List of Primers used for quantitative PCR (qPCR) analyses in *Glossina* species

| **Target Gene** | **Primer Name** | **Primer Sequence**  **(Listed 5- to -3)** | **Annealing Temperature (^°^C)** | **Amplicon Size (bp)** | **References** |
| --- | --- | --- | --- | --- | --- |
| fliC (flagellin) (*Sodalis*)^*^ | sod-FliCF | GCAGTTTCAGGATACCC | 55 | 508 | [1] |
|  | sod-FliCR | GGCGGAAAATGGTATAG |  |  |  |
| fliC (flagellin) (*Sodalis*)^**^ | sodqPCR-FliCF | GAAGCCACCGATCCTGTAAC | 55 | 508 | [2] |
|  | sodqPCR-FliCR | CAT CTT TGC CCG TAG AAA TCA C |  |  |  |
| Codhoc (*Wigglesworthia*) ^**^ | WiggqPCRcodhocF2 | GACTTGTACGTGATATTTCCAAGC | 60 | 645 | [3] |
|  | WiggqPCRcodhocR2 | GACATCAAATCGCGTTACTGG |  |  |  |
| 16S rRNA (*Wolbachia*)^**^ | Wspec fwd | YATACCTATTCGAAGGGATAG | 60 | 282 | [4, 5] |
|  | Woltse- cyt R | GGATTAGCTTAGCCTCGC |  |  |  |
| thiC (thiamine biosynthesis)  (*Wigglesworthia*)^*^ | WiggqPCRthiCF | GACATCAAATCGCGTTACTGG | 60 | 645 | [6] |
|  | WiggqPCRthiCR | GACTTGTACGTGATATTTCCAAGC' |  |  |  |
| odv-e66 (GpSGHV ORF5)^**^ | qPCRFwda | CAAATGATCCGTCGTGGTAGAA | 60 | 51 | [7, 8] |
|  | qPCRFwda | CAAATGATCCGTCGTGGTAGAA |  |  |  |
| 16S rRNA^*^ | Wspec fwd | YATACCTATTCGAAGGGATAG | 55 | *438* | [4, 9] |
|  | Wspec rev | AGCTTCGAGTGAAACCAATTC |  |  |  |
| odv-e66^*^ | GpSGHV2Fwd | CTTGTCAGCGCCACGTACAT | 55 | 401 | [10] |
|  | GpSGHV2Rev | GCATTCACAGCATCCCAATTTT |  |  |  |

^*^primers used for PCR to prepare the Standard curve

^**^primers used for qPCR

Reference list

1. Toh H, Weiss BL, Perkin SAH, Yamashita A, Oshima K, Hattori M, et al. Massive genome erosion and functional adaptations provide insights into the symbiotic lifestyle of Sodalis glossinidius in the tsetse host. Genome Res. 2006;16:149–56.

2. Weiss BL, Maltz M, Aksoy S. Obligate symbionts activate immune system development in the tsetse fly. J Immunol. 2012;188:3395–403.

3. Rose TM, Schultz ER, Henikoff JG, Pietrokovski S, McCallum CM, Henikoff S. Consensus-degenerate hybrid oligonucleotide primers for amplification of distantly related sequences. Nucleic Acids Res. 1998;26:1628–35.

4. Doudoumis V, Tsiamis G, Wamwiri F, Brelsfoard C, Alam U, Aksoy E, et al. Detection and characterization of *Wolbachia* infections in laboratory and natural populations of different species of tsetse flies (genus *Glossina)*. BMC Micobiology. 2012;12:S3-.

5. Brelsfoard C, Tsiamis G, Falchetto M, Gomulski LM, Telleria E, Alam U, et al. Presence of extensive *Wolbachia* symbiont insertions discovered in the genome of its host *Glossina morsitans morsitans*. PLoS Negl Trop Dis. 2014;8:e2728-.

6. Boucias DG, Kariithi HM, Bourtzis K, Schneider DI, Kelley K, Miller WJ, et al. Transgenerational transmission of the *Glossina pallidipes* hytrosavirus depends on the presence of a functional symbiome. PLoS One. 2013;8:e61150-.

7. Abd-Alla AMM, Cousserans F, Parker A, Bergoin M, Chiraz J, Robinson A. Quantitative PCR analysis of the salivary gland hypertrophy virus (GpSGHV) in a laboratory colony of *Glossina pallidipes*. Virus Res. 2009;139:48–53.

8. Abd-Alla AMM, Salem TZ, Parker AG, Wang Y, Jehle JA, Vreysen MJB, et al. Universal primers for rapid detection of hytrosaviruses. J Virol Methods. 2011;171:280–3.

9. Werren JH, Windsor DM. *Wolbachia* infection frequencies in insects: evidence of a global equilibrium? Proc Biol Sci. 2000;267:1277–85.

10. Abd-Alla A, Bossin H, Cousserans F, Parker A, Bergoin M, Robinson A. Development of a non-destructive PCR method for detection of the salivary gland hypertrophy virus (SGHV) in tsetse flies. J Virol Methods. 2007;139:143–9.
