# Supplementary figures and images for: Interactions between Glossina pallidipes salivary gland hypertrophy virus and tsetse endosymbionts in wild tsetse populations
Source: Parasit Vectors. 2022 Nov 29;15:447. doi: 10.1186/s13071-022-05536-9 (PMC9707009; doi:10.1186/s13071-022-05536-9)

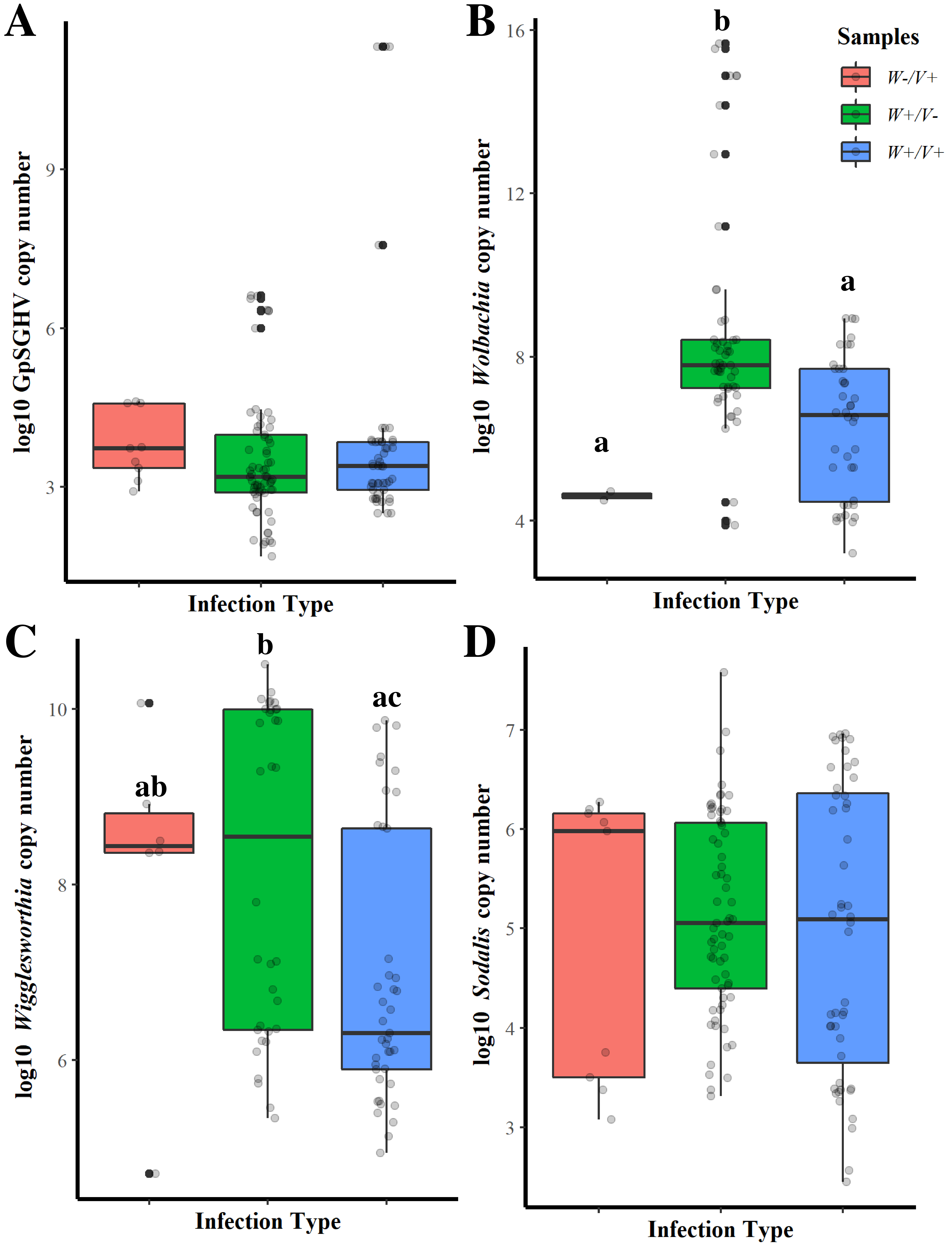

Supplement: Supplementary file 5 — Additional file 5: Figure S2. Density levels of GpSGHV (A), Wolbachia (B), Wigglesworthia (C), and Sodalis (D) determined by qPCR in tsetse flies with different GpSGHV and Wolbachia infection statuses. The copy number was determined by qPCR. Values indicated by a different small letter differ significantly at the 5% level. W+/V+: flies infected with both Wolbachia and GpSGHV; W+/V-: flies infected only with Wolbachia; W-/V-+: flies infected only with GpSGHV. GpSGHV and Wolbachia infection status was determined by conventional PCR as described previously [7,47]. [file 13071_2022_5536_MOESM5_ESM.tif]

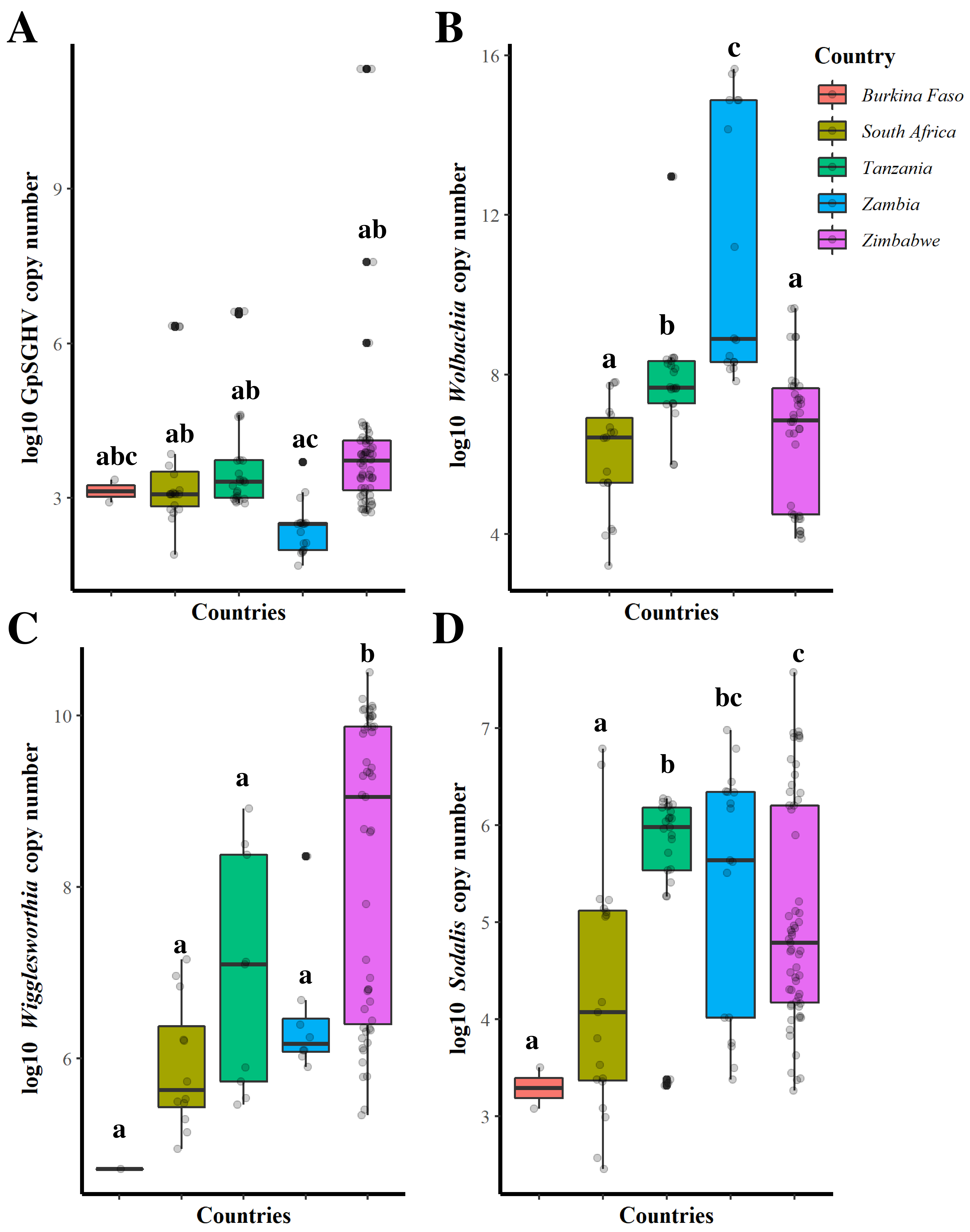

Supplement: Supplementary file 6 — Additional file 6: Figure S3. Density levels of GpSGHV (A), Wolbachia (B), Wigglesworthia (C), and Sodalis (D) in tsetse flies collected from different countries. The copy number was determined by qPCR. Values indicated by a different small letter differ significantly at the 5% level. [file 13071_2022_5536_MOESM6_ESM.tif]

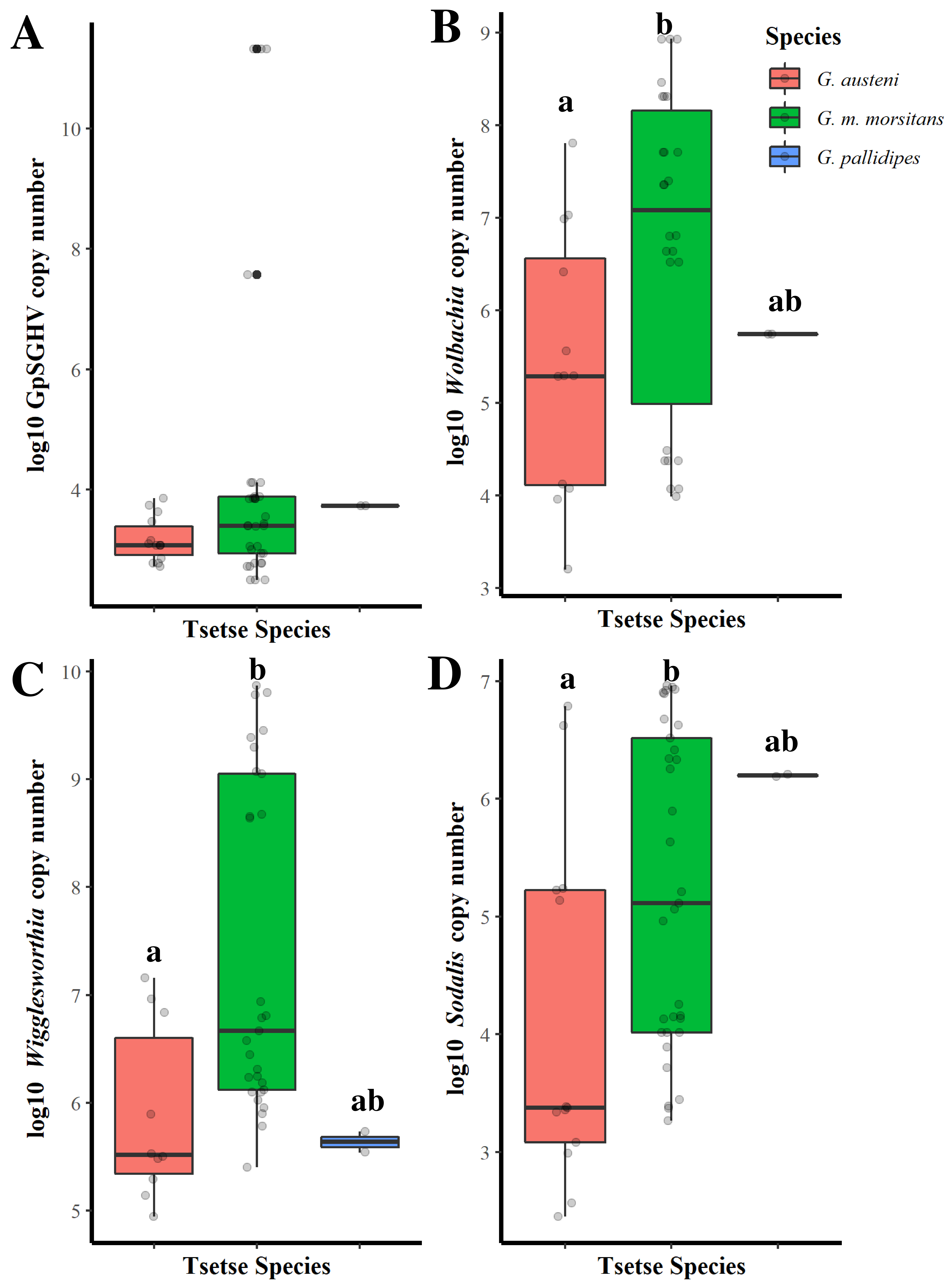

Supplement: Supplementary file 7 — Additional file 7: Figure S4. Impact of GpSGHV and Wolbachia co-infection (W+/V+) on the density levels of GpSGHV (A), Wolbachia (B), Wigglesworthia (C), and Sodalis (D) in different tsetse species. The copy number was determined by qPCR. Values indicated by the same lower case letter do not differ significantly at the 5% level. [file 13071_2022_5536_MOESM7_ESM.tif]
